# Prevalence and attributable health burden of chronic respiratory diseases, 1990–2017: a systematic analysis for the Global Burden of Disease Study 2017

**DOI:** 10.1016/S2213-2600(20)30105-3

**Published:** 2020-06

**Authors:** GBD Chronic Respiratory Disease Collaborators, Joan B Soriano, Parkes J Kendrick, Katherine R Paulson, Vinay Gupta, Elissa M Abrams, Rufus Adesoji Adedoyin, Tara Ballav Adhikari, Shailesh M Advani, Anurag Agrawal, Elham Ahmadian, Fares Alahdab, Syed Mohamed Aljunid, Khalid A Altirkawi, Nelson Alvis-Guzman, Nahla Hamed Anber, Catalina Liliana Andrei, Mina Anjomshoa, Fereshteh Ansari, Josep M Antó, Jalal Arabloo, Seyyede Masoume Athari, Seyyed Shamsadin Athari, Nefsu Awoke, Alaa Badawi, Joseph Adel Mattar Banoub, Derrick A Bennett, Isabela M Bensenor, Kathleen S Sachiko Berfield, Robert S Bernstein, Krittika Bhattacharyya, Ali Bijani, Michael Brauer, Gene Bukhman, Zahid A Butt, Luis Alberto Cámera, Josip Car, Juan J Carrero, Felix Carvalho, Carlos A Castañeda-Orjuela, Jee-Young Jasmine Choi, Devasahayam J Christopher, Aaron J Cohen, Lalit Dandona, Rakhi Dandona, Anh Kim Dang, Ahmad Daryani, Barbora de Courten, Feleke Mekonnen Demeke, Gebre Teklemariam Demoz, Jan-Walter De Neve, Rupak Desai, Samath Dhamminda Dharmaratne, Daniel Diaz, Abdel Douiri, Tim Robert Driscoll, Eyasu Ejeta Duken, Aziz Eftekhari, Hajer Elkout, Aman Yesuf Endries, Ibtihal Fadhil, Andre Faro, Farshad Farzadfar, Eduarda Fernandes, Irina Filip, Florian Fischer, Masoud Foroutan, M.A. Garcia-Gordillo, Abadi Kahsu Gebre, Ketema Bizuwork Gebremedhin, Gebreamlak Gebremedhn Gebremeskel, Kebede Embaye Gezae, Aloke Gopal Ghoshal, Paramjit Singh Gill, Richard F Gillum, Houman Goudarzi, Yuming Guo, Rajeev Gupta, Gessessew Bugssa Hailu, Amir Hasanzadeh, Hamid Yimam Hassen, Simon I Hay, Chi Linh Hoang, Michael K Hole, Nobuyuki Horita, H Dean Hosgood, Mihaela Hostiuc, Mowafa Househ, Olayinka Stephen Ilesanmi, Milena D Ilic, Seyed Sina Naghibi Irvani, Sheikh Mohammed Shariful Islam, Mihajlo Jakovljevic, Amr A Jamal, Ravi Prakash Jha, Jost B Jonas, Zubair Kabir, Amir Kasaeian, Gebremicheal Gebreslassie Kasahun, Getachew Mullu Kassa, Adane Teshome Kefale, Andre Pascal Kengne, Yousef Saleh Khader, Morteza Abdullatif Khafaie, Ejaz Ahmad Khan, Junaid Khan, Jagdish Khubchandani, Young-Eun Kim, Yun Jin Kim, Sezer Kisa, Adnan Kisa, Luke D Knibbs, Hamidreza Komaki, Parvaiz A Koul, Ai Koyanagi, G Anil Kumar, Qing Lan, Savita Lasrado, Paolo Lauriola, Carlo La Vecchia, Tham Thi Le, James Leigh, Miriam Levi, Shanshan Li, Alan D Lopez, Paulo A Lotufo, Fabiana Madotto, Narayan B Mahotra, Marek Majdan, Azeem Majeed, Reza Malekzadeh, Abdullah A Mamun, Navid Manafi, Farzad Manafi, Lorenzo Giovanni Mantovani, Birhanu Geta Meharie, Hagazi Gebre Meles, Gebrekiros Gebremichael Meles, Ritesh G Menezes, Tomislav Mestrovic, Ted R Miller, GK Mini, Erkin M Mirrakhimov, Babak Moazen, Karzan Abdulmuhsin Mohammad, Shafiu Mohammed, Farnam Mohebi, Ali H Mokdad, Mariam Molokhia, Lorenzo Monasta, Masoud Moradi, Ghobad Moradi, Lidia Morawska, Seyyed Meysam Mousavi, Kamarul Imran Musa, Ghulam Mustafa, Mehdi Naderi, Mohsen Naghavi, Gurudatta Naik, Sanjeev Nair, Vinay Nangia, Jobert Richie Nansseu, Javad Nazari, Duduzile Edith Ndwandwe, Ruxandra Irina Negoi, Trang Huyen Nguyen, Cuong Tat Nguyen, Huong Lan Thi Nguyen, Molly R Nixon, Richard Ofori-Asenso, Felix Akpojene Ogbo, Andrew T Olagunju, Tinuke O Olagunju, Eyal Oren, Justin R Ortiz, Mayowa O Owolabi, Mahesh P A, Smita Pakhale, Adrian Pana, Songhomitra Panda-Jonas, Eun-Kee Park, Hai Quang Pham, Maarten J Postma, Hadi Pourjafar, Hossein Poustchi, Amir Radfar, Alireza Rafiei, Fakher Rahim, Mohammad Hifz Ur Rahman, Muhammad Aziz Rahman, Salman Rawaf, David Laith Rawaf, Lal Rawal, Robert C Reiner Jr., Marissa Bettay Reitsma, Leonardo Roever, Luca Ronfani, Elias Merdassa Roro, Gholamreza Roshandel, Kristina E Rudd, Yogesh Damodar Sabde, Siamak Sabour, Basema Saddik, Saeed Safari, Komal Saleem, Abdallah M Samy, Milena M Santric-Milicevic, Bruno Piassi Sao Jose, Benn Sartorius, Maheswar Satpathy, Miloje Savic, Monika Sawhney, Sadaf G Sepanlou, Masood Ali Shaikh, Aziz Sheikh, Mika Shigematsu, Reza Shirkoohi, Si Si, Soraya Siabani, Virendra Singh, Jasvinder A Singh, Michael Soljak, Ranjani Somayaji, Moslem Soofi, Ireneous N Soyiri, Yonatal Mesfin Tefera, Mohamad-Hani Temsah, Berhe Etsay Tesfay, Jarnail Singh Thakur, Alemayehu Toma Toma, Miguel Tortajada-Girbés, Khanh Bao Tran, Bach Xuan Tran, Lorainne Tudor Car, Irfan Ullah, Marco Vacante, Pascual R Valdez, Job F M van Boven, Tommi Juhani Vasankari, Yousef Veisani, Francesco S Violante, Gregory R Wagner, Ronny Westerman, Charles D A Wolfe, Dawit Zewdu Wondafrash, Adam Belay Wondmieneh, Naohiro Yonemoto, Seok-Jun Yoon, Zoubida Zaidi, Mohammad Zamani, Heather J Zar, Yunquan Zhang, Theo Vos

## Abstract

**Background:**

Previous attempts to characterise the burden of chronic respiratory diseases have focused only on specific disease conditions, such as chronic obstructive pulmonary disease (COPD) or asthma. In this study, we aimed to characterise the burden of chronic respiratory diseases globally, providing a comprehensive and up-to-date analysis on geographical and time trends from 1990 to 2017.

**Methods:**

Using data from the Global Burden of Diseases, Injuries, and Risk Factors Study (GBD) 2017, we estimated the prevalence, morbidity, and mortality attributable to chronic respiratory diseases through an analysis of deaths, disability-adjusted life-years (DALYs), and years of life lost (YLL) by GBD super-region, from 1990 to 2017, stratified by age and sex. Specific diseases analysed included asthma, COPD, interstitial lung disease and pulmonary sarcoidosis, pneumoconiosis, and other chronic respiratory diseases. We also assessed the contribution of risk factors (smoking, second-hand smoke, ambient particulate matter and ozone pollution, household air pollution from solid fuels, and occupational risks) to chronic respiratory disease-attributable DALYs.

**Findings:**

In 2017, 544·9 million people (95% uncertainty interval [UI] 506·9–584·8) worldwide had a chronic respiratory disease, representing an increase of 39·8% compared with 1990. Chronic respiratory disease prevalence showed wide variability across GBD super-regions, with the highest prevalence among both males and females in high-income regions, and the lowest prevalence in sub-Saharan Africa and south Asia. The age-sex-specific prevalence of each chronic respiratory disease in 2017 was also highly variable geographically. Chronic respiratory diseases were the third leading cause of death in 2017 (7·0% [95% UI 6·8–7·2] of all deaths), behind cardiovascular diseases and neoplasms. Deaths due to chronic respiratory diseases numbered 3 914 196 (95% UI 3 790 578–4 044 819) in 2017, an increase of 18·0% since 1990, while total DALYs increased by 13·3%. However, when accounting for ageing and population growth, declines were observed in age-standardised prevalence (14·3% decrease), age-standardised death rates (42·6%), and age-standardised DALY rates (38·2%). In males and females, most chronic respiratory disease-attributable deaths and DALYs were due to COPD. In regional analyses, mortality rates from chronic respiratory diseases were greatest in south Asia and lowest in sub-Saharan Africa, also across both sexes. Notably, although absolute prevalence was lower in south Asia than in most other super-regions, YLLs due to chronic respiratory diseases across the subcontinent were the highest in the world. Death rates due to interstitial lung disease and pulmonary sarcoidosis were greater than those due to pneumoconiosis in all super-regions. Smoking was the leading risk factor for chronic respiratory disease-related disability across all regions for men. Among women, household air pollution from solid fuels was the predominant risk factor for chronic respiratory diseases in south Asia and sub-Saharan Africa, while ambient particulate matter represented the leading risk factor in southeast Asia, east Asia, and Oceania, and in the Middle East and north Africa super-region.

**Interpretation:**

Our study shows that chronic respiratory diseases remain a leading cause of death and disability worldwide, with growth in absolute numbers but sharp declines in several age-standardised estimators since 1990. Premature mortality from chronic respiratory diseases seems to be highest in regions with less-resourced health systems on a per-capita basis.

**Funding:**

Bill & Melinda Gates Foundation.

## Introduction

Chronic respiratory diseases are diseases of the airways and other structures of the lung,^1^ and are among the leading causes of morbidity and mortality worldwide.^2^ Some of the most common chronic respiratory diseases are asthma, chronic obstructive pulmonary disease (COPD), and occupational lung diseases. These disease entities are important contributors to the rising burden of non-communicable diseases (NCDs) globally.^2^

The WHO Global Alliance against Chronic Respiratory Diseases was established with the goal of reducing the burden of chronic respiratory diseases, towards a world in which all people breathe freely, and focuses on the needs of people with chronic respiratory diseases in low-income and middle-income countries.^3^ Risk factors for chronic respiratory diseases are common: at least 2 billion people are exposed to the toxic effects of biomass fuel use, 1 billion are exposed to outdoor air pollution, and 1 billion are smokers who expose a near-equal magnitude of people to the ill-effects of second-hand smoke.^4^ Each year, it is estimated that 4 million people die prematurely from chronic respiratory diseases.^5^ Although occupational respiratory conditions are a well characterised risk factor, their magnitude is ill-defined; on the basis of the few analyses that exist, around 2 million work-related deaths annually are estimated to occur because of work-related exposures relevant to respiratory conditions.^6^

**Research in context**

**Evidence before this study**

Although attempts were made to assess the burden of chronic obstructive pulmonary disease (COPD) and asthma in the Global Burden of Disease Study (GBD) 2015, there have been no reports summarising the burden of all chronic respiratory diseases or a comprehensive compilation of prevalence, deaths, and disability-adjusted life-years (DALYs) for all chronic respiratory diseases worldwide over an extended period. Such monitoring is needed to comply with the pre-stated aims of the Sustainable Development Goals, which specifically call for a one-third reduction in premature mortality from non-communicable diseases (NCDs) such as chronic respiratory diseases.

**Added value of this study**

As part of GBD 2017, we show that prevalence and mortality of chronic respiratory diseases are still rising in absolute numbers as a result of diverse risk factors, particularly in affluent countries. Our finding that nearly 545 million individuals currently live with a chronic respiratory condition, representing 7·4% of the world's population, provides additional evidence on the large health contribution of chronic respiratory diseases to premature morbidity and mortality. However, both age-standardised mortality and DALY rates per 100 000 people show sharp declines between 1990 and 2017. In males and females, disability remains highest in south Asia, where premature mortality from chronic respiratory diseases is highest. Risk factor estimates confirm that smoking remains the predominant risk factor for death and disability due to chronic respiratory diseases across all regions globally for men. Among women, exceptions exist, as household air pollution from solid fuels and ambient particulate matter were the leading risk factors in areas of Asia and Africa.

**Implications of all the available evidence**

COPD, asthma, and other chronic respiratory diseases—including interstitial lung disease, sarcoidosis, and pneumoconiosis—are important contributors to the global burden of NCDs. Although much of this burden is either preventable or treatable with affordable interventions, these diseases have received less attention than other NCDs. Identifying these up-to-date epidemiological trends in each individual chronic respiratory disease is key to informing policy decisions on resource allocation and ensuring adequate training of national health-care workforces. Our study shows that chronic respiratory diseases remain a leading cause of death and disability worldwide.

Lung diseases in children under 5 years of age, counting both acute infectious processes and chronic conditions, are among the most common causes of death.^7^ Notably, asthma is the most common chronic condition, affecting about 14% of children globally with rising prevalence.^8^

The Forum of International Respiratory Societies considers pneumonia, asthma, COPD, lung cancer, and tuberculosis the five most important lung diseases worldwide from a prevalence standpoint.^5^ Although chronic respiratory diseases are not curable, various forms of treatment can help to control symptoms, increase patients' quality of life, and prevent adverse outcomes (including exacerbations) that are associated with substantial morbidity, increased health-care use, disability, and risk of death.^3^ Infants and young children are particularly susceptible to this continuum of poor clinical outcomes.^1^

Health-care costs for respiratory diseases are an increasing burden on the economies of all nations. In 2019, among 28 EU member states, costs of about €380 billion annually were attributable to the care of patients with chronic respiratory diseases alone. Included in this estimate are the costs of direct primary and inpatient health care (the latter estimated to be at least €55 billion), the costs of lost productivity (at least €42 billion), and the monetised value of disability-adjusted life-years (DALYs) lost (at least €280 billion).^9^

Despite these extensive health and economic consequences, comprehensive published data on the prevalence and distribution of chronic respiratory diseases at a global scale are lacking.^10^, ^11^, ^12^, ^13^ The primary objective of our analysis was to address this evidence gap, by estimating the prevalence and disease burden in terms of deaths, DALYs, and DALY components—years lived with disability (YLDs) and years of life lost (YLLs)—for chronic respiratory diseases as part of the Global Burden of Diseases, Injuries, and Risk Factors Study (GBD) 2017. We also report on the burden of chronic respiratory diseases attributable to a series of causal risk factors and characterise the variability in our findings across the seven GBD super-regions. Our overarching aim is to inform policy makers on the persistent and, in some cases, rising burden of chronic respiratory diseases, especially given the significance of these findings to achieving the health-related Sustainable Development Goals (SDGs) by 2030.

## Methods

### Overview

Methods employed in the GBD 2017 have been extensively reported elsewhere.^14^, ^15^, ^16^, ^17^ Attributable deaths, prevalence, and DALYs were estimated for up to 359 diseases and injuries, as well as 84 behavioural, environmental and occupational, and metabolic risks or clusters of risks, for 195 countries and territories, by 23 age groups and by sex, from 1990 to 2017.^18^ The generic GBD protocol and the visualisation tool are accessible online.

As per the GBD Data Dictionary, chronic respiratory diseases include the following five categories: asthma, COPD, interstitial lung disease and pulmonary sarcoidosis, pneumoconiosis (including silicosis, asbestosis, coal-worker pneumoconiosis, and other pneumoconiosis), and other chronic respiratory diseases. The other chronic respiratory diseases category comprised obstructive sleep apnoea; vasomotor and allergic rhinitis; chronic rhinitis; nasopharyngitis and pharyngitis; chronic sinusitis; nasal polyposis; other and unspecified disorders of the nose and nasal sinuses; chronic diseases of the tonsils and adenoids; chronic laryngitis and laryngotracheitis; diseases of the vocal cords and larynx, not elsewhere classified; other diseases of the upper respiratory tract; airway disease due to specific organic dust; hypersensitivity pneumonitis due to organic dust; respiratory conditions due to inhalation of chemicals, gases, fumes, and vapours; respiratory conditions due to other external agents; respiratory conditions due to other specified external agents; pulmonary eosinophilia, not elsewhere classified; pleural effusion in conditions classified elsewhere; pleural plaque with presence of asbestos; and pleural plaque without asbestos. Note that infectious diseases such as tuberculosis were not included, even though it is one of the most prevalent respiratory conditions.^5^

The source data, including case definitions, epidemiological estimators, exposures, risk estimates, are freely available at the Global Health Data Exchange (GHDx). Method write-ups for each cause and risk factor are available in appendix 1 (pp 5, 33).

This study is compliant with the Guidelines for Accurate and Transparent Health Estimates Reporting.^19^

### Prevalence

The prevalence of chronic respiratory diseases was estimated by location, age, sex, and year using DisMod-MR version 2.1, a Bayesian regression analytical tool used in the GBD study which synthesises variable data sources to produce internally consistent estimates of incidence, prevalence, remission, and excess mortality.^20^ The major data inputs for estimating the prevalence of chronic respiratory diseases were population representative surveys, smaller prevalence studies in the literature identified by a systematic review, and hospital claims data.

The estimation included ascertainment of the severity distribution of sequelae, incorporation of disability weights, and comorbidity adjustment of sequelae. Disability weights (appendix 1 p 61), which quantify the relative severity of the sequela on a scale of 0–1, were derived from nine population surveys and an open-access internet survey using pairwise comparison methods.^21^ Notably, prevalence data for interstitial lung disease, pulmonary sarcoidosis, and pneumoconiosis were derived primarily from hospital inpatient and insurance claims data.

YLDs were calculated by multiplying the prevalence of each sequela by its disability weight for the corresponding health state plus a comorbidity adjustment. The estimation of YLDs consists of three main steps: establishing the prevalence and incidence of causes and sequelae (health states caused by a disease, such as blindness caused by diabetes) related to disability, assigning levels of severity to those disabilities, and combining prevalence and incidence with severity, adjusted for comorbidity, into one comprehensive measure of non-fatal health loss. We used an updated and extensive body of literature studies, survey data, surveillance data, inpatient admission records, outpatient visit records, and health insurance claims, and additionally used results from cause of death models to inform estimates. We then used those data, along with tools such as DisMod-MR 2.1, to generate estimates of the prevalence and incidence of disability-causing sequelae, ensuring consistency between rates of incidence, prevalence, remission, and cause of death for each condition.

### Mortality

All-cause mortality rates were derived from vital registration systems, censuses, and surveys, and were analysed with demographic methods to correct for incompleteness. Causes of death from an extensive database of vital registration and verbal autopsy data were analysed with GBD's Cause of Death Ensemble model tool to calculate a large number of mixed effects or spatiotemporal Gaussian process regression models of rates or cause fractions with varying combinations of predictive covariates. Predictive validity testing determined the optimal ensemble of models. Covariates included smoking prevalence, cigarettes per capita, the proportion exposed to household air pollution, mean fine particulate matter (with a diameter <2·5 μm [PM_2·5_]) from outdoor air pollution, a scalar of the combined exposure to risks for individual chronic respiratory diseases, and Socio-demographic Index. As the sensitivity of verbal autopsy algorithms to detect specific chronic respiratory diseases is poor, we excluded verbal autopsy from all but the total chronic respiratory disease model. Estimates for individual chronic respiratory diseases were constrained to the estimates for total chronic respiratory disease, and all individual causes were constrained to the all-cause mortality rates derived from demographic estimation.

YLLs were obtained by multiplying counts of deaths for a cause by the remaining life expectancy in GBD's standard life table on the basis of lowest observed mortality rates at each age in any population over 5 million people. DALYs are the sum of YLLs and YLDs. We present both crude estimates, and age-standardised rates in appendix 1 (p 62), as relevant. Age-standardised rates were based on the GBD global reference population (available at GHDx).

### Risk estimation

Estimates were composed of six risk factors for COPD (smoking, second-hand smoke, household air pollution, ambient particulate matter, ozone, and occupational particulates, which include coal dust), two risk factors for asthma (smoking and occupational exposures), and three risk factors for pneumoconiosis (occupational particulates, occupational silica, and occupational asbestos). Sufficient evidence of causality, availability of exposure data, potential for behavioural and other modifications, and health policy interest are criteria for choosing risks and associated outcomes in GBD. Population-attributable fractions of disease outcomes were estimated from exposure data, relative risks of outcomes, and a theoretical minimum exposure level. Population surveys were the main source of exposure data on smoking, second-hand smoke, and household air pollution. Exposure to ambient particulate matter (defined as the population-weighted annual average mass concentration of PM_2·5_ in 1 m^3^ of air) was estimated from satellite data on aerosols in the atmosphere and calibrated to observations from ground monitors. Exposure to ozone was based on a chemical transport model of satellite data.^22^ Occupational exposures were based on the proportion of the working population exposed to asthmagens and particulates based on distribution of the population in nine occupational groups as reported by the International Labour Organization.^23^ Relative risks were derived from pooled estimates in meta-analyses of cohort studies. The theoretical minimum exposure level was set as zero for smoking, second-hand smoke, and the occupational exposures. For household air pollution, the minimum was defined as no household reporting use of solid fuel for cooking. For ambient particulate matter, the minimum was set as a uniform distribution between the lowest and fifth percentile exposure level from all data sources. For ozone, the minimum was set as a uniform distribution between the lowest and fifth percentile exposure measured in the American Cancer Society Cancer Prevention Study-II.^24^ Unlike disease estimates, which are mutually exclusive and collectively exhaustive in GBD, risk estimates are based on a counterfactual analysis and are therefore not additive. Estimates of combinations of risks take mediation into account based on the difference in relative risks from cohort and trial data that did and did not control for another risk as a confounder. After adjustment for mediation, risks were combined using a multiplicative function to avoid the sum of risks exceeding the total amount of disease.^25^ Additional details on the estimation process for COPD and asthma risks can be found elsewhere.^10^

### Statistical analysis

Uncertainty was estimated by recalculating every outcome of interest 1000 times, drawing from distributions of the sampling error around input data, corrections for measurement error, and estimates of residual non-sampling error and, in the case of cause of death estimates, model selection. Uncertainty intervals (UIs) were defined as the 2·5th and 97·5th values of the posterior distributions. Differences between estimates were computed at the 1000-draw level and reported as significant if more than 95% of values for the difference are either positive or negative. All code is freely available at GHDx.

## Results

**Figure 1:**
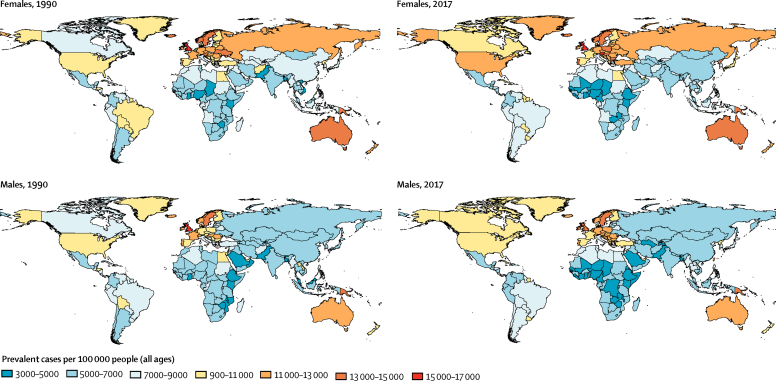
All-age chronic respiratory disease prevalence by country in 1990 and 2017

In 2017, an estimated 544· 9 million (95% UI 506·9–584·9) individuals worldwide had a chronic respiratory disease, equivalent to a 39·8% increase compared with the number of individuals affected in 1990 (389·7 million [362·9–416·3]; figure 1). The global prevalence in 2017 was around 7·1% (95% UI 6·6–7·7). Chronic respiratory diseases were most prevalent across the GBD high-income super-region, at 10·6% (9·9–11·3), up from 9·7% (9·1–10·3) in 1990 (table 1). By contrast, the lowest prevalence was observed in sub-Saharan Africa (5·1% [4·5–5·8]) and south Asia (5·5% [5·1–6·0]). Latin America and the Caribbean had the largest decline (–0·80 percentage points) in chronic respiratory disease prevalence over the study period, from 8·1% in 1990 to 7·3% in 2017. Sub-Saharan Africa and the central Europe, eastern Europe, and central Asia super-region also saw declines in the prevalence of chronic respiratory diseases (table 1).

**Table 1:** Point prevalence estimates of each chronic respiratory disease category by super-region in 1990 and 2017

	**Global**		**Central Europe, eastern Europe, and central Asia**		**High income**		**Latin America and Caribbean**		**North Africa and Middle East**		**South Asia**		**Southeast Asia, east Asia, and Oceania**		**Sub-Saharan Africa**	
	1990	2017	1990	2017	1990	2017	1990	2017	1990	2017	1990	2017	1990	2017	1990	2017
All chronic respiratory diseases	7·2240% (6·7278–7·7178)	7·1318% (6·6349–7·6547)	9·4698% (8·9050–10·0558)	9·1695% (8·5190–9·8830)	9·7207% (9·1347–10·3371)	10·5939% (9·9450–11·2672)	8·1434% (7·2581–9·0943)	7·3431% (6·6467–8·0598)	7·5713% (6·8684–8·3425)	7·7191% (7·0586–8·4066)	5·1293% (4·7956–5·5105)	5·5420% (5·1582–5·9500)	6·9019% (6·4080–7·4275)	7·0559% (6·5256–7·5955)	5·5665% (4·9793–6·1801)	5·1371% (4·5452–5·7772)
Chronic obstructive pulmonary disease	3·7051% (3·4124–4·0088)	3·9186% (3·5211–4·3201)	6·1588% (5·6361–6·6986)	6·0944% (5·4141–6·8561)	4·4068% (4·0763–4·7459)	5·9003% (5·3054–6·5329)	2·6773% (2·4761–2·8940)	3·3298% (2·9837–3·7173)	2·5562% (2·2937–2·8572)	3·1329% (2·8077–3·4923)	3·2030% (2·8847–3·5268)	3·5831% (3·2125–3·9711)	4·0542% (3·7328–4·4057)	4·2839% (3·8589–4·7329)	1·8222% (1·6283–2·0244)	1·5682% (1·3977–1·7538)
Asthma	3·9054% (3·4540–4·3882)	3·5689% (3·1712–3·9880)	3·8883% (3·5006–4·2786)	3·5087% (3·1157–3·9314)	5·8365% (5·2695–6·4139)	5·2880% (4·7870–5·8009)	5·8218% (4·8748–6·8155)	4·2885% (3·6572–4·9709)	5·3714% (4·6585–6·1478)	4·9506% (4·3343–5·6223)	2·2982% (2·0488–2·5586)	2·3527% (2·0916–2·6258)	3·1978% (2·7815–3·6500)	3·0848% (2·7104–3·4697)	3·9219% (3·3666–4·5551)	3·7072% (3·1200–4·3518)
Interstitial lung disease and pulmonary sarcoidosis	0·0617% (0·0563–0·0679)	0·0816% (0·0741–0·0896)	0·2142% (0·1929–0·2379)	0·2110% (0·1883–0·2359)	0·1223% (0·1113–0·1336)	0·1975% (0·1806–0·2147)	0·0275% (0·0250–0·0303)	0·0452% (0·0410–0·0498)	0·0185% (0·0166–0·0206)	0·0267% (0·0240–0·0299)	0·0284% (0·0256–0·0315)	0·0432% (0·0387–0·0480)	0·0363% (0·0325–0·0404)	0·0758% (0·0678–0·0846)	0·0412% (0·0370–0·0459)	0·0393% (0·0352–0·0437)
Pneumoconiosis	0·0054% (0·0048–0·0061)	0·0069% (0·0062–0·0078)	0·0065% (0·0058–0·0074)	0·0067% (0·0060–0·0076)	0·0041% (0·0036–0·0047)	0·0057% (0·0051–0·0064)	0·0034% (0·0030–0·0039)	0·0045% (0·0039–0·0052)	0·0028% (0·0023–0·0032)	0·0039% (0·0033–0·0047)	0·0020% (0·0017–0·0023)	0·0023% (0·0020–0·0028)	0·0100% (0·0087–0·0115)	0·0153% (0·0134–0·0175)	0·0017% (0·0015–0·0020)	0·0016% (0·0014–0·0019)

The “other chronic respiratory diseases” category is not shown because the Global Burden of Diseases Study does not estimate prevalence for such a category, and thus all prevalence values would be 0.

COPD remained the most prevalent disease-specific chronic respiratory disease worldwide in 2017, accounting for 55·1% of chronic respiratory disease prevalence among men and 54·8% among women globally. The relative increase in overall prevalence was 5·9% (1·1–9·8) between 1990 and 2017. COPD was most prevalent in the central Europe, eastern Europe, and central Asia super-region, at 6·1% in 2017. The high-income super-region had a similar prevalence of 5·9%, representing an increase of 1·5 percentage points from the prevalence in 1990 (4·4%), which was the greatest relative difference over the study period. Asthma remained the second most prevalent chronic respiratory disease worldwide, although, in aggregate, its prevalence had decreased since 1990, from 3·9% (3·5–4·4) to 3·6% (3·2–4·0; table 1).

By sex, chronic respiratory disease prevalence was highest for both males and females in the high-income region (figure 1). At the country level, the UK had the highest rates in both 1990 and 2017 (country estimates, overall and by sex, are provided in appendix 2).

**Figure 2:**
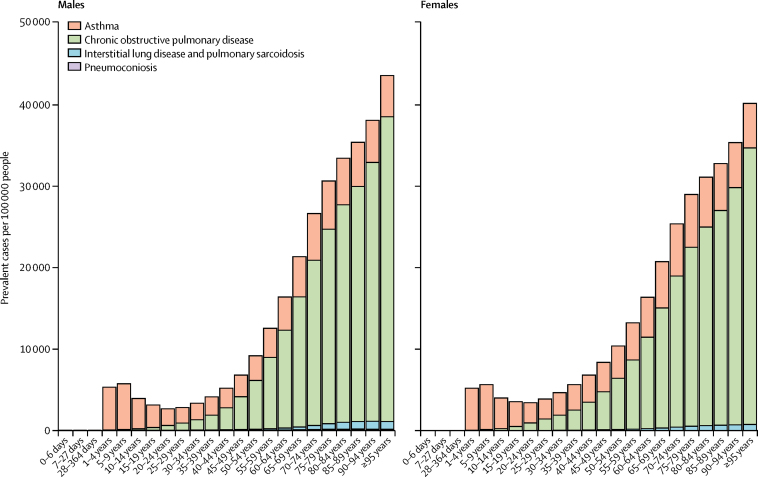
Global age-sex-specific prevalence of chronic respiratory diseases by disease category in 2017

The age-sex-specific prevalence of each chronic respiratory disease in 2017 was highly variable (figure 2). Asthma prevalence peaked at 5–9 years of age among boys and girls, and was greater among boys than among girls up to 9 years of age. However, from age 10 years onwards, asthma prevalence was consistently higher among females than males. From the onset of adulthood to old age, COPD increased monotonically, while the contributions of interstitial lung disease, pulmonary sarcoidosis, and other chronic respiratory diseases decreased relative to all chronic respiratory diseases (figure 2). Interstitial lung disease and pulmonary sarcoidosis were more prevalent among males than among females (appendix 2).

The age-standardised prevalence of chronic respiratory diseases was 8157·75 (7663·98–8662·19) cases per 100 000 in 1990 and 6991·55 (6496·73–7516·36) cases per 100 000 in 2017, representing a decrease of 14·3% (appendix 1 p 62).

There were 3 914 196 (3 790 578–4 044 819) deaths due to chronic respiratory diseases in 2017 globally, an increase of 18·0% since 1990 (3 317 205 [3 011 594–3 425 405] deaths). In total, chronic respiratory diseases accounted for 51·23 (49·61–52·94) deaths per 100 000 individuals in 2017, 56·45 (54·32–58·08) per 100 000 males and 45·97 (42·73–49·34) per 100 000 females (table 2). These deaths, in aggregate, accounted for 7·0% (6·8–7·2) of total all-cause deaths globally, ranking chronic respiratory diseases as the third leading cause of death in 2017, just behind cardiovascular diseases (31·8% [31·4–32·2] of all deaths) and neoplasms (17·1% [16·8–17·3]). However, age-standardised mortality rates of chronic respiratory diseases decreased by 42·6% between 1990 (89·5 [81·2–92·5] deaths per 100 000) and 2017 (51·4 [49·75–53·1] deaths per 100 000).

**Table 2:** Chronic respiratory disease-attributable deaths and DALYs per 100 000 individuals and as a proportion of all-cause deaths and DALYs, respectively, across all super regions, 2017

	**Overall**				**Males**				**Females**			
	Death rate per 100 000	Proportion of all-cause deaths, %	DALY rate per 100 000	Proportion of all-cause DALYs, %	Death rate per 100 000	Proportion of all-cause deaths, %	DALY rate per 100 000	Proportion of all-cause DALYs, %	Death rate per 100 000	Proportion of all-cause deaths, %	DALY rate per 100 000	Proportion of all-cause DALYs, %
All chronic respiratory diseases	51·23 (49·61–52·94)	7·00% (6·76–7·23)	1470·03 (1369·68–1566·56)	4·50% (4·20–4·78)	56·45 (54·32–58·08)	7·12% (6·89–7·30)	1529·43 (1432·75–1624·22)	4·37% (4·12–4·60)	45·97 (42·73–49·34)	6·85% (6·37–7·34)	1410·18 (1288·53–1520·29)	4·65% (4·27–5·03)
Asthma	6·48 (4·43–8·39)	0·88% (0·60–1·14)	297·92 (236·69–370·88)	0·91% (0·76–1·09)	6·30 (3·72–8·85)	0·79% (0·47–1·11)	287·50 (220·90–368·68)	0·82% (0·65–1·02)	6·66 (4·55–8·68)	0·99% (0·68–1·29)	308·43 (237·74–388·97)	1·02% (0·82–1·23)
Chronic obstructive pulmonary disease	41·85 (39·64–43·96)	5·72% (5·43–5·97)	1068·02 (994·47–1135·50)	3·27% (2·96–3·56)	46·68 (43·62–49·25)	5·89% (5·50–6·20)	1128·21 (1045·99–1202·19)	3·22% (2·93–3·49)	36·99 (33·63–39·85)	5·51% (5·00–5·91)	1007·37 (916·25–1088·81)	3·33% (2·95–3·71)
Interstitial lung diseases and pulmonary sarcoidosis	1·93 (1·50–2·37)	0·26% (0·20–0·32)	44·04 (36·19–53·43)	0·13% (0·11–0·16)	2·09 (1·60–2·73)	0·26% (0·20–0·35)	47·93 (38·75–62·32)	0·14% (0·11–0·18)	1·78 (1·19–2·37)	0·26% (0·18–0·35)	40·13 (30·41–52·65)	0·13% (0·10–0·17)
Pneumoconiosis	0·28 (0·27–0·30)	0·04% (0·04–0·04)	6·64 (6·18–7·17)	0·02% (0·02–0·02)	0·50 (0·47–0·53)	0·06% (0·06–0·07)	11·82 (10·98–12·75)	0·03% (0·03–0·04)	0·06 (0·05–0·07)	0·01% (0·01–0·01)	1·42 (1·20–1·66)	0·00% (0·00–0·01)
Other chronic respiratory diseases	0·68 (0·60–0·78)	0·09% (0·08–0·11)	53·40 (47·16–59·63)	0·16% (0·15–0·18)	0·89 (0·76–1·06)	0·11% (0·10–0·13)	53·97 (47·38–61·67)	0·15% (0·14–0·18)	0·48 (0·39–0·56)	0·07% (0·06–0·08)	52·83 (45·68–59·90)	0·17% (0·15–0·20)

Data are point estimate (95% uncertainty interval). DALYs=disability-adjusted life-years.

**Figure 3:**
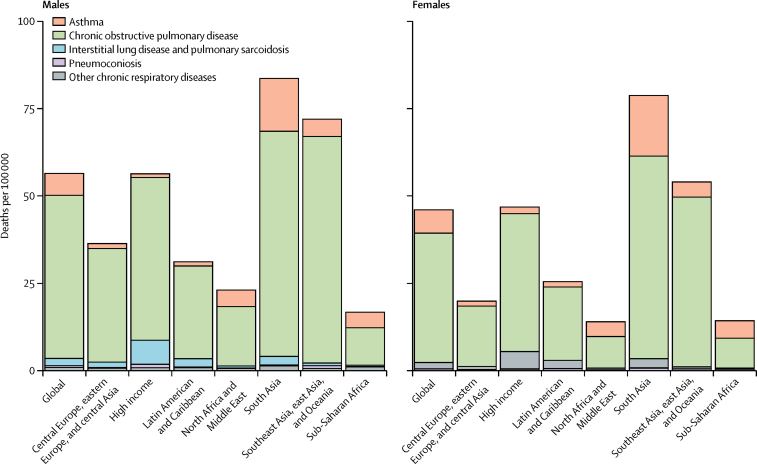
All-age mortality rates of chronic respiratory diseases by disease category across super-regions in 2017

COPD was the most common cause of chronic respiratory disease-attributable deaths, at 41·9 deaths per 100 000 individuals (5·7% of total all-cause deaths; figure 3, table 2). 46·7 (43·6–49·3) deaths per 100 000 among men and 37·0 (33·6–39·9) per 100 000 among women were attributable to COPD. Worldwide, asthma was the second leading cause of death among chronic respiratory diseases.

Geographically, deaths attributable to chronic respiratory disease were most frequent in the south Asia super-region (81·2 deaths [75·4–86·3] per 100 000 individuals) in 2017, and least frequent in sub-Saharan Africa (15·5 deaths [14·4–17·0] per 100 000 Individuals; figure 3). COPD was the most common cause of deaths attributable to chronic respiratory disease in each individual super-region. The second most common cause of chronic respiratory deaths was asthma, in all but three super-regions. The exceptions were interstitial lung disease and pulmonary sarcoidosis in the high-income region (5·9 deaths [4·0–6·6] per 100 000), Latin America and the Caribbean region (0·3 deaths [0·2–0·4] per 100 000), and central Europe, eastern Europe, and central Asia super-regions (1·2 deaths [1·0–1·7] per 100 000; figure 3).

**Figure 4:**
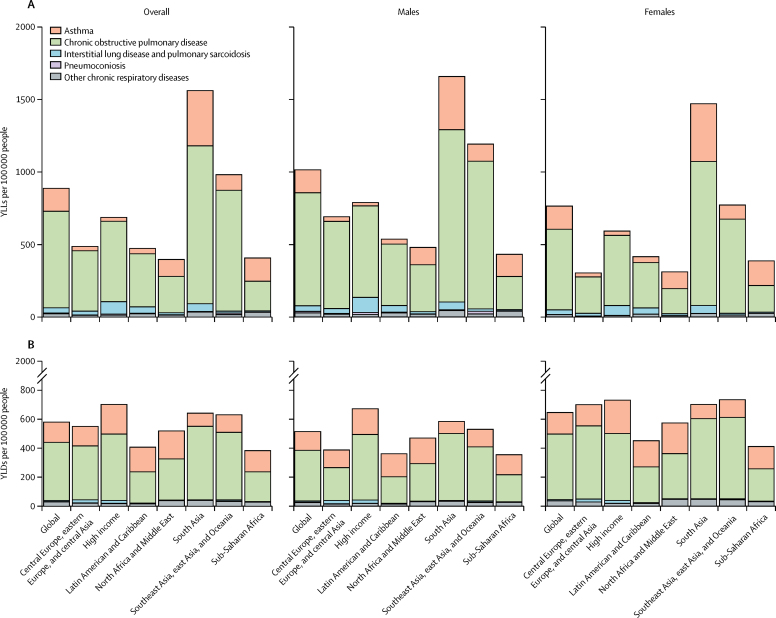
Chronic respiratory disease-attributable YLL rates (A) and YLD rates (B) across super-regions in 2017 YLL=years of life lost. YLD=years lived with disability.

Overall, there were 112 316 763 DALYs (95% UI 104 649 690–119 692 783) due to chronic respiratory diseases in 2017 globally, an increase of 13·3% since 1990 (99 103 908 DALYs [91 717 116–104 764 799]). In south Asia, the majority of DALYs were the result of the high YLL rate in this region (1567·1 YLLs [1470·9–1663·4] per 100 000 people; figure 4A), while the YLD rate accounted for a minority share of 641·6 YLDs (541·1–727·9) per 100 000 people (figure 4B). In the high-income super-region, by contrast, the composition of DALYs was comparatively more balanced, with 688·8 YLLs and 702·0 YLDs per 100 000 people (figure 4). When considering specific diseases, similar findings were observed with respect to morbidity: COPD accounted for the majority of chronic respiratory disease-associated DALYs, while asthma was the second leading cause of morbidity (figure 4). Similar to prevalence and mortality, age-standardised DALY rates for chronic respiratory diseases decreased by 38·2% between 1990 (2304·36 DALYs [2138·52–2422·37] per 100 000 people) and 2017 (1422·89 DALYs [1326·65–1517·23] per 100 000 people).

**Figure 5:**
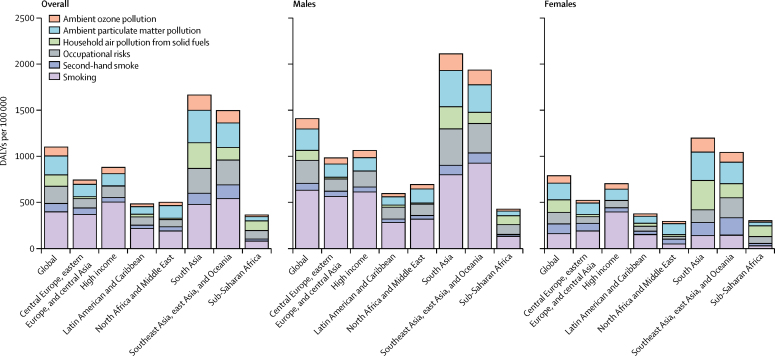
Chronic respiratory disease-attributable DALY rates by risk factor across super-regions in 2017 DALY=disability-adjusted life-year.

Smoking was the most prevalent risk factor for chronic respiratory diseases worldwide, responsible for 396·4 DALYs (355·1–435·5) per 100 000 people (27·0% [24·5–29·6] of chronic respiratory disease-attributable DALYs; figure 5). Smoking accounted for the largest fraction of all chronic respiratory disease morbidity in all super-regions except sub-Saharan Africa (80·5 [67·4–94·2] DALYs per 100 000 people; 10·2% [8·5–11·9]), where household air pollution (105·5 DALYs [77·0–132·7] per 100 000 people) and occupational risks (89·9 DALYs [79·6–100·7] per 100 000 people) were more prominent factors. These findings were not consistent across sexes, however, as exposure to household air pollution from solid fuels was the leading risk factor among women globally, and in south Asia and sub-Saharan Africa. In addition, among women, household air pollution from solid fuels represented the leading risk factor in the south Asia and the southeast Asia, east Asia, and Oceania super-regions. In these two super-regions smoking accounted for the lowest fraction for women but the greatest fraction for men. Smoking-attributable DALYs were the lowest in the north Africa and Middle East and the sub-Saharan Africa super-regions (figure 5; appendix 3).

## Discussion

This study shows that chronic respiratory diseases remain a leading cause of disability and death worldwide, with the highest prevalence (10–11% of the population or higher) observed in high-income regions, a finding unchanged since first estimated in 1990. In south Asia, although crude prevalence is lower than that in most other super-regions, morbidity and mortality attributable to chronic respiratory diseases are the highest in the world. Much of this burden is the result of premature mortality. In general, progress has been modest in alleviating chronic respiratory disease burden over the past 30 years, with the largest reductions observed in Latin America. COPD remains the most prevalent of chronic respiratory diseases globally among men and women, with crude prevalence rising globally in absolute terms. This change is probably due to population ageing and longer life expectancies, particularly in high-income areas, although improved diagnosis cannot be ruled out. However, from 1990 to 2017, the prevalence, mortality, and DALY rates per 100 000 people dropped by 14·3%, 42·6%, and 38·2%, respectively, if we adjust for population growth and ageing. These sharp declines might be the result of a long list of factors, including global or regional success in tobacco control measures, paired with reductions in environmental pollution in cities, more electric cars, cleaner working environments, better information and prevention with regard to allergens, better treatment of respiratory and non-respiratory comorbidities, and reductions of universally high rates of underdiagnosis of chronic respiratory diseases. Although smoking is the leading risk factor for DALYs related to chronic respiratory diseases across all regions for men, other risk factors such as household and ambient air pollution have substantial effects on women across Asia and Africa.

Interpretation of counts and absolute rates is straightforward. We also present the results as age-standardised rates in appendix 1 (pp 62–66). Crude estimates provide the real situation in each country, which is useful for policy makers and practising physicians, whereas age-standardised estimates allow comparisons over time and between countries after adjusting for the differences in the age structure and population growth of the population for a specific year. For grasping the current burden of all chronic respiratory diseases in the short period from 1990 to 2017, representing only one generation, we suggest the overall picture of counts and crude rates to be simpler and more useful. However, for longer periods, the use of age-standardised rates is much more appropriate, and even necessary. Predicted values for all-age standardised mortality for COPD by GBD super-region in 2040 are already available through GBD Foresight.^26^ In addition, all estimates for each of the 195 individual countries or territories are available in appendix 2.

Given the high mortality rates due to chronic respiratory diseases in south Asia, increased attention and resources should be allocated to this category of diseases, which are often given less focus than chronic cardiovascular ailments, diabetes, or cancer.^27^ Multiple potential policy solutions exist that could help to make progress in the regions where high YLL rates account for the majority of chronic respiratory disease-related DALYs. First, as respiratory diseases are often difficult to diagnose^28^ in resource-constrained settings because of the widespread lack of important diagnostic equipment (eg, spirometry and radiography equipment), ensuring that such capabilities are part of any primary health-care strategy is crucial for improving time to diagnosis and early intervention.^4^

Second, most risk factors for chronic respiratory diseases are preventable. As we report, in south Asia and in southeast Asia, east Asia, and Oceania, ambient indoor and outdoor air pollution cause a far greater share of chronic respiratory disease-attributable disability than they do in the high-income super-region and the central Europe, eastern European, and central Asia super-region, where greater affluence correlates with higher smoking-attributable disease. Different strategies to minimise exposure to these well known risk factors have been previously described and remain underutilised globally. For example, for tobacco, WHO advocates measures such as banning advertising, creating tobacco-free spaces, inserting health warnings on the packaging of tobacco products, and increased cigarette taxation.^29^ New assessments on the effects of exposure to second-hand smoke,^30^ and of new forms of smoking, such as electronic cigarettes,^31^ on health, particularly among children and adolescents, deserve heightened public awareness.

The general limitations of GBD studies have been reported elsewhere and additionally apply to estimates of chronic respiratory diseases,^10^, ^25^ although there are some limitations specific to chronic respiratory diseases. Notably, distinguishing between the different chronic respiratory disease diagnoses requires clear case definitions, which are often not standardised across countries because of the intrinsic difficulty and resource-intensive nature of accurately diagnosing a chronic respiratory disease. However, initiatives to better define asthma, COPD, and their disease mimics are currently in progress.^32^ For diagnoses such as interstitial lung disease, pulmonary sarcoidosis, and pneumoconiosis, in particular, almost all of our input data for prevalence are derived from hospital inpatient records and insurance claims—a major limitation because claims data can be unreliable and highly dependent on the methods for denominator estimation and bias correction. Moreover, for conditions such as pneumoconiosis, prevalence studies have typically been done in small, non-representative groups (eg, employees in a particular occupation), which are not included in GBD. Taken together, these limitations highlight the need for enhanced data collection efforts through the use of more precise and standardised case definitions to help improve cross-country comparisons.

In addition, with regard to COPD, defining the cutoff value for spirometry with the lower limit of normal as the fifth percentile in a healthy reference population makes the uneasy assumption that COPD prevalence cannot be lower than 5%. Recent evidence has identified near-perfect pattern recognition and diagnosis through spirometric manoeuvres by machine learning algorithms,^33^ which are likely to overcome the poor repeatability of human-based interpretation of spirometry. All included COPD surveys were spirometry-based, and we chose the Global Initiative for Chronic Obstructive Lung Disease post-bronchodilator fixed ratio lower than 0·7 as a reference value; different spirometry-based case definitions showed a strong age pattern, which we tried to capture with regression methods. These adjustments add uncertainty, which would be avoided if estimates were all reported in a standard manner.^10^

Asthma diagnosis relies on self-report of wheeze in the past 12 months and self-report of a physician diagnosis, because no physiological measurement is considered a gold standard. Thus, measurement of asthma prevalence is subject to the limitations of recall bias, access to health services, and the different interpretations of survey questions inherent in self-reported health measurements.

Therefore, in all chronic respiratory diseases, and in particular interstitial lung disease and pulmonary sarcoidosis, definition is highly dependent on medical expertise and equipment, and it is likely that a significant source of geographical variation is the lack of adequate diagnosis in less-developed areas.^27^, ^34^

For many countries without functional vital registration systems, we had to rely on death estimates of all chronic respiratory diseases collectively from verbal autopsy studies, as these studies cannot distinguish between asthma, COPD, or other chronic respiratory diseases. Initiatives to strengthen vital registration systems are key to improving population health measurement. Of note, the residual group of other chronic respiratory diseases includes many heterogeneous conditions, some very prevalent, including rhinitis^3^, ^35^ and sleep apnoea.^36^ These conditions merit greater attention in future GBD cycles. The estimation of any specific chronic respiratory disease would benefit from greater precision, both in the primary data and in the analytical methods, in distinguishing between and within chronic respiratory disease categories that often have many overlapping diagnostic criteria.^37^

Finally, incorporating new evidence and analytical strategies for risk factors associated with chronic respiratory diseases, particularly on all types of smoking—from cigarettes^38^ to new forms of heat-not-burn technologies such as vaping—is needed to accurately capture the changing landscape of risk factors for chronic respiratory ailments.^30^, ^39^ Additionally, the issue of confounding between air pollution and smoking has not been addressed.^40^ Analyses that take these confounders into account would be a future aim of the project. For instance, no attributable risk factor analyses could be done for interstitial lung disease and pulmonary sarcoidosis with the currently available evidence.

Chronic respiratory diseases are important contributors to the burden of NCDs. Although much of the burden is either preventable or treatable with affordable interventions, these diseases have received less attention than other prominent NCDs such as cardiovascular disease, diabetes, and cancer.^41^ Although global age-standardised morbidity and mortality rates for chronic respiratory diseases dropped substantially between 1990 and 2017, the fact that chronic respiratory disease prevalence is increasing in absolute terms in high-income nations despite the availability of robust health-care services suggests that either some risk factors have not been sufficiently addressed or provisioning of resources to address this persistent burden remains inadequate.

Up-to-date population-level estimates on these common respiratory conditions is key to effective policy making with the aim of improving access to care and scaling of robust prevention strategies. We call for greater standardisation in data collection with regard to case definitions and severity distributions of all NCDs in general, and chronic respiratory diseases in particular. More and updated population measurements of chronic respiratory diseases are needed to better quantify the size of the problem and to better monitor progress towards achievement of the 2030 health-related Sustainable Development Goals.^42^

Correspondence to: Dr Joan B Soriano, Hospital Universitario de la Princesa, Universidad Autónoma de Madrid, 28005 Madrid, Spain jbsoriano2@gmail.com
