# Supplementary material for: Prevalence and attributable health burden of chronic respiratory diseases, 1990–2017: a systematic analysis for the Global Burden of Disease Study 2017
Source: Lancet Respir Med. 2020 Jun;8(6):585–96. doi: 10.1016/S2213-2600(20)30105-3 (PMC7284317; doi:10.1016/S2213-2600(20)30105-3)

THE LANCET
Respiratory Medicine

Supplementary appendix 1

This appendix formed part of the original submission and has been peer reviewed. We post it as supplied by the authors.

Supplement to: GBD Chronic Respiratory Disease Collaborators. Prevalence and attributable health burden of chronic respiratory diseases, 1990–2017: a systematic analysis for the Global Burden of Disease Study 2017. *Lancet Respir Med* 2020; **8:** 585–96.

ONLINE APPENDIX 1

Prevalence and Attributable Health Burden of Chronic Respiratory Diseases from 1990–2017: A systematic analysis from the Global Burden of Disease Study 2017

Joan B Soriano,[1,2,3] Parkes Kendrick,[4] Katherine Paulson,[4] Vinay Gupta,[4] Theo Vos,[4] and the GBD Chronic Respiratory Disease Collaborators

[1] Associate Professor of Medicine, Hospital Universitario de la Princesa, Universidad Autónoma de Madrid, Madrid, Spain

[2] Centro de Investigación en Red de Enfermedades Respiratorias (CIBERES), Instituto de Salud Carlos III (ISCIII), Madrid, Spain

[3] Hospital Universitari Son Espases, Universitat de les Illes Balears, Palma, Spain

[4] Institute for Health Metrics Evaluation, University of Washington, Seattle, WA, USA.

Correspondence:

Dr. Joan B Soriano, MD, PhD, FERS, FCCP

Associate Professor of Medicine

Hospital Universitario de la Princesa, UAM

Diego de León 62, 28005-Madrid (SPAIN)

Email: jbsoriano2@gmail.com

Date: 16 May 2020

File name: Online Appendix 1 CRD GBD 2017 paper – 16 May 2020.docx

Table Contents of Online Appendix 1

AUTHOR LIST 5

ACKNOWLEDGEMENTS 14

DECLARATIONS 15

ONLINE METHODS DISEASE INDIVIDUAL WRITE-UPS FOR CHRONIC RESPIRATORY CONDITIONS IN GBD 2017 16

CHRONIC RESPIRATORY DISEASES 16
Input data 16
Modelling strategy 16
CHRONIC OBSTRUCTIVE PULMONARY DISEASE 18
Input data 18
Modelling strategy 18
PNEUMOCONIOSIS DISEASES: SILICOSIS, ASBESTOSIS, COAL WORKER'S PNEUMOCONIOSIS, AND OTHER PNEUMOCONIOSIS 20
Input data 20
Modelling strategy 20
ASTHMA 22
Input data 22
Modelling strategy 22
INTERSTITIAL LUNG DISEASE AND PULMONARY SARCOIDOSIS 24
Input data 24
Modelling strategy 24
OTHER CHRONIC RESPIRATORY DISEASES 26
Input data 26
Modelling strategy 26

CHRONIC OBSTRUCTIVE PULMONARY DISEASE (COPD) 28

FLOWCHART 28
INPUT DATA AND METHODOLOGICAL SUMMARY 28
CASE DEFINITION 28
INPUT DATA 29
MODELLING STRATEGY 30

SEVERITY 31

PNEUMOCONIOSIS 33

COAL WORKER'S PNEUMOCONIOSIS, ASBESTOSIS, SILICOSIS, AND OTHER PNEUMOCONIOSIS 33

FLOWCHART 33
INPUT DATA AND METHODOLOGICAL APPENDIX 33
CASE DEFINITION 33
INPUT DATA 33
SEVERITY SPLIT INPUTS 34
MODELLING STRATEGY 35

ASTHMA 36

FLOWCHART 36
CASE DEFINITION 36

Input data ... 36
Modelling strategy ... 37
Severity split inputs ... 39

INTERSTITIAL LUNG DISEASE AND PULMONARY SARCOIDOSIS (ILD) ... 40

Flowchart ... 40
Case definition ... 40
Input data ... 40
Modelling strategy ... 41

OTHER CHRONIC RESPIRATORY DISEASES ... 43

ONLINE METHODS RISKS FOR INDIVIDUAL WRITE-UPS FOR RESPIRATORY RISK FACTORS IN GBD 2017 ... 44

SMOKING CAPSTONE APPENDIX ... 44

Flowchart ... 44
Current and former smoking prevalence ... 45
Data extraction ... 45
Crosswalk ... 45
Age and sex splitting ... 46
Smoking prevalence modelling ... 46
Exposure among current and former smokers ... 47
Risk-outcome pairs ... 47
Dose-response risk curves ... 47
PAF calculation ... 48

SECONDHAND SMOKE CAPSTONE APPENDIX ... 49

Flowchart ... 49
Exposure ... 49
Case definition ... 49
Input data ... 49
Modelling strategy ... 50
Theoretical minimum-risk exposure level ... 50
Relative risks ... 50

AMBIENT OZONE POLLUTION CAPSTONE APPENDIX ... 52

Flowchart ... 52
Input data and methodological summary ... 52
Exposure ... 52
Theoretical minimum-risk exposure level ... 52
Relative risks ... 53
References ... 53

OCCUPATIONAL RISK FACTORS CAPSTONE APPENDIX ... 54

Exposure definitions ... 54
Input data ... 55
Modelling strategies ... 55
Occupational carcinogens, occupational noise, and occupational particulates ... 56
Occupational ergonomic factors and occupational asthmagens ... 56
Occupational injuries ... 56

Occupational asbestos ... *57*
Theoretical minimum-risk exposure level ... 57
For all occupational risks, with the exception of occupational asbestos, the theoretical minimum-risk exposure level was assumed to be no exposure to that risk ... *57*
Relative risk ... 58
PAFs ... 58
Occupational injuries PAF ... *58*
References ... 58
AMBIENT PARTICULATE MATTER POLLUTION CAPSTONE APPENDIX ... 59
Flowchart ... 59
Input data and modeling strategy ... 60
Exposure ... *60*
Theoretical minimum-risk exposure level ... 65
Relative risks and population attributable fractions ... 66
Integrated exposure response function ... *66*
Relative risk and proportional PAF approach ... *67*
References ... 68
HOUSEHOLD AIR POLLUTION CAPSTONE APPENDIX ... 69
Flowchart ... 69
Input Data & Methodological Summary ... 69
Exposure ... *69*
Theoretical minimum-risk exposure level ... 70
Relative risks ... 70
$PM_{2.5}$ mapping value ... *70*
References ... 71
ONLINE RESULTS APPENDIX - SUPPLEMENTARY TABLES AND FIGURES ... 72
Supplementary Table 1: ... 72
Supplementary Table 2: ... 73
Online Figure 1 ... 78
Online Figure 2a ... 79
Online Figure 2b ... 80
Online Figure 2c ... 81

Author list

Joan B Soriano, Parkes J Kendrick, Katherine R Paulson, Vinay Gupta, Elissa M Abrams, Rufus Adesoji Adedoyin, Tara Ballav Adhikari, Shailesh M Advani, Anurag Agrawal, Elham Ahmadian, Fares Alahdab, Syed Mohamed Aljunid, Khalid A Altirkawi, Nelson Alvis-Guzman, Nahla Hamed Anber, Catalina Liliana Andrei, Mina Anjomshoa, Fereshteh Ansari, Josep M Antó, Jalal Arabloo, Seyyede Masoume Athari, Seyyed Shamsadin Athari, Nefsu Awoke, Alaa Badawi, Joseph Adel Mattar Banoub, Derrick A Bennett, Isabela M Bensenor, Kathleen Sachiko Berfield, Robert S Bernstein, Krittika Bhattacharyya, Ali Bijani, Michael Brauer, Gene Bukhman, Zahid A Butt, Luis Alberto Cámera, Josip Car, Juan J Carrero, Felix Carvalho, Carlos A Castañeda-Orjuela, Jee-Young Jasmine Choi, Devasahayam J Christopher, Aaron J Cohen, Lalit Dandona, Rakhi Dandona, Anh Kim Dang, Ahmad Daryani, Barbora de Courten, Feleke Mekonnen Demeke, Gebre Teklemariam Demoz, Jan-Walter De Neve, Rupak Desai, Samath Dhamminda Dharmaratne, Daniel Diaz, Abdel Douiri, Tim Robert Driscoll, Eyasu Ejeta Duken, Aziz Eftekhari, Hajer Elkout, Aman Yesuf Endries, Ibtihal Fadhil, Andre Faro, Farshad Farzadfar, Eduarda Fernandes, Irina Filip, Florian Fischer, Masoud Foroutan, M.A. Garcia-Gordillo, Abadi Kahsu Gebre, Ketema Bizuwork Gebremedhin, Gebreamlak Gebremedhn Gebremeskel, Kebede Embaye Gezae, Aloke Gopal Ghoshal, Paramjit Singh Gill, Richard F Gillum, Houman Goudarzi, Yuming Guo, Rajeev Gupta, Gessessew Bugssa Hailu, Amir Hasanzadeh, Hamid Yimam Hassen, Simon I Hay, Chi Linh Hoang, Michael K Hole, Nobuyuki Horita, H Dean Hosgood, Mihaela Hostiuc, Mowafa Househ, Olayinka Stephen Ilesanmi, Milena D Ilic, Seyed Sina Naghibi Irvani, Sheikh Mohammed Shariful Islam, Mihajlo Jakovljevic, Amr A Jamal, Ravi Prakash Jha, Jost B Jonas, Zubair Kabir, Amir Kasaeian, Gebremicheal Gebreslassie Kasahun, Getachew Mullu Kassa, Adane Teshome Kefale, Andre Pascal Kengne, Yousef Saleh Khader, Morteza Abdullatif Khafaie, Ejaz Ahmad Khan, Junaid Khan, Jagdish Khubchandani, Young-Eun Kim, Yun Jin Kim, Sezer Kisa, Adnan Kisa, Luke D Knibbs, Hamidreza Komaki, Parvaiz A Koul, Ai Koyanagi, G Anil Kumar, Qing Lan, Savita Lasrado, Paolo Lauriola, Carlo La Vecchia, Tham Thi Le, James Leigh, Miriam Levi, Shanshan Li, Alan D Lopez, Paulo A Lotufo, Fabiana Madotto, Narayan B Mahotra, Marek Majdan, Azeem Majeed, Reza Malekzadeh, Abdullah A Mamun, Navid Manafi, Farzad Manafi, Lorenzo Giovanni Mantovani, Birhanu Geta Meharie, Hagazi Gebre Meles, Gebrekiros Gebremichael Meles, Ritesh G Menezes, Tomislav Mestrovic, Ted R Miller, GK Mini, Erkin M Mirrakhimov, Babak Moazen, Karzan Abdulmuhsin Mohammad, Shafiu Mohammed, Farnam Mohebi, Ali H Mokdad, Mariam Molokhia, Lorenzo Monasta, Masoud Moradi, Ghobad Moradi, Lidia Morawska, Seyyed Meysam Mousavi, Kamarul Imran Musa, Ghulam Mustafa, Mehdi Naderi, Mohsen Naghavi, Gurudatta Naik, Sanjeev Nair, Vinay Nangia, Jobert Richie Nansseu, Javad Nazari, Duduzile Edith Ndwandwe, Ruxandra Irina Negoi, Trang Huyen Nguyen, Cuong Tat Nguyen, Huong Lan Thi Nguyen, Molly R Nixon, Richard Ofori-Asenso, Felix Akpojene Ogbo, Andrew T Olagunju, Tinuke O Olagunju, Eyal Oren, Justin R Ortiz, Mayowa O Owolabi, Mahesh P A, Smita Pakhale, Adrian Pana, Songhomitra Panda-Jonas, Eun-Kee Park, Hai Quang Pham, Maarten J Postma, Hadi Pourjafar, Hossein Poustchi, Amir Radfar, Alireza Rafiei, Fakher Rahim, Mohammad Hifz Ur Rahman, Muhammad Aziz Rahman, Salman Rawaf, David Laith Rawaf, Lal Rawal, Robert C Reiner Jr., Marissa Bettay Reitsma, Leonardo Roever, Luca Ronfani, Elias Merdassa Roro, Gholamreza Roshandel, Kristina E Rudd, Yogesh Damodar Sabde, Siamak Sabour, Basema Saddik, Saeed Safari, Komal Saleem, Abdallah M Samy, Milena M Santric-Milicevic, Bruno Piassi Sao Jose, Benn Sartorius, Maheswar Satpathy, Miloje Savic, Monika

Sawhney, Sadaf G Sepanlou, Masood Ali Shaikh, Aziz Sheikh, Mika Shigematsu, Reza Shirkoohi, Si Si, Soraya Siabani, Virendra Singh, Jasvinder A Singh, Michael Soljak, Ranjani Somayaji, Moslem Soofi, Ireneous N Soyiri, Yonatal Mesfin Tefera, Mohamad-Hani Temsah, Berhe Etsay Tesfay, Jarnail Singh Thakur, Alemayehu Toma Toma, Miguel Tortajada-Girbés, Khanh Bao Tran, Bach Xuan Tran, Lorainne Tudor Car, Irfan Ullah, Marco Vacante, Pascual R Valdez, Job F M van Boven, Tommi Juhani Vasankari, Yousef Veisani, Francesco S Violante, Gregory R Wagner, Ronny Westerman, Charles D A Wolfe, Dawit Zewdu Wondafrash, Adam Belay Wondmieneh, Naohiro Yonemoto, Seok-Jun Yoon, Zoubida Zaidi, Mohammad Zamani, Heather J Zar, Yunquan Zhang, and Theo Vos

Affiliations

Hospital Universitario de La Princesa (Princess University Hospital) (Prof J B Soriano MD), Autonomous University of Madrid, Madrid, Spain; Centro de Investigación Biomédica en Red Enfermedades Respiratorias (CIBERES) (Center for Biomedical Research in Respiratory Diseases Network), Madrid, Spain (Prof J B Soriano MD); Institute for Health Metrics and Evaluation (P J Kendrick BS, K R Paulson BS, V Gupta MD, Prof M Brauer DSc, A J Cohen DSc, Prof L Dandona MD, Prof R Dandona PhD, Prof S D Dharmaratne MD, Prof S I Hay FMedSci, Prof A D Lopez PhD, Prof A H Mokdad PhD, Prof M Naghavi MD, M R Nixon PhD, R C Reiner Jr. PhD, M B Reitsma BS, Prof T Vos PhD), Department of Surgery (K S Berfield MD), Department of Health Metrics Sciences, School of Medicine (Prof R Dandona PhD, Prof S D Dharmaratne MD, Prof S I Hay FMedSci, Prof A D Lopez PhD, Prof A H Mokdad PhD, Prof M Naghavi MD, R C Reiner Jr. PhD, Prof B Sartorius PhD, Prof T Vos PhD), Department of Global Health (J R Ortiz MD), Department of Medicine (R Somayaji MD), University of Washington, Seattle, WA, USA (Prof E Oren PhD); Section of Allergy and Clinical Immunology (E M Abrams MD), Ophthalmology Department (N Manafi MD), University of Manitoba, Winnipeg, MB, Canada; Department of Paediatrics (E M Abrams MD), School of Population and Public Health (Prof M Brauer DSc), University of British Columbia, Vancouver, BC, Canada; Department of Medical Rehabilitation (Prof R A Adedoyin PhD), Obafemi Awolowo University, Ile-Ife, Nigeria; Department of Public Health (T B Adhikari MPH), Aarhus University, Aarhus, Denmark; Nepal Health Frontiers (T B Adhikari MPH), University of Southern Denmark, Kathmandu, Nepal; Social Behavioral Research Branch (S M Advani PhD), National Institute of Health, Bethesda, MD, USA; Department of Oncology (S M Advani PhD), Georgetown University, Washington DC, DC, USA; Institute of Genomics and Integrative Biology (Prof A Agrawal PhD), Council of Scientific & Industrial Research, Delhi, India; Internal Medicine (Prof A Agrawal PhD), Baylor College of Medicine, Houston, TX, USA; Research Center for Chronic Kidney Disease (E Ahmadian PhD), Research Center for Evidence Based Medicine (F Ansari PhD), Tabriz University of Medical Sciences, Tabriz, Iran; Joint Ukraine-Azerbaijan International Research and Education Center of Nanobiotechnology and Functional Nanosystems, Baku, Azerbaijan (E Ahmadian PhD); Mayo Evidence-based Practice Center (F Alahdab MSc), Mayo Clinic Foundation for Medical Education and Research, Rochester, MN, USA; Department of Health Policy and Management (Prof S M Aljunid PhD), Kuwait University, Safat, Kuwait; International Centre for Casemix and Clinical Coding (Prof S M Aljunid PhD), National University of Malaysia, Bandar Tun Razak, Malaysia; Pediatric Intensive Care Unit (K A Altirkawi MD, M Temsah MD), Department of Family and

Community Medicine (A A Jamal MD), King Saud University, Riyadh, Saudi Arabia; Research Group in Health Economics (Prof N Alvis-Guzman PhD), University of Cartagena, Cartagena, Colombia; Research Group in Hospital Management and Health Policies (Prof N Alvis-Guzman PhD), University of the Coast, Barranquilla, Colombia; Faculty of Medicine (N H Anber DrPH), Mansoura University, Mansoura, Egypt (N H Anber DrPH); Cardiology Department (C Andrei PhD), Department of Internal Medicine (M Hostiuc PhD), Department of Anatomy and Embryology (R I Negoi PhD), Carol Davila University of Medicine and Pharmacy, Bucharest, Romania; Social Determinants of Health Research Center (M Anjomshoa PhD), Rafsanjan University of Medical Sciences, Rafsanjan, Iran; Razi Vaccine and Serum Research Institute (F Ansari PhD), Agricultural Research, Education, and Extension Organization (AREEO), Tehran, Iran; Non-Communicable Diseases & Environment Programme (Prof J M Antó MD), Barcelona Institute for Global Health, Barcelona, Spain; Department of Experimental and Health Sciences (Prof J M Antó MD), Pompeu Fabra University, Barcelona, Spain; Health Management and Economics Research Center (J Arabloo PhD), Pars Advanced and Minimally Invasive Medical Manners Research Center (A Kasaeian PhD), Department of Ophthalmology (N Manafi MD), Iran University of Medical Sciences, Tehran, Iran; Department of Biology (S Athari MPH), Department of Pharmacology and Toxicology (A Eftekhari PhD), Department of Microbiology (A Hasanzadeh PhD), Department of Nutrition and Food Sciences (H Pourjafar PhD), Maragheh University of Medical Sciences, Maragheh, Iran; Department of Immunology (S Athari MPH), Zanjan University of Medical Sciences, Zanjan, Iran; Department of Nursing (N Awoke MSc), Wolaita Sodo University, Wolaita Sodo, Ethiopia; Public Health Risk Sciences Division (A Badawi PhD), Public Health Agency of Canada, Toronto, ON, Canada; Department of Nutritional Sciences (A Badawi PhD), Joint Centre for Bioethics (F Manafi MD), University of Toronto, Toronto, ON, Canada; Department of Internal Medicine (J A M Banoub MRCP), University of London, London, UK; Department of General Medicine (J A M Banoub MRCP), Alexandria University, Alexandria, Egypt; Nuffield Department of Population Health (D A Bennett PhD), University of Oxford, Oxford, UK; Department of Internal Medicine (I M Bensenor PhD), University of São Paulo, São Paulo, Brazil; Thoracic Surgery (K S Berfield MD), Department of Veterans Affairs, Seattle, WA, USA; Hubert Department of Global Health (R S Bernstein MD), Emory University, Atlanta, GA, USA; Department of Statistical and Computational Genomics (K Bhattacharyya MSc), National Institute of Biomedical Genomics, Kalyani, India; Department of Statistics (K Bhattacharyya MSc), University of Calcutta, Kolkata, India; Social Determinants of Health Research Center (A Bijani PhD), Student Research Committee (M Zamani MD), Babol University of Medical Sciences, Babol, Iran; Department of Global Health and Social Medicine (G Bukhman MD), Division of General Internal Medicine (Prof A Sheikh MD), Department of Environmental Health (G R Wagner MD), Harvard University, Boston, MA, USA; Partners In Health, Boston, MA, USA (G Bukhman MD); School of Public Health and Health Systems (Z A Butt PhD), University of Waterloo, Waterloo, ON, Canada; Al Shifa School of Public Health (Z A Butt PhD), Al Shifa Trust Eye Hospital, Rawalpindi, Pakistan; Internal Medicine Department (Prof L A Cámera MD), Hospital Italiano de Buenos Aires, Buenos Aires, Argentina; Board of Directors (Prof L A Cámera MD), Argentine Society of Medicine, Buenos Aires, Argentina (Prof P R Valdez M.Ed.); Centre for Population Health Sciences (J Car PhD), Lee Kong Chian School of Medicine (M Soljak PhD, L Tudor Car PhD), Nanyang Technological University, Singapore, Singapore; Department of Primary Care and Public Health (J Car PhD, Prof A Majeed MD, Prof S Rawaf MD, M Soljak PhD), WHO Collaborating Centre for Public Health Education and Training

(D L Rawaf MD), Imperial College London, London, UK; Department of Medical Epidemiology and Biostatistics (Prof J J Carrero PhD), Karolinska Institutet, Stockholm, Sweden; Research Unit on Applied Molecular Biosciences (UCIBIO) (Prof F Carvalho PhD), Associated Laboratory for Green Chemistry (LAQV) (Prof E Fernandes PhD), University of Porto, Porto, Portugal; Colombian National Health Observatory (C A Castañeda-Orjuela MD), National Institute of Health, Bogota, Colombia; Epidemiology and Public Health Evaluation Group (C A Castañeda-Orjuela MD), National University of Colombia, Bogota, Colombia; Biomedical Informatics (J J Choi PhD), Seoul National University Hospital, Seoul, South Korea; Department of Pulmonary Medicine (Prof D J Christopher MD), Christian Medical College and Hospital (CMC), Vellore, India; Health Effects Institute, Boston, MA, USA (A J Cohen DSc); Public Health Foundation of India, Gurugram, India (Prof L Dandona MD, Prof R Dandona PhD, G Kumar PhD); Indian Council of Medical Research, New Delhi, India (Prof L Dandona MD); Institute for Global Health Innovations (A K Dang MD, C T Nguyen MPH, H L T Nguyen MPH, H Q Pham MD), Duy Tan University, Hanoi, Vietnam; Toxoplasmosis Research Center (Prof A Daryani PhD), Department of Immunology (Prof A Rafiei PhD), Molecular and Cell Biology Research Center (Prof A Rafiei PhD), Mazandaran University of Medical Sciences, Sari, Iran; The School of Clinical Sciences (Prof B de Courten PhD), Department of Epidemiology and Preventive Medicine (Prof Y Guo PhD, R Ofori-Asenso PhD), School of Public Health and Preventive Medicine (S Li PhD, S Si PhD), Monash University, Melbourne, VIC, Australia; Department of Medical Laboratory Sciences (F M Demeke MSc), Bahir Dar University, Bahir Dar, Ethiopia; School of Pharmacy (G T Demoz MSc, G G Kasahun MSc), Department of Nursing (G G Gebremeskel MSc), Aksum University, Aksum, Ethiopia; Heidelberg Institute of Global Health (HIGH) (J De Neve MD, B Moazen MSc, S Mohammed PhD), Department of Ophthalmology (S Panda-Jonas MD), Heidelberg University, Heidelberg, Germany; Division of Cardiology (R Desai MBBS), Atlanta Veterans Affairs Medical Center, Decatur, GA, USA; Department of Community Medicine (Prof S D Dharmaratne MD), University of Peradeniya, Peradeniya, Sri Lanka; Center of Complexity Sciences (Prof D Diaz PhD), National Autonomous University of Mexico, Mexico City, Mexico; Faculty of Veterinary Medicine and Zootechnics (Prof D Diaz PhD), Autonomous University of Sinaloa, Culiacan Rosales, Mexico; School of Population Health and Environmental Sciences (A Douiri PhD, Prof C D A Wolfe MD), Faculty of Life Sciences and Medicine (M Molokhia PhD), King's College London, London, UK; Sydney School of Public Health (Prof T R Driscoll PhD), Sydney Medical School (S Islam PhD), Asbestos Diseases Research Institute (J Leigh MD), University of Sydney, Sydney, NSW, Australia; College of Health Sciences (E Duken MSc), Department of Public Health (E M Roro MPH), Wollega University, Nekemte, Ethiopia; Mycobacteriology Research Center (E Duken MSc), Jimma University, Jimma, Ethiopia; Department of Pharmacology and Toxicology (A Eftekhari PhD), The John Paul II Catholic University of Lublin, Lublin, Poland; Department of Community Medicine (H Elkout PhD), Tripoli University, Tripoli, Libya; Health Information (H Elkout PhD), World Health Organization (WHO), Tripoli, Libya; Public Health Department (A Y Endries MPH), St. Paul's Hospital Millennium Medical College, Addis Ababa, Ethiopia; Division of Non-Communicable Diseases (I Fadhil PhD), Ministry of Public Health and Population, Dubai, United Arab Emirates; Department of Psychology (Prof A Faro PhD), Federal University of Sergipe, São Cristóvão, Brazil; Non-communicable Diseases Research Center (Prof F Farzadfar DSc, F Mohebi MD), Department of Microbiology (A Hasanzadeh PhD), Hematology, Oncology and Stem Cell Transplantation Research Center (A Kasaeian PhD), Digestive Diseases Research Institute (Prof R Malekzadeh MD, H Poustchi PhD, S G Sepanlou MD), National Institute of

Health Research (NIHR) (F Mohebi MD), Department of Health Policy, Management, and Economics (S Mousavi PhD), Metabolomics and Genomics Research Center (F Rahim PhD), Cancer Research Institute (R Shirkoohi PhD), Cancer Biology Research Center (R Shirkoohi PhD), Tehran University of Medical Sciences, Tehran, Iran; Psychiatry Department (I Filip MD), Kaiser Permanente, Fontana, CA, USA; School of Health Sciences (I Filip MD), A.T. Still University, Mesa, AZ, USA; Institute of Gerontological Health Services and Nursing Research (F Fischer PhD), Ravensburg-Weingarten University of Applied Sciences, Weingarten, Germany; Department of Medical Parasitology (M Foroutan PhD), Abadan Faculty of Medical Sciences, Abadan, Iran; Faculty of Business and Management (M Garcia-Gordillo PhD), Universidad Autonóma de Chile (Autonomous University of Chile), Talca, Chile; School of Pharmacy (A K Gebre MSc), Department of Nursing (G G Gebremeskel MSc), Department of Biostatistics (K Gezae MSc), Department of Medical Parasitology and Entomology (G B Hailu MSc), School of Public Health (G G Meles MPH), Department of Pharmacology and Toxicology (D Z Wondafrash MSc), Mekelle University, Mekelle, Ethiopia (H G Meles MPH); School of Medical and Health sciences (A K Gebre MSc), Edith Cowan University, Perth, WA, Australia; Department of Nursing and Midwifery (K B Gebremedhin MSc), School of Public Health (E M Roro MPH), Department of Pharmacology (D Z Wondafrash MSc), School of Nursing and Midwifery (A B Wondmieneh MSc), Addis Ababa University, Addis Ababa, Ethiopia; Respiratory Medicine (Prof A G Ghoshal MD), National Allergy Asthma Bronchitis Institute, Kolkata, India; Department of Respiratory Medicine (Prof A G Ghoshal MD), Fortis Hospital, Kolkata, India; Medical School (Prof P S Gill DM), University of Warwick, Coventry, UK; Division of General Internal Medicine (R F Gillum MD), Department of Community and Family Medicine (R F Gillum MD), Howard University, Washington, DC, USA; Department of Respiratory Medicine (H Goudarzi PhD), Center for Environmental and Health Sciences (H Goudarzi PhD), Hokkaido University, Sapporo, Japan; Department of Epidemiology (Prof Y Guo PhD), Binzhou Medical University, Yantai City, China; Department of Preventive Cardiology (Prof R Gupta MD), Eternal Heart Care Centre & Research Institute, Jaipur, India; Department of Medicine (Prof R Gupta MD), Mahatma Gandhi University Medical Sciences, Jaipur, India; Department of Primary and Interdisciplinary Care (H Y Hassen MPH), University Hospital Antwerp, Antwerp, Belgium; Department of Public Health (H Y Hassen MPH), Mizan-Tepi University, Mizan Teferi, Ethiopia; Center of Excellence in Behavioral Medicine (C L Hoang B.Med.Sc., T H Nguyen B.Med.Sc.), Nguyen Tat Thanh University, Ho Chi Minh City, Vietnam; Department of Pediatrics (M K Hole MD), University of Texas Austin, Austin, TX, USA; Department of Pulmonology (N Horita PhD), Yokohama City University, Kanazawa-ku, Yokohama, Japan; National Human Genome Research Institute (NHGRI) (N Horita PhD), National Institutes of Health, Bethesda, MD, USA; Department of Epidemiology and Population Health (H Hosgood PhD), Albert Einstein College of Medicine, Bronx, NY, USA; College of Science and Engineering (Prof M Househ PhD), Hamad Bin Khalifa University, Doha, Qatar; Department of Community Medicine (O S Ilesanmi PhD), Department of Medicine (Prof M O Owolabi DrM), University of Ibadan, Ibadan, Nigeria; Department of Community Medicine (O S Ilesanmi PhD), University College Hospital, Ibadan, Nigeria; Department of Epidemiology (Prof M D Ilic PhD), Department of Global Health, Economics and Policy (Prof M Jakovljevic PhD), University of Kragujevac, Kragujevac, Serbia; Research Institute for Endocrine Sciences (S N Irvani MD), Department of Epidemiology (S Sabour PhD), Department of Emergency Medicine (S Safari MD), Shahid Beheshti University of Medical Sciences, Tehran, Iran; Institute for Physical Activity and Nutrition (S Islam PhD), Deakin University, Burwood, VIC, Australia; N. A.

Semashko Department of Public Health and Healthcare (Prof M Jakovljevic PhD), I.M. Sechenov First Moscow State Medical University, Moscow, Russia; Department of Community Medicine (R P Jha MSc), Baba Saheb Ambedkar Medical College & Hospital, Delhi, India; Department of Community Medicine (R P Jha MSc), Banaras Hindu University, Varanasi, India; Department of Ophthalmology (Prof J B Jonas MD), Heidelberg University, Mannheim, Germany; Beijing Institute of Ophthalmology (Prof J B Jonas MD), Beijing Tongren Hospital, Beijing, China; School of Public Health (Z Kabir PhD), University College Cork, Cork, Ireland; College of Health Sciences (G M Kassa MSc), Debre Markos University, Debre Markos, Ethiopia; Department of Pharmacy (A T Kefale MSc), Debre Berhan University, Debre Berhan, Ethiopia; School of Pharmacy (A T Kefale MSc), University of Tasmania, Hobart, TAS, Australia; Non-Communicable Diseases Research Unit (Prof A P Kengne PhD), Unit on Child & Adolescent Health (Prof H J Zar PhD), Medical Research Council South Africa, Cape Town, South Africa; Department of Medicine (Prof A P Kengne PhD), Department of Paediatrics & Child Health (Prof H J Zar PhD), University of Cape Town, Cape Town, South Africa; Department of Public Health (Prof Y S Khader PhD), Jordan University of Science and Technology, Irbid, Jordan; Social Determinants of Health Research Center (M A Khafaie PhD), Thalassemia and Hemoglobinopathy Research Center (F Rahim PhD), Ahvaz Jundishapur University of Medical Sciences, Ahvaz, Iran; Department of Epidemiology and Biostatistics (E A Khan MPH), Health Services Academy, Islamabad, Pakistan; Department of Population Studies (J Khan M.Phil.), International Institute for Population Sciences, Mumbai, India; Department of Nutrition and Health Science (Prof J Khubchandani PhD), Ball State University, Muncie, IN, USA; Department of Preventive Medicine (Y Kim PhD, Prof S Yoon PhD), Korea University, Seoul, South Korea; School of Traditional Chinese Medicine (Y Kim PhD), Xiamen University Malaysia, Sepang, Malaysia; Department of Nursing and Health Promotion (S Kisa PhD), Oslo Metropolitan University, Oslo, Norway; School of Health Sciences (Prof A Kisa PhD), Kristiania University College, Oslo, Norway; Global Community Health and Behavioral Sciences (Prof A Kisa PhD), Tulane University, New Orleans, LA, USA; School of Public Health (L D Knibbs PhD), The University of Queensland, Herston, QLD, Australia; Neurophysiology Research Center (H Komaki MD), Hamadan University of Medical Sciences, Hamadan, Iran; Brain Engineering Research Center (H Komaki MD), Institute for Research in Fundamental Sciences, Tehran, Iran; Department of Internal and Pulmonary Medicine (Prof P A Koul MD), Sheri Kashmir Institute of Medical Sciences, Srinagar, India; CIBERSAM (A Koyanagi MD), San Juan de Dios Sanitary Park, Sant Boi de Llobregat, Spain; Catalan Institution for Research and Advanced Studies (ICREA), Barcelona, Spain (A Koyanagi MD); Division of Cancer Epidemiology and Genetics (Q Lan PhD), National Cancer Institute, Rockville, MD, USA; Department of Otorhinolaryngology (S Lasrado MS), Father Muller Medical College, Mangalore, India; Institute of Clinical Physiology (P Lauriola MD), National Research Council, Pisa, Italy; Department of Clinical Sciences and Community Health (Prof C La Vecchia MD), University of Milan, Milan, Italy; Department of Pharmaceutical Health Services Research (T T Le MPH), Center for Vaccine Development (J R Ortiz MD), University of Maryland, Baltimore, MD, USA; Department of Prevention (M Levi PhD), USL Tuscany Center, Firenze, Italy; Department of Health Sciences (M Levi PhD), University of Florence, Florence, Italy; Melbourne School of Population and Global Health (Prof A D Lopez PhD), University of Melbourne, Melbourne, VIC, Australia; Department of Medicine (Prof P A Lotufo DrPH), University of São Paulo, Sao Paulo, Brazil; Value-Based Healthcare Unit (F Madotto PhD, Prof L G Mantovani DSc), IRCCS MultiMedica, Sesto San Giovanni, Italy; Department of Clinical physiology (N B Mahotra MD),

Tribhuvan University, Kathmandu, Nepal; Department of Public Health (M Majdan PhD), Trnava University, Trnava, Slovakia; Non-communicable Disease Research Center (Prof R Malekzadeh MD, S G Sepanlou MD), Shiraz University of Medical Sciences, Shiraz, Iran; Institute for Social Science Research (A A Mamun PhD), The University of Queensland, Indooroopilly, QLD, Australia; School of Medicine and Surgery (Prof L G Mantovani DSc), University of Milan Bicocca, Monza, Italy; Department of Pharmacy (B Meharie MSc), Department of Environmental Health (Y M Tefera MSc), Department of Nursing (A B Wondmieneh MSc), Wollo University, Dessie, Ethiopia; Forensic Medicine Division (Prof R G Menezes MD), Imam Abdulrahman Bin Faisal University, Dammam, Saudi Arabia; Clinical Microbiology and Parasitology Unit (T Mestrovic PhD), Zora Profozic Polyclinic, Zagreb, Croatia; University Centre Varazdin (T Mestrovic PhD), University North, Varazdin, Croatia; Pacific Institute for Research & Evaluation, Calverton, MD, USA (T R Miller PhD); School of Public Health (T R Miller PhD), Curtin University, Perth, WA, Australia; Global Institute of Public Health (Prof G Mini PhD), Ananthapuri Hospitals and Research Institute, Trivandrum, India; Women's Social and Health Studies Foundation, Trivandrum, India (Prof G Mini PhD); Internal Medicine Programme (Prof E M Mirrakhimov PhD), Kyrgyz State Medical Academy, Bishkek, Kyrgyzstan; Department of Atherosclerosis and Coronary Heart Disease (Prof E M Mirrakhimov PhD), National Center of Cardiology and Internal Disease, Bishkek, Kyrgyzstan; Institute of Addiction Research (ISFF) (B Moazen MSc), Frankfurt University of Applied Sciences, Frankfurt, Germany; Department of Biology (K A Mohammad PhD), Salahaddin University-Erbil, Erbil, Iraq; Health Systems and Policy Research Unit (S Mohammed PhD), Ahmadu Bello University, Zaria, Nigeria; Clinical Epidemiology and Public Health Research Unit (L Monasta DSc, L Ronfani PhD), Burlo Garofolo Institute for Maternal and Child Health, Trieste, Italy; Research Center for Environmental Determinants of Health (M Moradi PhD), Clinical Research Development Center (M Naderi PhD), Department of Health Education and Health Promotion (S Siabani PhD), Social Development and Health Promotion Research Center (M Soofi PhD), Kermanshah University of Medical Sciences, Kermanshah, Iran; Social Determinants of Health Research Center (G Moradi PhD), Department of Epidemiology and Biostatistics (G Moradi PhD), Kurdistan University of Medical Sciences, Sanandaj, Iran; International Laboratory for Air Quality and Health (Prof L Morawska PhD), Queensland University of Technology, Brisbane, QLD, Australia; School of Medical Sciences (K Musa PhD), Science University of Malaysia, Kubang Kerian, Malaysia; Department of Pediatric Medicine (Prof G Mustafa MD), The Children's Hospital & The Institute of Child Health, Multan, Pakistan; Department of Pediatrics & Pediatric Pulmonology (Prof G Mustafa MD), Institute of M & Child Care, Multan, Pakistan; Comprehensive Cancer Center (G Naik MPH), School of Medicine (Prof J A Singh MD), University of Alabama at Birmingham, Birmingham, AL, USA; Department of Pulmonary Medicine (S Nair MD), Government Medical College Trivandrum, Trivandrum, India; Health Action by People, Trivandrum, India (S Nair MD); Suraj Eye Institute, Nagpur, India (V Nangia MD); Department for the Control of Disease, Epidemics, and Pandemics (J Nansseu MD), Ministry of Public Health, Yaoundé, Cameroon; Department of Public Heath (J Nansseu MD), University of Yaoundé I, Yaoundé, Cameroon; Department of Pediatrics (J Nazari MD), Arak University of Medical Sciences, Arak, Iran; Cochrane South Africa (D E Ndwandwe PhD), South African Medical Research Council, Cape Town, South Africa; Cardio-Aid, Bucharest, Romania (R I Negoi PhD); Department of Pharmacy (R Ofori-Asenso PhD), University of Copenhagen, Copenhagen, Denmark; Translational Health Research Institute (F A Ogbo PhD), Western Sydney University, Sydney, NSW, Australia; Department of Psychiatry and Behavioural

Neurosciences (A T Olagunju MD), Department of Pathology and Molecular Medicine (T O Olagunju MD), McMaster University, Hamilton, ON, Canada; Department of Psychiatry (A T Olagunju MD), University of Lagos, Lagos, Nigeria; Graduate School of Public Health (Prof E Oren PhD), San Diego State University, San Diego, CA, USA; Department of Medicine (Prof M O Owolabi DrM), University College Hospital, Ibadan, Ibadan, Nigeria; Department of Respiratory Medicine (Prof M P A DNB), Jagadguru Sri Shivarathreeswara Academy of Health Education and Research, Mysore, India; Department of Medicine (S Pakhale MD), Ottawa Hospital Research Institute, Ottawa, ON, Canada; Department of Statistics and Econometrics (A Pana MD), Bucharest University of Economic Studies, Bucharest, Romania; Department of Health Metrics (A Pana MD), Center for Health Outcomes & Evaluation, Bucharest, Romania; Department of Medical Humanities and Social Medicine (Prof E Park PhD), Kosin University, Busan, South Korea; University Medical Center Groningen (Prof M J Postma PhD, J F M van Boven PhD), School of Economics and Business (Prof M J Postma PhD), University of Groningen, Groningen, Netherlands; Dietary Supplements and Probiotic Research Center (H Pourjafar PhD), Alborz University of Medical Sciences, Karaj, Iran; College of Medicine (A Radfar MD), University of Central Florida, Orlando, FL, USA; Department of Community Medicine (M Rahman PhD), Maharishi Markandeshwar Institute of Medical Sciences & Research, Ambala, India; School of Nursing and Healthcare Professions (M Rahman PhD), Federation University Australia, Berwick, VIC, Australia; School of Nursing and Midwifery (M Rahman PhD), La Trobe University, Melbourne, Victoria, Australia; Academic Public Health England (Prof S Rawaf MD), Public Health England, London, UK; University College London Hospitals, London, UK (D L Rawaf MD); School of Health, Medical and Applied Sciences (L Rawal PhD), CQ University, Sydney, NSW, Australia; Department of Clinical Research (L Roever PhD), Federal University of Uberlândia, Uberlândia, Brazil; Golestan Research Center of Gastroenterology and Hepatology (GRCGH) (G Roshandel PhD), Golestan University of Medical Sciences, Gorgan, Iran; Department of Critical Care Medicine (K E Rudd MD), University of Pittsburgh, Pittsburgh, PA, USA; Environmental Epidemiology and Public Health (Y D Sabde MD), National Institute for Research in Environmental Health, Bhopal, India; Department of Family and Community Medicine (B Saddik PhD), University of Sharjah, Sharjah, United Arab Emirates; Medical and Human Science Department (K Saleem MSc), University of Manchester, Manchester, UK; Division of Medicine (K Saleem MSc), University College London, London, UK; Department of Entomology (A M Samy PhD), Ain Shams University, Cairo, Egypt; Faculty of Medicine (Prof M M Santric-Milicevic PhD), School of Public Health and Health Management (Prof M M Santric-Milicevic PhD), University of Belgrade, Belgrade, Serbia; Department of Infectious Diseases and Tropical Medicine (B P Sao Jose PhD), Federal University of Minas Gerais, Belo Horizonte, Brazil; Faculty of Infectious and Tropical Diseases (Prof B Sartorius PhD), London School of Hygiene & Tropical Medicine, London, UK; UGC Centre of Advanced Study in Psychology (M Satpathy PhD), Utkal University, Bhubaneswar, India; Udyam-Global Association for Sustainable Development, Bhubaneswar, India (M Satpathy PhD); GSK Biologicals, Wavre, Belgium (M Savic PhD); Department of Public Health Sciences (M Sawhney PhD), University of North Carolina at Charlotte, Charlotte, NC, USA; Independent Consultant, Karachi, Pakistan (M A Shaikh MD); Centre for Medical Informatics (Prof A Sheikh MD), University of Edinburgh, Edinburgh, UK; National Institute of Infectious Diseases, Tokyo, Japan (M Shigematsu PhD); School of Health (S Siabani PhD), University of Technology Sydney, Sydney, NSW, Australia; Department of Pulmonary Medicine (Prof V Singh MD), Asthma Bhawan, Jaipur, India; Medicine Service (Prof J A Singh MD), US

Department of Veterans Affairs (VA), Birmingham, AL, USA; Department of Medicine (R Somayaji MD), University of Calgary, Calgary, AB, Canada; Hull York Medical School (I N Soyiri PhD), University of Hull, Hull City, UK; School of Public Health (Y M Tefera MSc), University of Adelaide, Adelaide, SA, Australia; Department of Public Health (B E Tesfay MPH), Adigrat University, Adigrat, Ethiopia; School of Public Health (Prof J S Thakur MD), Post Graduate Institute of Medical Education and Research, Chandigarh, India; Department of Pharmacy (A T Toma PhD), Hawassa University, Hawassa, Ethiopia; Pediatric Department (Prof M Tortajada-Girbés PhD), University Hospital Doctor Peset, Valencia, Spain; Department of Pediatrics, Obstetrics and Gynecology (Prof M Tortajada-Girbés PhD), University of Valencia, Valencia, Spain; Molecular Medicine and Pathology (K B Tran MD), University of Auckland, Auckland, New Zealand; Clinical Hematology and Toxicology (K B Tran MD), Maurice Wilkins Centre, Auckland, New Zealand; Department of Health Economics (B X Tran PhD), Hanoi Medical University, Hanoi, Vietnam; Department of Allied Health Sciences (I Ullah PhD), Iqra National University, Peshawar, Pakistan; Department of General Surgery and Medical-Surgical Specialties (M Vacante PhD), University of Catania, Catania, Italy; Velez Sarsfield Hospital, Buenos Aires, Argentina (Prof P R Valdez M.Ed.); UKK Institute, Tampere, Finland (Prof T J Vasankari MD); Psychosocial Injuries Research Center (Y Veisani PhD), Ilam University of Medical Sciences, Ilam, Iran; Department of Medical and Surgical Sciences (Prof F S Violante MD), University of Bologna, Bologna, Italy; Occupational Health Unit (Prof F S Violante MD), Sant'Orsola Malpighi Hospital, Bologna, Italy; Competence Center of Mortality-Follow-Up of the German National Cohort (R Westerman DSc), Federal Institute for Population Research, Wiesbaden, Germany; NIHR Biomedical Research Centre (Prof C D A Wolfe MD), Guy's and St.Thomas' Hospital and Kings College London, London, UK; Department of Neuropsychopharmaology (N Yonemoto MPH), National Center of Neurology and Psychiatry, Kodaira, Japan; Department of Public Health (N Yonemoto MPH), Juntendo University, Tokyo, Japan; Department of Medicine (Prof Z Zaidi PhD), University Ferhat Abbas of Setif, Sétif, Algeria; School of Public Health (Y Zhang PhD), Hubei Province Key Laboratory of Occupational Hazard Identification and Control (Y Zhang PhD), Wuhan University of Science and Technology, Wuhan, China.

Acknowledgements

Syed Aljunid would like to acknowledge the Department of Health Policy and Management, Faculty of Public Health, Kuwait University and International Centre for Casemix and Clinical Coding, Faculty of Medicine, National University of Malaysia for the approval and support to participate in this research project. Derrick Bennett acknowledges support by the NIHR Oxford Biomedical Research Centre. The views expressed are those of the authors and not necessarily those of the NHS, the NIHR or the Department of Health and Social Care. Juan J Carrero acknowledges support from the Swedish Research Council (2019-01059). Felix Carvalho acknowledges support from UID/MULTI/04378/2019 and UID/QUI/50006/2019 with funding from FCT/MCTES through national funds. Aaron Cohen was supported by Health Effects Institute, Boston MA USA. Jan-Walter De Neve was supported by the Alexander von Humboldt Foundation. Abdel Douiri acknowledges financial support from the National Institute for Health Research (NIHR) Biomedical Research and from the NIHR Collaboration for Leadership in Applied Health Research and Care South London at King's College Hospital NHS Foundation Trust. Sheikh Mohammed Shariful Islam acknowledges funding by the National Heart Foundation of Australia and Deakin University. Mihajlo Jakovljevic acknowledges co-funding through the Grant OI175014 of the Ministry of Education Science and Technological Development for the Serbian contributions to this GBD study. Mariam Molokhia acknowledges support from the National Institute for Health Research Biomedical Research Center at Guy’s and St Thomas’ National Health Service Foundation Trust and King’s College London. Seyed Sina Naghibi Irvani would like to thank the Clinical Research Development Unit (CRDU) of Loghman Hakim Hospital, Shahid Beheshti University of Medical Sciences, Tehran, Iran for their support. Adrian Pana is partially supported by a grant of the Romanian National Authority for Scientific Research and Innovation, CNDS-UEFISCDI, project number PN-III-P4-ID-PCCF-2016-0084. Abdallah M. Samy was supported by a fellowship from the Egyptian Fulbright Mission Program. Milena Santric Milicevic acknowledges the support of the Ministry of Education, Science and Technological Development of the Republic of Serbia (Contract No. 175087). Aziz Sheikh acknowledges Health Data Research UK. Job FM van Boven was supported by the University of Groningen, University Medical Center Groningen, Groningen Research Institute for Asthma and COPD (GRIAC).

Declarations

Dr.Islam reports grants from National Heart Foundation of Australia during the conduct of the study, and grants from Deakin University, outside the submitted work. Dr. Postma reports grants and personal fees from MSD, GSK, Pfizer, Boehringer Ingelheim, Novavax, BMS, Astra Zeneca, Sanofi, IQVIA, and Seqirus, personal fees from Quintiles, Novartis, and Pharmerit, grants from Bayer, BioMerieux, WHO, EU, FIND, Antilope, DIKTI, LPDP, Budi, stocks in Ingress Health and PAG Ltd, and acting as advisor to Asc Academics, all outside the submitted work. Dr. Savic reports employment by GSK Biologicals, Wavre, Belgium, and holding restricted shares in the company. Dr. Singh reports personal fees from Crealta/Horizon, Medisys, Fidia, UBM LLC, Trio health, Medscape, WebMD, Clinical Care options, Clearview healthcare partners, Putnam associates, Spherix, Practice Point communications, the National Institutes of Health and the American College of Rheumatology, and Simply Speaking, stocks in Amarin pharmaceuticals and Viking pharmaceuticals, non-financial support from FDA Arthritis Advisory Committee, Veterans Affairs Rheumatology Field Advisory Committee, UAB Cochrane Musculoskeletal Group Satellite Center on Network Meta-analysis, and the Steering committee of OMERACT, an international organization that develops measures for clinical trials and receives arm’s length funding from 12 pharmaceutical companies, all outside the submitted work.

Online Methods Disease individual write-ups for chronic respiratory conditions in GBD 2017

Chronic Respiratory Diseases

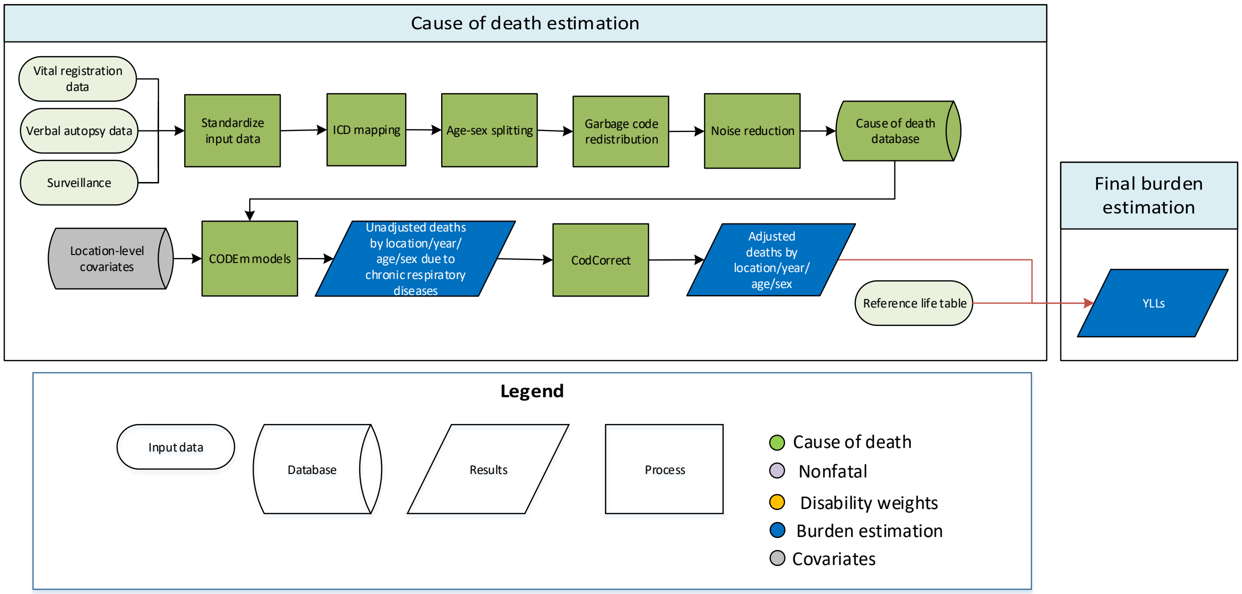

Input data

Sources used to estimate chronic respiratory disease mortality included vital registration, verbal autopsy, and surveillance data from China. Our outlier criteria excluded data points that (1) were implausibly high or low, (2) substantially conflicted with established age or temporal patterns, or (3) significantly conflicted with other data sources conducted from the same locations or locations with similar characteristics (ie, Socio-demographic Index).

Modelling strategy

The standard CODEm modelling approach was applied to estimate deaths due to chronic respiratory diseases. Chronic respiratory diseases served as the parent cause to chronic obstructive pulmonary disease, pneumoconiosis (including silicosis, asbestosis, coal worker's pneumoconiosis, other pneumoconiosis), asthma, interstitial lung disease and pulmonary sarcoidosis, and other chronic respiratory diseases. Functionally, this means the death estimates for chronic respiratory diseases serve as a "parent" envelope into which the "child" causes are squeezed by the CodCorrect algorithm. This approach allows us to use a broader range of data – specifically verbal autopsy data – which cannot be accurately mapped to specific respiratory diseases.

Separate models were conducted for male and female mortality, and the age range for both models was 1 to 95+ years. The same covariates from GBD 2016 were used.

Level	Covariate	Direction

1	log-transformed SEV scalar: chronic respiratory diseases	+
	cumulative cigarettes (10 years)	+
	cumulative cigarettes (5 years)	+
	healthcare quality and access index	-
2	smoking prevalence	+
	indoor air pollution (all cooking fuels)	+
	outdoor air pollution ($PM_{2.5}$)	+
	population above 1500m elevation (proportion)	+
3	log LDI (I$ per capita)	-
	education (years per capita)	-
	Socio-demographic Index	-
	population between 500 and 1,500m elevation (proportion)	+
	population density over 1,000 people/kilometer^2 (proportion)	+

Chronic Obstructive Pulmonary Disease

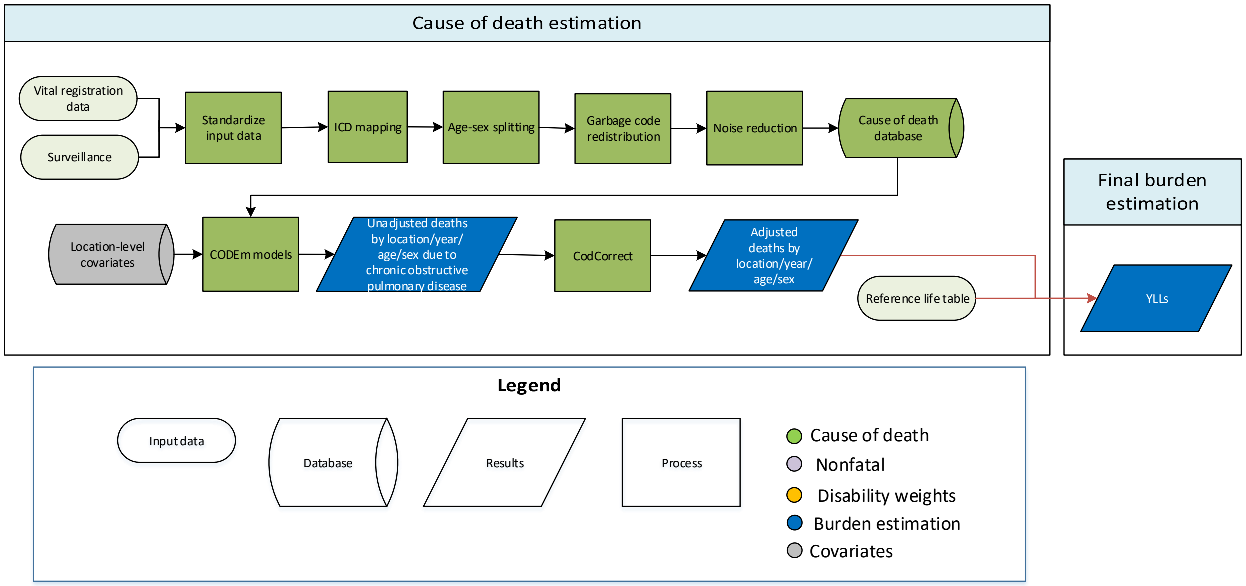

Input data

Data used to estimate chronic obstructive pulmonary disease (COPD) mortality included vital registration and surveillance data from the cause of death (COD) database. Our outlier criteria excluded data points that (1) were implausibly high or low, (2) substantially conflicted with established age or temporal patterns, or (3) significantly conflicted with other data sources conducted from the same locations or locations with similar characteristics (ie, Socio-demographic Index).

Modelling strategy

The standard CODEm modelling approach was applied to estimate deaths due to COPD. Separate models were conducted for male and female mortality, and the age range for both models was 1-95+ years. The mortality estimates from the COPD models were ultimately fit into the chronic respiratory diseases envelope.

The same covariates from GBD 2016 were used, but outdoor air pollution was moved to level 1.

Level	Covariate	Direction
1	log-transformed SEV scalar: COPD	+
	cumulative cigarettes (10 years)	+
	cumulative cigarettes (5 years)	+
	elevation over 1,500m (proportion)	+
	outdoor air pollution ($PM_{2.5}$)	+
2	smoking prevalence	+

	indoor air pollution (all cooking fuels)	+
	healthcare access and quality index	-
3	Socio-demographic Index	-
	log LDI (I$ per capita)	-
	education (years per capita)	-

Pneumoconiosis Diseases: Silicosis, Asbestosis, Coal Worker's Pneumoconiosis, and Other Pneumoconiosis

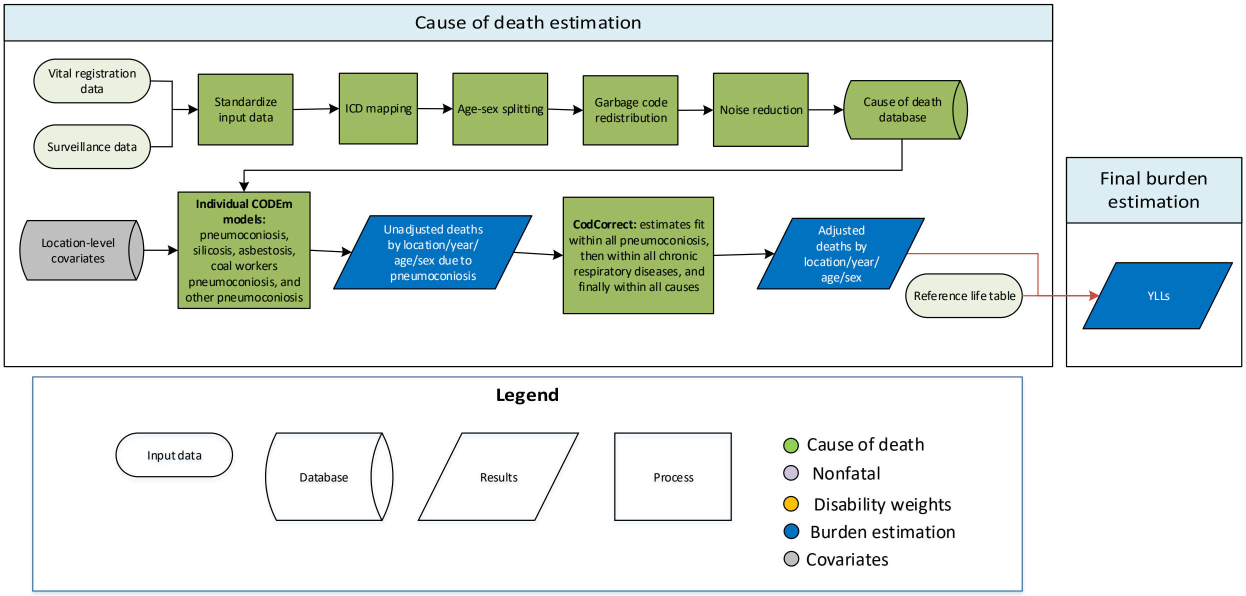

Input data

Data used to estimate pneumoconiosis diseases mortality included vital registration and China mortality surveillance data from the cause of death (COD) database. Our outlier criteria excluded data points that (1) were implausibly high or low, (2) substantially conflicted with established age or temporal patterns, or (3) significantly conflicted with other data sources conducted from the same locations or locations with similar characteristics (ie, socio-demographic index).

Modelling strategy

The standard CODEm modelling approach was applied to estimate deaths due to pneumoconiosis diseases. Separate models were conducted for male and female mortality, and the age range for both models was 15–95+ years. The mortality estimates from pneumoconiosis disease models were ultimately fit into the chronic respiratory envelope, which is the parent cause for pneumoconiosis disease. The pneumoconiosis model serves as an envelope for silicosis, asbestosis, coal worker's pneumoconiosis, and other pneumoconiosis. In CoDCorrect, estimates are first fit within all pneumoconiosis, then within all chronic respiratory disease, before being fit to the all-cause mortality envelope.

For the most part, the same covariates from GBD 2016 were used. The log-transformed SEV scalars were dropped, however, because the associated risk factors for GBD are occupational silica, asbestos, and particulate exposure, which each have a population attributable fraction (PAF) of 1 for pneumoconiosis. When PAF is equal to one, SEV=1/(1-PAF) is undefined. Subnational adjustments were also made to the coal, asbestos, and gold covariates.

The following table indicates covariates used in the pneumoconiosis models, their level, and direction:

Level	Covariate	Direction
1	asbestos consumption per capita*	+
	coal production per capita*	+
	gold production per capita*	+
2	smoking prevalence	+
	indoor air pollution (all cooking fuels)	+
	cumulative cigarettes (5 years)	+
	elevation over 1,500m (proportion)	+
	elevation 500 to 1,500m (proportion)	+
	healthcare access and quality index	-
3	log LDI (I$ per capita)	-
	education (years per capita)	-
	Socio-demographic Index	-

* asbestos, coal, and gold covariates are each only used in a subset of the pneumoconiosis models, as follows: all three are included in the parent all pneumoconiosis model, asbestos consumption is included in the asbestosis model, coal production is included in the coal worker's pneumoconiosis model, and gold production is included in the silicosis model.

Asthma

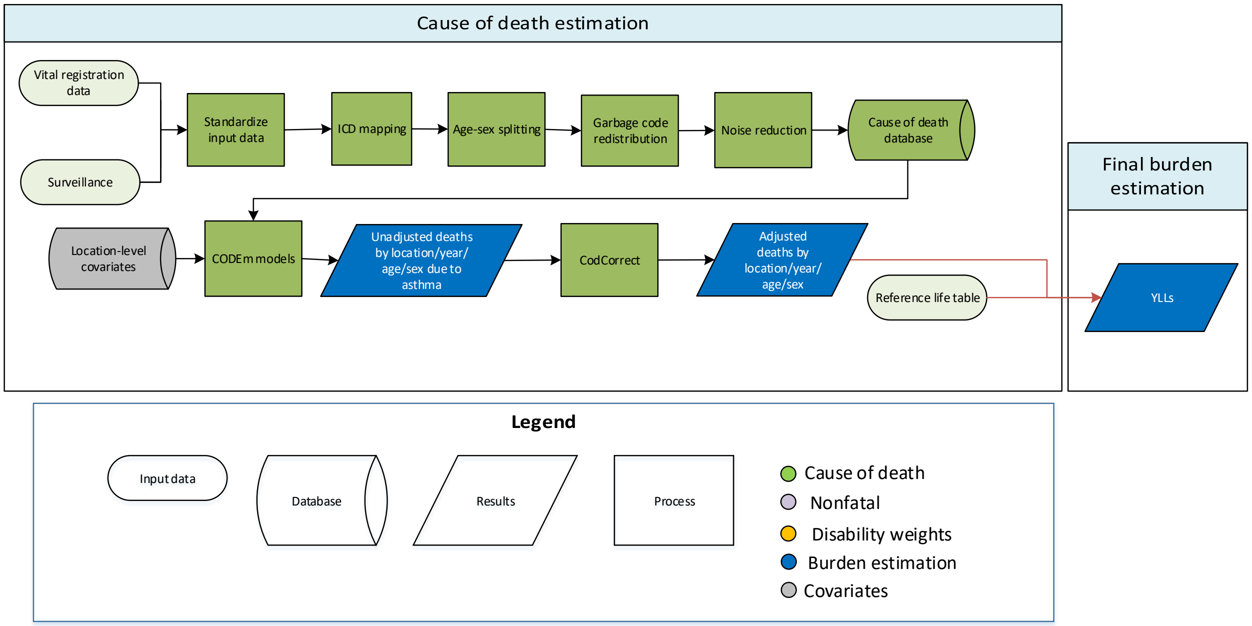

Input data

Data used to estimate asthma mortality included vital registration and surveillance data from the cause of death (COD) database. Verbal autopsy data were not included and were instead mapped to the parent model (chronic respiratory diseases). Our outlier criteria excluded data points that (1) were implausibly high or low relative to global or regional patterns, (2) substantially conflicted with established age or temporal patterns, or (3) significantly conflicted with other data sources conducted from the same locations or locations with similar characteristics (ie, Socio-demographic Index).

Modelling strategy

The standard CODEm modelling approach was applied to estimate deaths due to asthma. Separate models were conducted for male and female mortality, and the age range for both models was 1–95+ years. The mortality estimates from the asthma models were ultimately fit into the chronic respiratory diseases envelope.

The same covariates from GBD 2016 were used.

Level	Covariate	Direction
1	log-transformed SEV scalar: asthma	+
	cumulative cigarettes (10 years)	+
	cumulative cigarettes (5 years)	+
	healthcare access and quality index	-

2	smoking prevalence	+
	indoor air pollution (all cooking fuels)	+
	outdoor air pollution ($PM_{2.5}$)	+
3	log LDI (I$ per capita)	-
	education (years per capita)	-
	Socio-demographic Index	-

Interstitial Lung Disease and Pulmonary Sarcoidosis

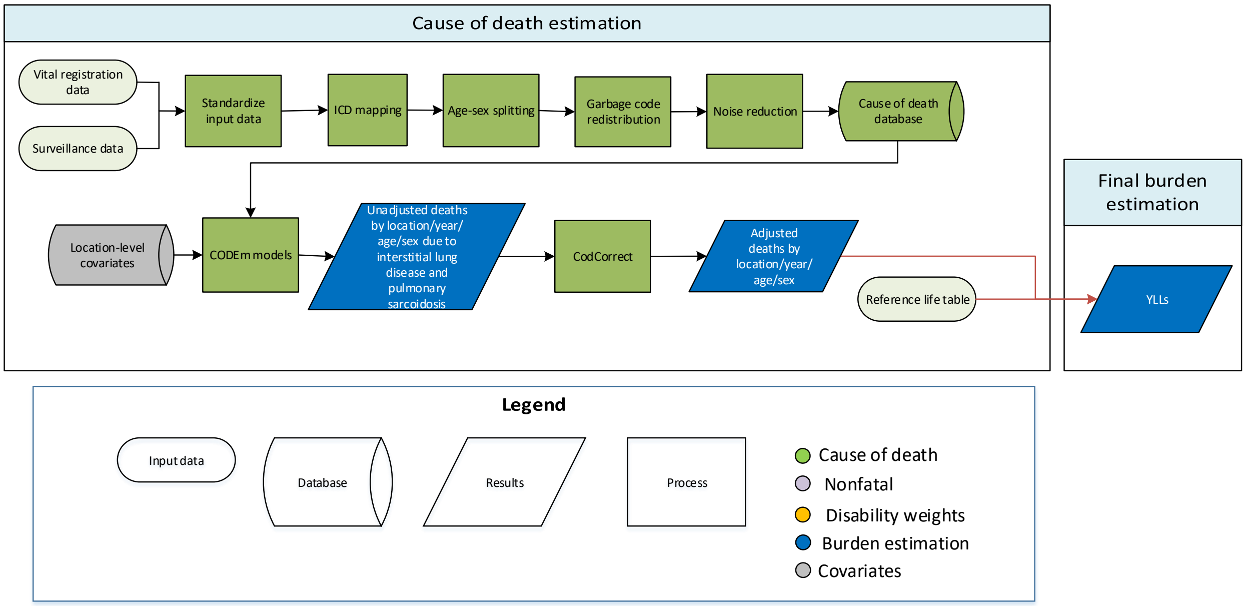

Input data

Data used to estimate interstitial lung disease and pulmonary sarcoidosis mortality included vital registration and surveillance data from the cause of death (COD) database. Our outlier criteria excluded data points that (1) were implausibly high or low, (2) substantially conflicted with established age or temporal patterns, or (3) significantly conflicted with other data sources conducted from the same locations or locations with similar characteristics (ie, Socio-demographic Index).

Modelling strategy

The standard CODEm modelling approach was applied to estimate deaths due to interstitial lung disease and pulmonary sarcoidosis. Separate models were conducted for male and female mortality, and the age range for both models was 1–95+ years. The mortality estimates from the interstitial lung disease and pulmonary sarcoidosis models were ultimately fit into the chronic respiratory envelope.

The same covariates from GBD 2016 were used.

Level	Covariate	Direction
1	log-transformed SEV scalar: interstitial lung disease	+
	smoking prevalence	+
	cumulative cigarettes (5 years)	+
2	elevation over 1,500m (proportion)	+
	elevation between 500 and 1,500m (proportion)	+

	population density over 1,000 ppl/km^2 (proportion)	+
	indoor air pollution (all cooking fuels)	+
	outdoor air pollution ($PM_{2.5}$)	+
	healthcare access and quality index	-
3	log LDI (I$ per capita)	-
	education (years per capita)	-
	Socio-demographic Index	-

Other Chronic Respiratory Diseases

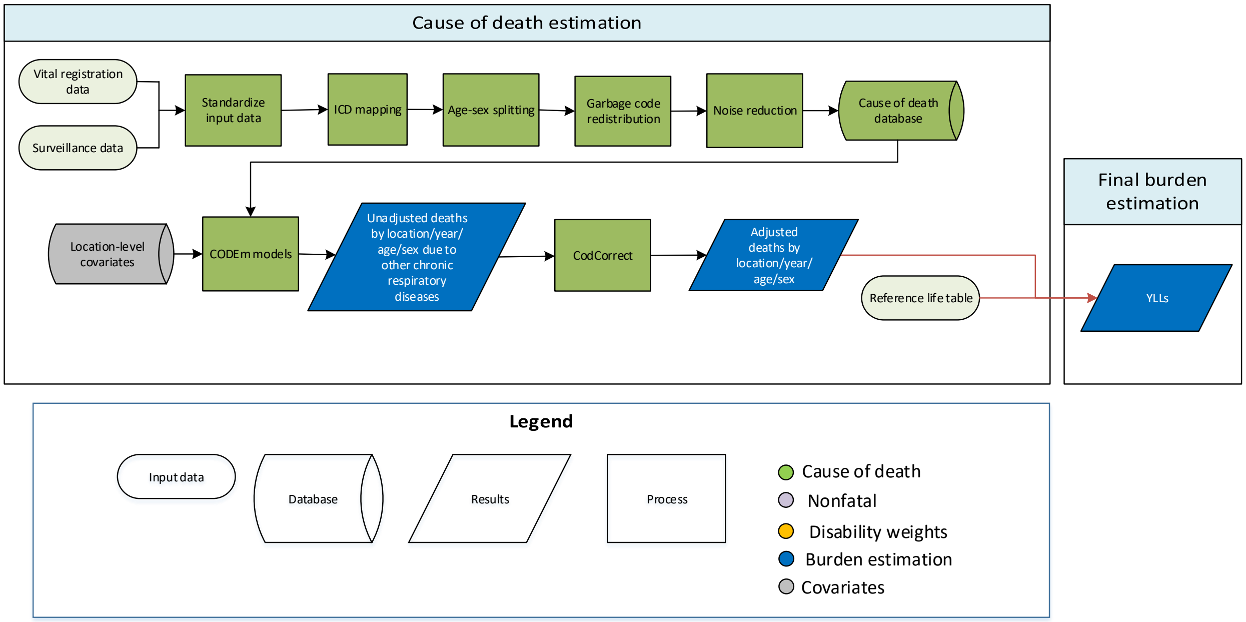

Input data

Data used to estimate other chronic respiratory diseases included vital registration and surveillance data from the cause of death (COD) database. Our outlier criteria excluded data points that (1) were implausibly high or low, (2) substantially conflicted with established age or temporal patterns, or (3) significantly conflicted with other data sources conducted from the same locations or locations with similar characteristics (ie, Socio-demographic Index).

Modelling strategy

The standard CODEm modelling approach was applied to estimate deaths due to other chronic respiratory diseases. Separate models were conducted for male and female mortality, and the age range for both models was 1 year to 95+ years. Like other respiratory causes, the mortality estimates from other chronic respiratory diseases were ultimately fit into the chronic respiratory envelope.

The same covariates from GBD 2016 were used.

Level	Covariate	Direction
1	log-transformed SEV scalar: other chronic respiratory diseases	+
	smoking prevalence	+
	cumulative cigarettes (5 years)	+
	indoor air pollution (all cooking fuels)	+
	outdoor air pollution ($PM_{2.5}$)	+

2	elevation over 1,500m (proportion)	+
	elevation between 500 and 1,500m (proportion)	+
	population density over 1,000 ppl/km^2 (proportion)	+
	healthcare access and quality index	-
3	log LDI (I$ per capita)	-
	education (years per capita)	-
	Socio-demographic Index	-

Chronic obstructive pulmonary disease (COPD)

Flowchart

Chronic Obstructive Pulmonary Disease (COPD)

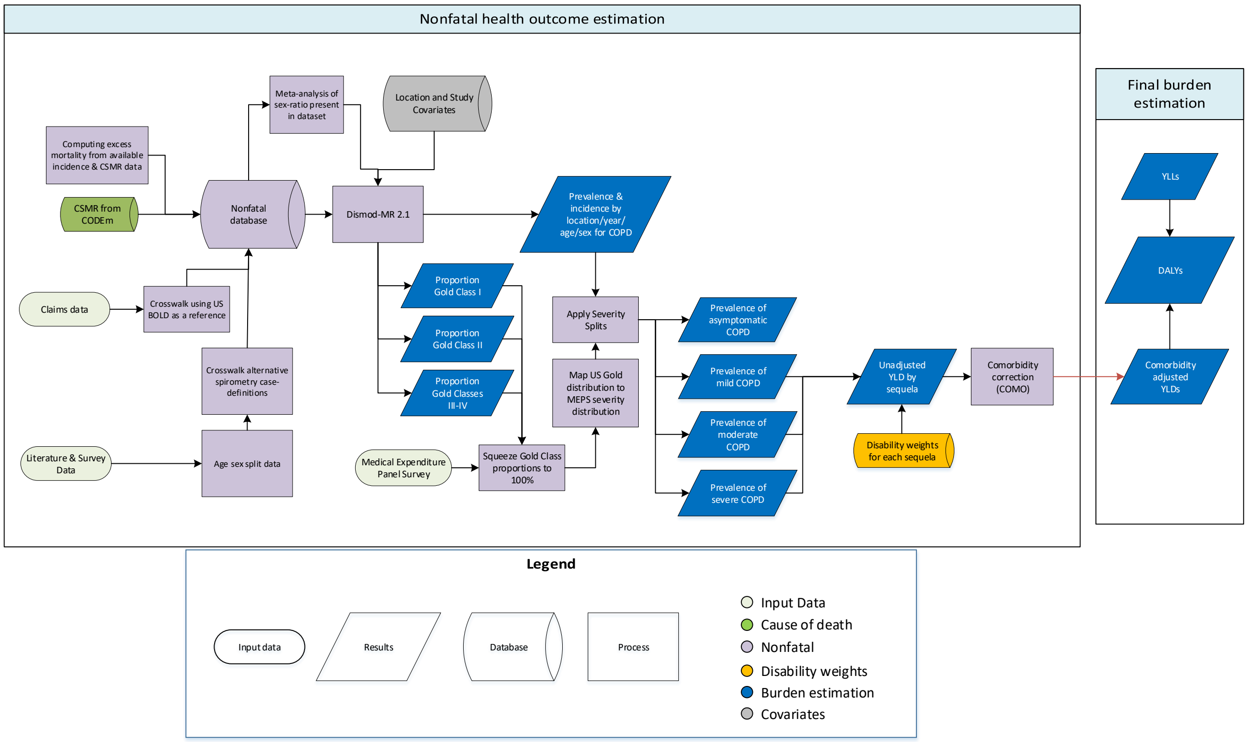

Input data and methodological summary

Case definition

COPD is defined as in the Global Initiative for Chronic Obstructive Lung Disease (GOLD) classification: a measurement of <0.7 FEV_1/FVC (one second of forceful exhalation/total forced expiration) on spirometry after bronchodilation. It should be noted that this is the same reference definition as was used for GBD 2015 and GBD 2016, but it is different from GBD 2013, where the "Lower Limit of Normal (LLN)," ie, relative to an age- and sex-specific norm for the FEV_1/FVC ratio, was the reference. We made this decision because the severity grading of COPD follows the GOLD Class definition rather than the LLN concept. The definitions of the severity classes in the GOLD classification are provided below.

GOLD CLASS	FEV_1 Score
I: Mild	>=80% of normal
II: Moderate	50-79% of normal
IV: Severe	<50% of normal

ICD-10 codes associated with COPD include J41, J42, J43, J44, and J47. The corresponding ICD-9 codes are 491-492, and 496. J40 & 490 (Bronchitis, not specified as acute or chronic) and J47 & 494 (Bronchiectasis) were mapped to COPD for GBD 2016 but excluded for GBD 2017 based on expert feedback.

Input data

No systematic review of the literature was completed for GBD 2017; however, for GBD 2016, we updated the systematic review from previous iterations. The full search term was:

(chronic obstructive pulmonary disease[Title/Abstract] AND (prevalence[Title/Abstract] or incidence [Title/Abstract] or mortality [Title/Abstract] or death [Title/Abstract]) AND "Cross-Sectional Studies"[MeSH Terms]) Filters: Publication date from 04/01/2015 to 11/01/2016; Humans

For GBD 2017, we reviewed the papers listed in the following meta-analysis of COPD prevalence estimates:

Adeloye D, Chua S, Lee C, Basquill C, Papana A, Theodoratou E, Nair H, Gasevic D, Sridhar D, Campbell H, Chan KY. Global and regional estimates of COPD prevalence: Systematic review and meta–analysis. Journal of global health. 2015 Dec;5(2).

In addition to scientific literature, we included survey data with spirometry measurements, such as the National Health and Nutrition Examination Study series in the United States. The Study of Aging and Global Health (SAGE) series, the Korean NHANES, the English Longitudinal Study of Aging (ELSA), and the Turkey Chronic Diseases and Risk Factors Study 2011 were all added for GBD 2017.

Data using alternative case-definitions of COPD prevalence (ie, LLN or FEV1/FVC<0.7 pre-bronchodilator) were crosswalked to the reference case-definition with age-specific ratios derived from studies reporting prevalence using both the alternative and reference case-definitions.

Furthermore, claims data for the United States were included. Additional information on the claims data collection and pre-corrections are provided elsewhere. Briefly, we determined USA national and state-level estimates of COPD prevalence from a database of individual-level ICD-coded health service encounters. Persons with any inpatient claim or at least two outpatient claims associated with COPD were marked as a prevalent case for that year.

For GBD 2016, a correction was made for COPD USA claims data. Under the assumption that NHANES estimates are more accurate than claims data estimates because they use spirometry measurements, we derived an age-specific crosswalk to adjust USA claims data according to the ratio between NHANES and the national-level USA claims estimates. However, for GBD 2017 we decided the age-pattern apparent in NHANES is unreliable and perhaps implausibly high in individuals under 30 years old, due to the fact that NHANES spirometry measurements are taken without the use of a bronchodilator. Instead, we derived an age-specific crosswalk using a comparison of BOLD study results from Kentucky to claims data from Kentucky. Claims data are valuable for the subnational variation they can provide; however, the challenge of correcting the systematic bias present in claims data relative to spirometry-based prevalence data has no clear or singular resolution.

The volume of claims data is sufficiently large to have a ripple effect throughout the model. One way this effect manifests is in the sex-ratio. The GBD 2016 NHANES-based crosswalk was both age and sex-specific. The GBD 2017 BOLD-based crosswalk, on the other hand, is not sex-specific, and this decision was made because BOLD estimates in Kentucky are greater in females than in males, whereas USA NHANES and claims data suggest greater prevalence in females. As a result of using a non-sex-specific crosswalk, the sex-ratio present in the claims data is preserved by the crosswalk. This ratio, while in the direction we expect (larger prevalence in males), is smaller in magnitude than the ratio from NHANES, and therefore smaller than the ratio present in our adjusted data for GBD 2016. This modelling decision had the effect of increasing prevalence in females in the US, and this, combined with new UK data that are higher in females than the GBD 2016 models, resulted in higher modelled prevalence for females in many other GBD regions as well. A table describing the density and distribution of the available data informing the COPD estimation process is provided below.

	Prevalence	Incidence	Proportion by GOLD class
Site-years (total)	504	5	39
Number of countries with data	53	5	31
Number of GBD regions with data (out of 21 regions)	16	3	15
Number of GBD super-regions with data (out of 7 super-regions)	7	2	7

Modelling strategy

As described above, the estimation of COPD burden occurs in three main steps. The first is the estimation of prevalence and incidence using a DisMod-MR 2.1 model. The second is the separate estimation of the proportions by three GOLD class groupings in DisMod-MR 2.1. The third is the combination of these two processes to derive prevalence by severity.

Step 1: Main COPD model

Prior settings include remission of 0 and an incidence ceiling of 0.0002 before age 20. The latter was necessary to avoid a kick-up of estimates in childhood at an age range with few or no primary data.

Similar to other causes, we included estimates of cause-specific mortality rate (CSMR) and derived estimates of excess mortality rate (EMR) by dividing every prevalence data point by the CSMR value for the corresponding location, age, sex, and year. We did not estimate EMR for data points with an age range greater than 20 years.

To assist estimation, each model includes a series of country-level covariates that describe spatiotemporal patterns. For example, we use the COPD standardised exposure variables (SEV), which

aggregates multiple risk factors into a single variable. We also use the log of LDI and the Healthcare Access and Quality (HAQ) index on EMR to capture country-level variation of EMR, assuming a negative coefficient (ie, lower mortality with rising GDP and HAQ). For this GBD cycle, the proportion of elevation over 1500m was also added as a country-level covariate on prevalence and EMR based on its significance in the COPD cause of death models.

For GBD 2017, with the new adjustment strategy for claims data, it appeared that DisMod was calculating a sex-coefficient that placed too much weight on the sex-ratio from the claims. The claims ratio is smaller than the ratio from the remainder of the dataset, so this had an undesirable effect. In response, we performed a random-effects meta-analysis of the male:female ratio in our dataset and fixed the sex-coefficient in the DisMod prevalence model accordingly.

Step 2: GOLD class models
The GOLD class models use data from surveys that specified prevalence by GOLD class after expressing the values as a proportion of all COPD cases. For GBD 2016 we used fixed effects from the SEV scalar and the log of lag-distributed income (LDI) per capita to assist estimation. For GBD 2017, we dropped these covariates because they did not produce significant coefficients. We also restricted random effects to +/-0.5 to control implausible geographical variation.

Table of model coefficients for COPD

Model	Variable name	Measure	Beta	Exponentiated
COPD	Elevation over 1500m (proportion)	excess mortality rate	0.21 (0.12–0.31)	1.23 (1.12–1.36)
COPD	LDI (I$ per capita)	excess mortality rate	-0.5 (-0.5 to -0.5)	0.61 (0.60–0.61)
COPD	Log age-standardised SEV scalar: COPD	prevalence	0.90 (0.90–0.90)	2.46 2.46–2.46)

Severity

The three GOLD class groupings reflect a grading based on a physiological measurement rather than a direct measurement of disease severity. In order to map the epidemiological findings by GOLD class into the three COPD health states for which we have disability weights (DW), we used the 2001–2011 Medical Expenditure Panel Survey (MEPS) data from the United States. Specifically, we convert the GOLD class designations estimated for the USA in 2005 (the midpoint of MEPS years of analyses) into GBD classifications of asymptomatic, mild, moderate, and severe COPD.

The table below shows the three health states of COPD and the corresponding lay descriptions and disability weights. The graph shows the average proportion by GOLD class (after scaling to 100%) across all ages for USA in 2005. We also show the proportion of MEPS respondents reporting any health service contact in the past year for COPD with a DW value attributable to COPD of 0, mild range (0 to midpoint between DWs for mild and moderate), moderate range (midpoint of DW values mild and moderate to midpoint of DW values for moderate and severe) and severe range (midpoint between DW values

moderate and severe or higher). The DW value for COPD was derived from a regression with indicator variables for all health states reported by MEPS respondents and their reported overall level of disability derived from a conversion of 12-Item Short Form Surveys (SF-12) answers to GBD DW values. This analysis gave the severity distribution for each GBD cause reported in MEPS after correcting for any comorbid causes individual respondents reported during a year.

Health state	Lay description	DW (95% CI)
Mild COPD	This person has cough and shortness of breath after heavy physical activity, but is able to walk long distances and climb stairs.	0.019 (0.011–0.033)
Moderate COPD	This person has cough, wheezing, and shortness of breath, even after light physical activity. The person feels tired and can walk only short distances or climb only a few stairs.	0.225 (0.153–0.31)
Severe COPD	This person has cough, wheezing, and shortness of breath all the time. The person has great difficulty walking even short distances or climbing any stairs, feels tired when at rest, and is anxious.	0.408 (0.273–0.556)

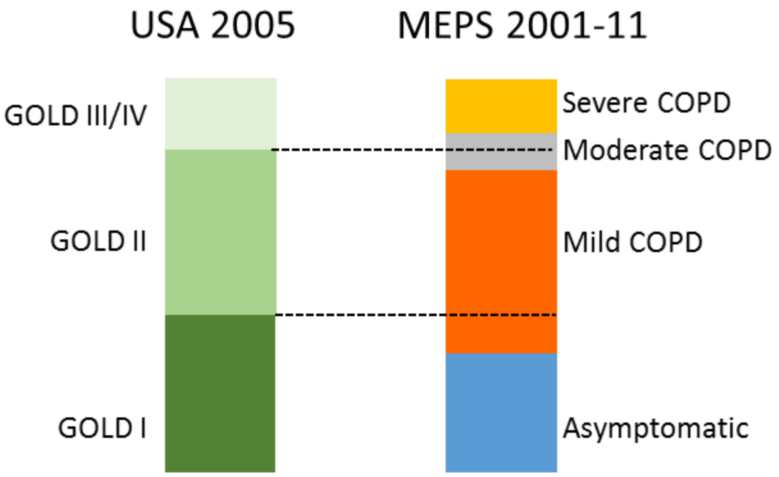

The algorithm to translate GOLD class to COPD DW categories first assigns GOLD III&IV to severe COPD and what remains to moderate. Next, GOLD class I is assigned to the asymptomatic category first and what remains goes to mild COPD. This algorithm is repeated for each age and sex category and for all 1,000 draws from the DisMod models of GOLD classes and the MEPS analyses. We end up with proportions of each of the GOLD class categories that map onto GBD COPD health states with uncertainty bounds determined by the 25th and 975th values of the 1,000 draws. These values are then applied to the estimates of the proportion of cases by GOLD class category, after scaling to 100%, by location, year, age,

and sex. This assumes that the relationship between GOLD class and GBD COPD health states in the United States applies everywhere.

Pneumoconiosis

Coal Worker's Pneumoconiosis, Asbestosis, Silicosis, and Other Pneumoconiosis

Flowchart

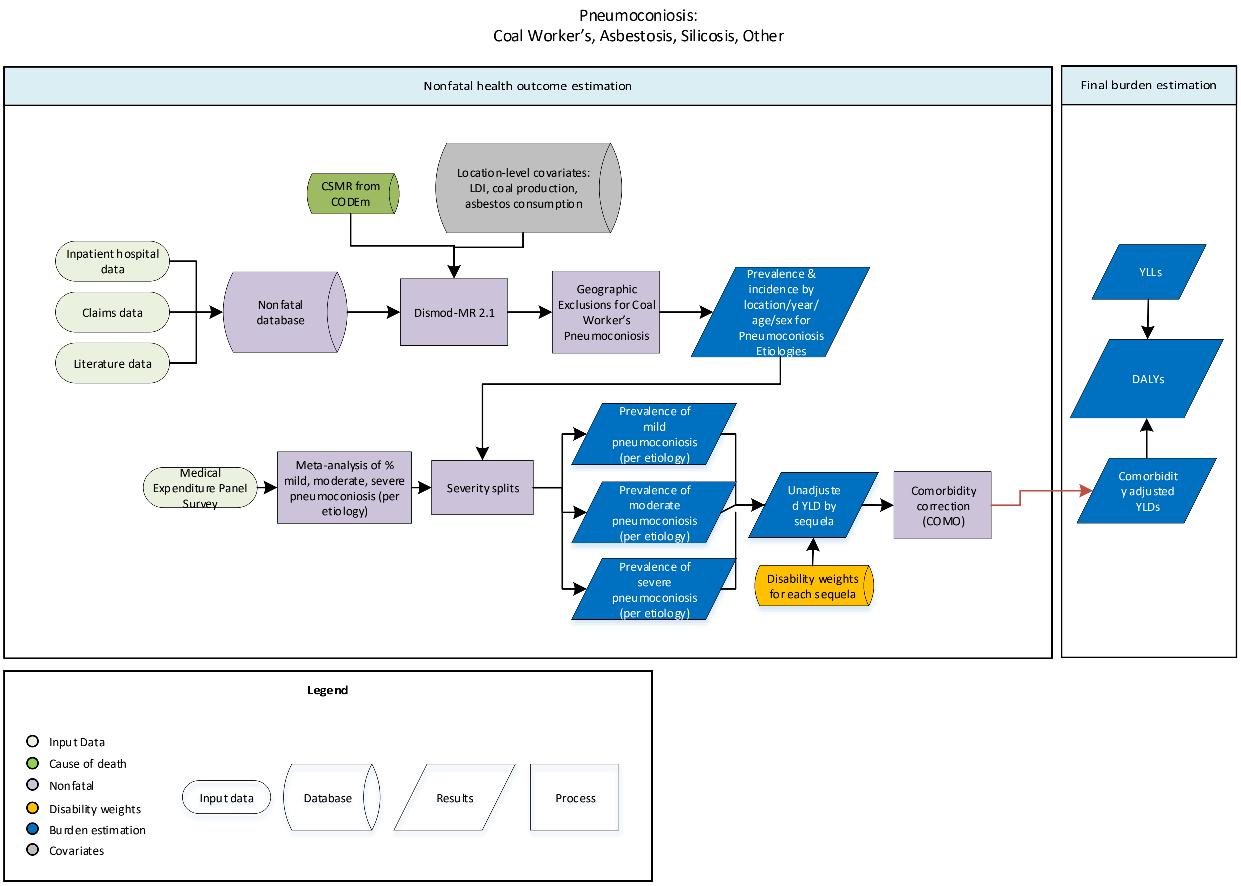

Input data and methodological appendix

Case definition

Pneumoconiosis is a chronic lung disease typified by lung scarring and other interstitial damage caused by exposure to dust and other containments – usually through occupational exposure. For GBD, we model pneumoconiosis by exposure type: coal, asbestos, silica, and other.

Input data

Data used to make estimates of pneumoconiosis are predominantly from three main sources. The first is literature data from systematic reviews, usually from smaller-scale studies of prevalence. One challenge

with literature data is that most studies are conducted in high-risk populations that are not representative of the general population. No systematic review of the literature was conducted for GBD 2017. The second source of data is inpatient hospital reports, and the third is claims data for the United States and Taiwan. For all aetiologies, we use a sex-specific correction factor of the hospital inpatient data where numbers are adjusted upward by the ratio of primary diagnosis to secondary diagnosis present in the claims data. Greater detail on the preparation of the inpatient and claims data is provided elsewhere.

The table below includes details regarding input data counts. All data are for prevalence. Data which have been marked as outliers are not included in these counts.

	Asbestosis	Coal worker's Pneumoconiosis	Silicosis	Other Pneumoconiosis
Site-years (total)	945	769	744	934
Number of countries with data	32	29	33	38
Number of GBD regions with data (out of 21 regions)	12	13	13	15
Number of GBD super-regions with data (out of 7 super-regions)	5	6	6	7

Severity split inputs

Data to inform estimates of the severity gradient due to pneumoconiosis etiologies are derived from previous analyses of the Medical Expenditure Panel Survey (MEPS). The disability weights are also shared.

Severity level	Lay description	DW (95% CI)
Mild	Has cough and shortness of breath after heavy physical activity, but is able to walk long distances and climb stairs.	0.019 (0.011–0.033)
Moderate	Has cough, wheezing, and shortness of breath, even after light physical activity. The person feels tired and can walk only short distances or climb only a few stairs.	0.225 (0.153–0.312)
Severe	Has cough, wheezing, and shortness of breath all the time. The person has great difficulty walking even short distances or climbing any stairs, feels tired when at rest, and is anxious.	0.408 (0.273–0.556)

Modelling strategy

Estimates for the pneumoconiosis aetiologies are produced using a standard DisMod-MR 2.1 approach.

For all aetiologies, we use prior settings of zero remission. Additionally, we assume no incidence and prevalence before the age of 10.

To assist estimation, each model includes a series of country-level covariates that describe spatiotemporal patterns. The standardised exposure variable (SEV) covariates, which were used for GBD 2016, were removed because the associated risk-outcome pairs for the new calculation resulted in undefined SEV values. However, we added the SEV scalar for mesothelioma in the asbestosis model, as asbestosis and mesothelioma have a common risk factor in asbestos exposure. The gold production covariate, which was used for the GBD 2016 silicosis model, was removed because DisMod was assigning it implausible coefficient values. Subnational updates were made to coal production and asbestos consumption to account for new subnational locations for GBD 2017.

Cause	Measure	Variable name	Beta	Exponentiated
Asbestosis	Prevalence	Asbestos consumption (per capita)	0.47<br>(0.015–1.70)	1.60<br>(1.02–5.47)
Asbestosis	Prevalence	Log-transformed age-standardised SEV scalar: Mesothelioma	0.029<br>(0.000016–0.32)	1.03<br>(1.00–1.38)
Coal worker's	Prevalence	Coal production (per capita)	0.0017<br>(-0.00025 to 0.0045)	1.00<br>(1.00–1.00)

Prevalence and incidence of coal worker's pneumoconiosis were set to zero in locations without a history of coal mining given the causal and necessary relationship between respective occupational exposure and disease. For GBD 2016 these locations were values with zero coal production for 30 years in the GBD coal production covariate, but for GBD 2017 we cross-referenced these locations with vital registration data to ensure that we are not setting prevalence and incidence to zero for any locations where vital registration codes greater than zero deaths due to coal worker's pneumoconiosis.

Asthma

Flowchart

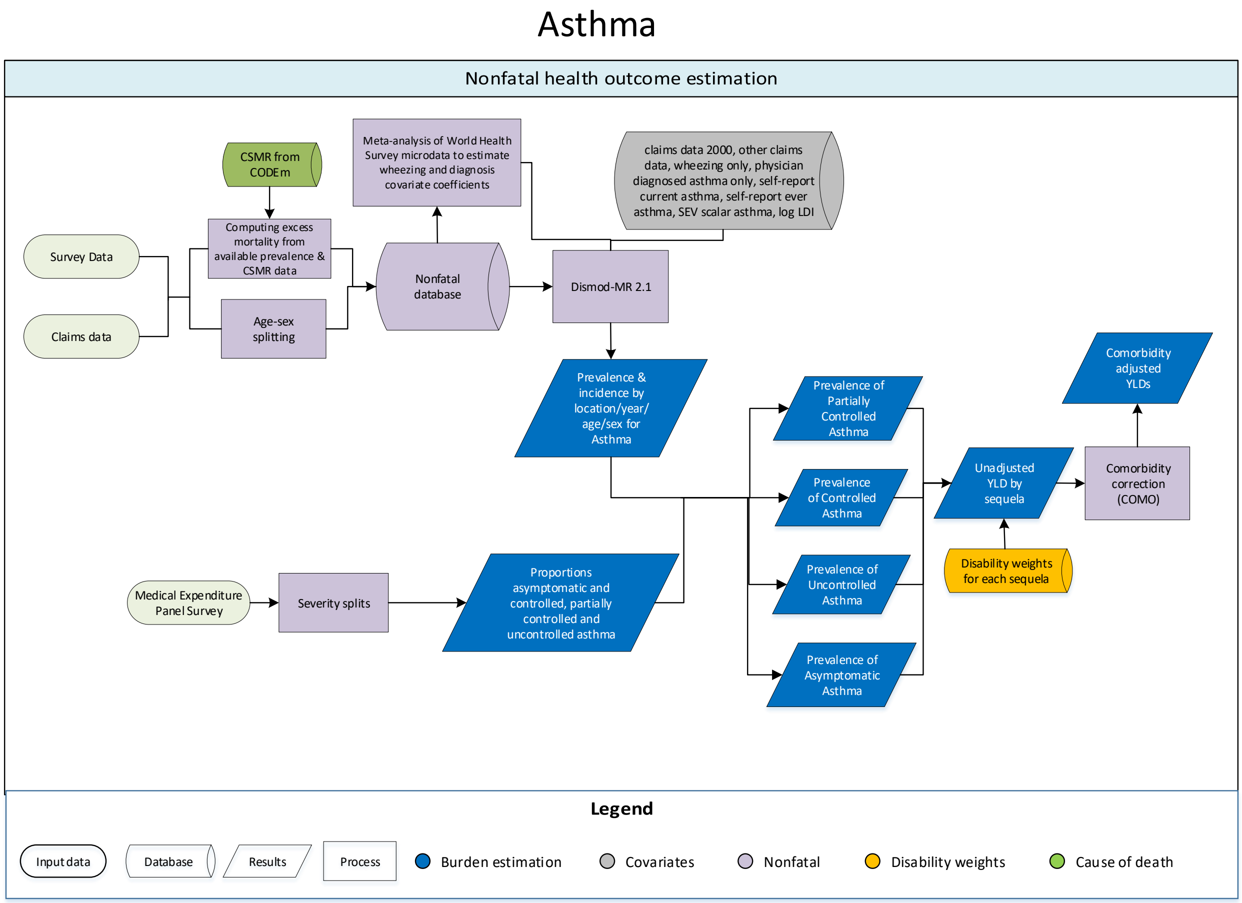

Case definition

Asthma is a chronic lung disease marked by spasms in the bronchi usually resulting from an allergic reaction or hypersensitivity and causing difficulty in breathing. We define asthma as a doctor's diagnosis and wheezing in the past year. The relevant ICD-10 codes are J45 and J46. ICD-9 code is 493.

Input data

No systematic review of the literature was completed for this GBD cycle. However, for GBD 2016, we did a full systematic review of the literature on asthma. We used the following search string in PubMed and filtered by studies of humans published between January 2012 and November 2016.

(Asthma[Title/Abstract] AND prevalence[Title/Abstract] AND "Cross-Sectional Studies"[MeSH Terms])

Survey data added for GBD 2016 include the Survey of Health, Ageing and Retirement in Europe (SHARE), the Russian Ural Eye and Medical Study, the South Africa National Income Dynamics Study, the South Africa General Household Survey 2009, and the WHO Study on Global Ageing and Adult Health series (SAGE), among others.

Surveys carried out as part of the International Study of Asthma and Allergies in Childhood (ISAAC) collaboration are the most important source of prevalence data in children.

The following table provides a description of the data density and distribution by geography and epidemiological measure (including the claims data discussed below).

	Prevalence	Incidence	Remission	Other
Site-years (total)	1389	10	32	9
Number of countries with data	136	5	15	6
Number of GBD regions with data (out of 21 regions)	21	1	7	3
Number of GBD super-regions with data (out of 7 super-regions)	7	1	5	3

In addition to literature and survey data, we use claims data from the United States. Information on the source and preparation of these data are provided in detail elsewhere.

Modelling strategy

We use DisMod-MR 2.1 as the main modelling tool for asthma. Prior settings include a maximum remission of 0.3 (reflecting the upper bound of the highest observed data) and no incidence between the ages of 0 and 0.5 year, as a diagnosis cannot be made in young infants.

Data points from the ISAAC studies were reported for both sexes combined. We sex-split before modelling using the ratios derived from the 2012 US claims data.

Data that describe wheezing in the past year but do not report presence/absence of an accompanying diagnosis are crosswalked to the reference category using a study-level covariate in DisMod. As the table below shows, studies that only report wheezing are systematically higher than reference data points and are adjusted down – dividing by the exponentiated coefficient. Data that describe prevalence of lifetime diagnosis of asthma but not accompanying wheezing in the past year are also crosswalked to the reference category using a study-level covariate. For GBD 2016, we allowed DisMod to estimate these coefficients. For GBD 2017 we performed an analysis of World Health Survey microdata to estimate the coefficients and used these values as priors in the DisMod model.

To account for country-level differences in excess mortality as a function of available medical care we use log lag-distributed income (LDI) as a covariate and assume a negative coefficient. The effect size is shown below.

For GBD 2016, claims data for 2000 and 2010 were adjusted via study covariates to account for systematically lower estimates relative to the 2012 claims data. Implicit in this adjustment is the assumption that variation between years of claims data is a function of data-collection inconsistencies. However, an analysis for GBD 2017 showed that even the 2012 claims data were systematically lower than asthma survey data. To account for this, we estimated a MarketScan 2000 coefficient and a separate MarketScan coefficient for the remaining years of data, by comparing the national values in these datasets to national asthma estimates from the USA National Health and Nutrition Examination Survey and National Health Interview Surveys.

Similar to other causes, we include estimates of cause-specific mortality rate (CSMR) and excess mortality rate (EMR) derived as a matched value for each prevalence data point dividing CSMR by prevalence. We restrict these EMR calculations to data points of 20-year age span or less.

To assist estimation, the model includes a series of country-level covariates that describe spatiotemporal patterns. Specifically, we use log LDI and the asthma standardised exposure variable (SEV), a scalar that combines exposure of all GBD risks that influence asthma. A full covariate list, including the study-level covariates described above, are presented in the following table with their associated effects:

Variable name	Measure	Beta	Exponentiated
Wheezing only	prevalence	1.05<br>(1.05–1.05)	2.85<br>(2.85–2.85)
Physician-diagnosed asthma only	prevalence	0.60<br>(0.60–0.60)	1.82<br>(1.82–1.82)
Self-reported currently have asthma	prevalence	0.22<br>(0.16–0.28)	1.24<br>(1.17–1.32)
Self-reported ever having asthma	prevalence	0.24<br>(0.20–0.28)	1.28<br>(1.23–1.32)
Claims data 2000	prevalence	-1.25<br>(-1.25 to -1.25)	0.29<br>(0.29–0.29)
Claims data post-2000	prevalence	-0.79<br>(-0.79 to -0.79)	0.45<br>(0.45–0.45)
Log SEV scalar: asthma	prevalence	0.75<br>(0.75–0.76)	2.13<br>(2.12–2.14)

Log LDI (I$ per capita)	excess mortality rate	-0.5<br>(-0.5 to -0.5)	0.61<br>(0.61–0.61)

Severity split inputs

Lay descriptions and disability weights for the asthma health states are shown in the table below. The distribution between the three health states is derived from an analysis of the USA Medical Expenditure Panel Surveys (MEPS). The methods are described in full in a separate section of this appendix. Briefly, MEPS is an ongoing survey of health service encounters with as its main objective to collect data on health expenditure. Panels are recruited every year and followed up for a period of two years. Diagnostic information provided by respondents on the reasons for any health care contact are coded into three-digit ICD-9 codes by professional coders.

Twice over the two-year follow-up period, respondents are asked to fill in 12-Item Short Form Surveys (SF-12). From convenience samples asking respondents to fill in SF-12 for 60 of the GBD health states, IHME has created a mapping from SF-12 scores to GBD disability weights (DW). We perform a regression with indicator variables for all GBD causes that we can identify from the ICD codes in MEPS to derive for each individual with a diagnosis the amount of disability that can be attributed to that condition after controlling for any comorbid conditions. Anyone with a diagnosis of asthma in whom the disability assigned to asthma is negative or zero we assume is asymptomatic (at the time of asking SF-12 question relating to their health status in the past four weeks). Non-zero values we bin into the three health states assuming a split between these at the midpoint between DW values. The table below gives the proportions in MEPS in each of the health states and an asymptomatic state.

Severity level	Lay description	DW (95% CI)	Severity distribution
Asymptomatic			36.2%<br>(35.0–37.3%)
Controlled	This person has wheezing and cough once a month, which does not cause difficulty with daily activities.	0.015<br>(0.007–0.026)	19.9%<br>(13.6–27.8%)
Partially controlled	This person has wheezing and cough once a week, which causes some difficulty with daily activities.	0.036<br>(0.022–0.055)	20.6%<br>(15.1–25.8%)
Uncontrolled	This person has wheezing, cough, and shortness of breath more than twice a week, which causes difficulty with daily activities and sometimes wakes the person at night.	0.133<br>(0.086–0.192)	23.3%<br>(18.7–30.3%)

Interstitial lung disease and pulmonary sarcoidosis (ILD)

Flowchart

Interstitial Lung Disease and Pulmonary Sarcoidosis (ILD)

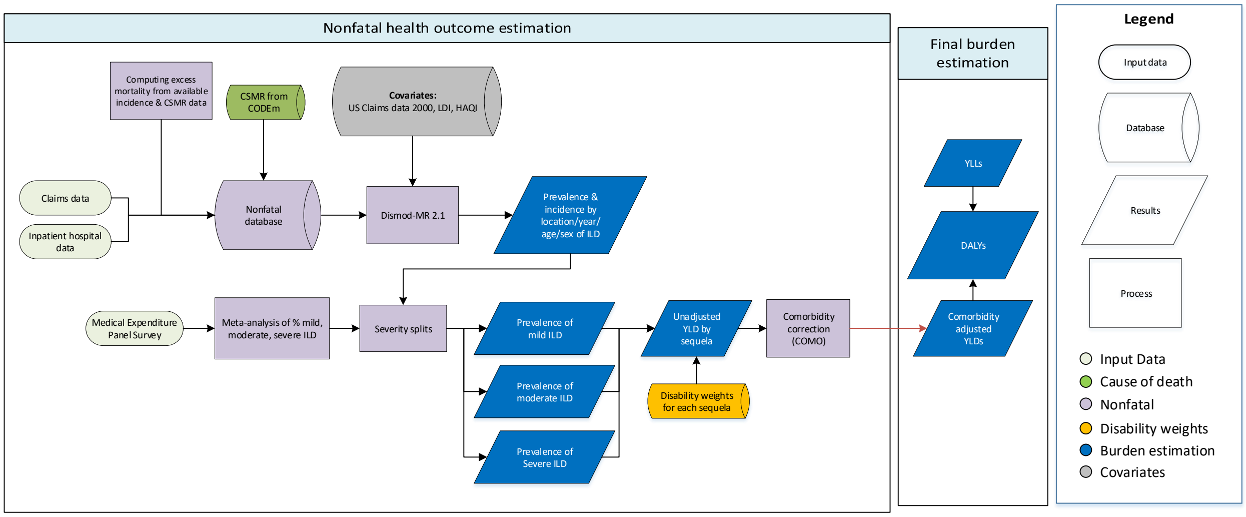

Case definition

Interstitial lung diseases and pulmonary sarcoidosis are a collection of chronic respiratory diseases that impair lung function and oxygen uptake through scarring and/or inflammation. The relevant ICD codes are D86 and J84. For interstitial lung disease, we use the American Thoracic Society as the gold standard definition.

Input data

Model Inputs

No systematic review of the literature was conducted for ILD for this iteration of the Global Burden of Disease. These reviews are done on a rotating basis and updates will be made for a future iteration.

Data used to make estimates of ILD are predominantly from three main sources. The first is literature data from previous systematic reviews – usually from smaller-scale studies of prevalence or incidence. The second main data type is claims data for the United States. The source and preparation of these data is described elsewhere. The third main data type is adjusted hospital inpatient records. Because these records only report primary diagnosis, we a priori adjust the numbers by a sex-specific factor based on the observed ratio between USA claims data and USA inpatient hospital data.

The following table provides a picture of the number of available studies along with their distribution globally and by epidemiological profile. In short, the ILD data landscape is rather sparse. The available data are largely skewed toward high-income countries like the United States or the member countries of

the European Union. The relatively high number of subnational units with data is largely a function of claims data in the United States and hospital data from Mexico and Brazil.

	Prevalence	Incidence	Other
Site-years (total)	1380	54	2
Number of countries with data	39	16	2
Number of GBD regions with data (out of 21 regions)	15	7	2
Number of GBD super-regions with data (out of 7 super-regions)	7	4	2

Severity splits

Data to inform estimates of the severity gradient due to ILD are derived from previously analyses of the Medical Expenditure Panel Survey (MEPS). The table below illustrates the lay descriptions and disability weights associated with different levels of severity of interstitial lung disease.

Severity level	Lay description	DW (95% CI)
Mild	Has cough and shortness of breath after heavy physical activity, but is able to walk long distances and climb stairs.	0.019 (0.011–0.033)
Moderate	Has cough, wheezing, and shortness of breath, even after light physical activity. The person feels tired and can walk only short distances or climb only a few stairs.	0.225 (0.153–0.312)
Severe	Has cough, wheezing, and shortness of breath all the time. The person has great difficulty walking even short distances or climbing any stairs, feels tired when at rest, and is anxious.	0.408 (0.273–0.556)

Modelling strategy

Estimates for ILD are produced using a standard DisMod-MR 2.1 approach. We use prior settings of zero remission and we constrain the super-region random effects to -0.5 to 0.5 to ensure model stability.

As described above, we use an a priori adjustment of hospital inpatient data.

Similar to other causes, we include estimates of cause-specific mortality rate (CSMR) and Excess Mortality Rate (EMR). The source and estimation of these rates are discussed elsewhere.

Variable name	Measure	Beta	Exponentiated
All MarketScan, year 2000	prevalence	-0.25<br>(-0.27 to -0.23)	0.78<br>(0.76–0.79)
LDI (I$ per capita)	excess mortality rate	-0.2<br>(-0.2 to -0.2)	0.82<br>(0.82–0.82)
Healthcare Access and Quality index	excess mortality rate	0.012<br>(0.012–0.013)	1.01<br>(1.01–1.01)

A study-level covariate was used for MarketScan 2000 data to adjust for systematically low values. To account for country-level differences in excess mortality (perhaps as a function of available medical care) we use ln(lag distributed income) and Healthcare Access and Quality (HAQ) index as proxy measures. The effect sizes are shown above.

Other chronic respiratory diseases

In addition to the chronic respiratory diseases described above, there are many diverse types of chronic respiratory diseases with a range of severities and associated sequelae. Because these chronic respiratory diseases are diverse in their underlying causes and risk factors as well as in their associated health outcomes, modelling them together in a DisMod-MR model would not produce reliable estimates of prevalence or excess mortality. Instead, we calculated the YLDs caused by other chronic respiratory diseases directly using a YLD/YLL ratio.

We calculated the ratio of YLDs to YLLs across the specified chronic respiratory diseases for which non-fatal outcomes were modelled, using YLL estimates from the GBD 2017 cause of death (CoD) analysis. We then multiplied this YLD/YLL ratio by the YLL estimates for other chronic respiratory diseases from the GBD 2017 CoD analysis, providing us with an estimate of the YLDs associated with other chronic respiratory diseases.

Online Methods risks for individual write-ups for respiratory risk factors in GBD 2017

Smoking Capstone Appendix

Flowchart

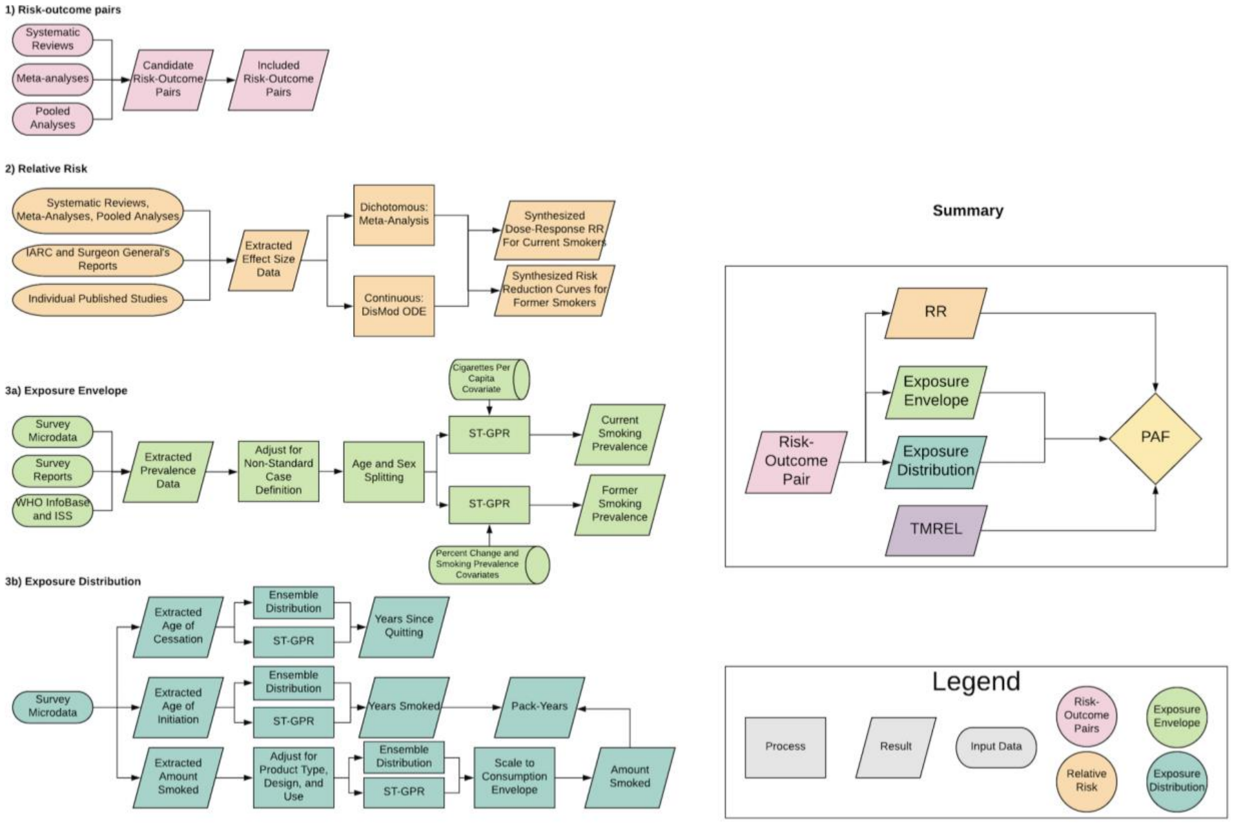

We made significant changes to the methods used to estimate smoking attributable burden in GBD 2017. In previous iterations of the GBD, we have used the Peto-Lopez (Smoking Impact Ratio) method to estimate burden attributable to cancers and chronic respiratory diseases. Although this method provides robust estimates of the burden of cancers and chronic respiratory diseases related to tobacco, it is not fully consistent with the GBD approach of estimating exposure independently of the outcomes affected by exposure. For cardiovascular diseases and all other smoking attributable health outcomes, we used five-year lagged daily smoking prevalence as the exposure. With a growing body of evidence on the association between smoking and several types of cancers and with cardiovascular disease, coupled with good estimates of the distribution of cumulative smoking exposure, direct estimation of attributable burden is possible. In GBD 2017, we have transitioned to using continuous measures of exposure that incorporate dose-response effects among daily, occasional, and former smokers for all health outcomes except fractures.

Current and former smoking prevalence

We estimated the prevalence of current smoking and the prevalence of former smoking using data from cross-sectional nationally representative household surveys. We defined current smokers as individuals who currently use any smoked tobacco product on a daily or occasional basis. We defined former smokers as individuals who quit using all smoked tobacco products for at least 6 months, where possible, or according to the definition used by the survey. Prior to modelling a complete time series for all demographic groups, we made adjustments for alternative case definitions as well as for data reported in non-standard age or sex groups. We modelled current and former prevalence using spatiotemporal Gaussian process regression.

Data extraction

We extracted primary data from individual-level microdata and survey report tabulations. We extracted data on current, former, and/or ever smoked tobacco use reported as any combination of frequency of use (daily, occasional, and unspecified, which includes both daily and occasional smokers) and type of smoked tobacco used (all smoked tobacco, cigarettes, hookah, and other smoked tobacco products such as cigars or pipes), resulting in 36 possible combinations. Other variants of tobacco products, for example hand-rolled cigarettes, were grouped into the four type categories listed above based on product similarities. Only smoked tobacco products are included, smoked drugs are estimated separately as part of the drug use risk factor.

For microdata, we extracted relevant demographic information, including age, sex, location, and year, as well as survey metadata, including survey weights, primary sampling units, and strata. This information allowed us to tabulate individual-level data in the standard GBD five-year age-sex groups and produce accurate estimates of uncertainty. For survey report tabulations, we extracted data at the most granular age-sex group provided.

Crosswalk

Our GBD smoking case definitions were current smoking of any tobacco product and former smoking of any tobacco product. All other data points were adjusted to be consistent with either of these definitions. Some sources contained information on more than one case definition and these sources were used to develop the adjustment coefficient to transform alternative case definitions to the GBD case definition. The adjustment coefficient was the beta value derived from a linear model with one predictor and no intercept.

We generated separate crosswalk coefficients for the 10-14 age group and the 15-19 age group, as we found the relationships between case definitions differed strongly in the younger age groups compared to the 20+ age groups. To account for this, we attempted to generate a global crosswalk coefficient for both the 10-14 and 15-19 age groups, using the same regression as above. Due to data limitations, none of the crosswalk coefficients met the criteria outlined above, so no data covering youths under 20 years old were crosswalked. In other words, all data from these age groups that appear in the model were asked according to our case definition in the survey.

We propagated uncertainty at the survey level from the crosswalk by incorporating both the variance of the errors and the variance of the adjustment coefficients.

For each source that needed adjusting, we assigned space weights based on GBD region and super region to the sources containing more than one case definition. Data from the same region receiving a

full weight of 1, and data from the same super-region received a weight of ½. We explored using a time weight, to control for possible changes in the relationship between smokeless tobacco use behaviours over time. We found incorporating temporal information did not significantly change the estimated coefficients but did undercut sample sizes, and chose to exclude the time weight. Crosswalk coefficients generated from fewer than 20 data sources were dropped

Age and sex splitting

We split data reported in broader age groups than the GBD 5-year age groups or as both sexes combined by adapting the method reported in Ng et al. (http://jamanetwork.com/journals/jama/fullarticle/1812960) to split using a sex- geography- time specific reference age pattern. We separated the data into two sets: a training dataset, with data already falling into GBD sex-specific 5-year age groups, and a split dataset, which reported data in aggregated age or sex groups. We then used spatiotemporal Gaussian Process Regression (ST-GPR) to estimate sex-geography-time specific age patterns using data in the training dataset. The estimated age patterns were used to split each source in the split dataset.

The ST-GPR model used to estimate the age patterns for age-sex splitting used an age weight parameter value that minimises the effect of any age smoothing. This parameter choice allows the estimated age pattern to be driven by data, rather than being enforced by any smoothing parameters of the model. Because these age-sex split data points will be incorporated in the final ST-GPR exposure model, we do not want to doubly enforce a modelled age pattern for a given sex-location-year on a given aggregate data point.

Smoking prevalence modelling

We used ST-GPR to model current and former smoking prevalence. Full details on the ST-GPR method are reported elsewhere in the Appendix. Briefly, the mean function input to GPR is a complete time series of estimates generated from a mixed effects hierarchical linear model plus weighted residuals smoothed across time, space and age. The linear model formula for current smoking, fit separately by sex using restricted maximum likelihood in R, is:

$$logit(p_{g,a,t}) = \beta_0 + \beta_1 CPC_{g,t} + \sum_{k=2}^{19} \beta_k I_{A[a]} + \alpha_s + \alpha_r + \alpha_g + \epsilon_{g,a,t}$$

Where $CPC_{g,t}$ is the tobacco consumption covariate by geography g and time t, described above, $I_{A[a]}$ is a dummy variable indicating specific age group A that the prevalence point $p_{g,a,t}$ captures, and α_s, α_r, and α_g are super region, region, and geography random intercepts, respectively. Random effects were used in model fitting but not in prediction.

The linear model formula for former smoking is:

$$logit(p_{g,a,t}) = \beta_0 + \beta_1 PctChange_{A[a],g,t} + \beta_3 CSP_{A[a],g,t} + \sum_{k=3}^{20} \beta_k I_{A[a]} + \alpha_s + \alpha_r + \alpha_g + \epsilon_{g,a,t}$$

Where $PctChange_{A[a],g,t}$ is the percent change in current smoking prevalence from the previous year, and $CSP_{A[a],g,t}$ is the current smoking prevalence by specific age group A, geography g, and time t that point $p_{g,a,t}$ captures, both derived from the current smoking ST-GPR model defined above.

Exposure among current and former smokers

We estimated exposure among current smokers for two continuous indicators: cigarettes per smoker per day and pack-years. Pack-years incorporates aspects of both duration and amount. One pack-year represents the equivalent of smoking one pack of cigarettes (assuming a 20 cigarette pack) per day for one year. Since the pack-years indicator collapses duration and intensity into a single dimension, one pack-year of exposure can reflect smoking 40 cigarettes per day for six months or smoking 10 cigarettes per day for two years.

To produce these indicators, we simulated individual smoking histories based on distributions of age of initiation and amount smoked. We informed the simulation with cross-sectional survey data capturing these indicators, modelled at the mean level for all locations, years, ages, and sexes using spatiotemporal Gaussian process regression. We rescaled estimates of cigarettes per smoker per day to an envelope of cigarette consumption based on supply-side data. We estimated pack-years of exposure by summing samples from age- and time-specific distributions of cigarettes per smoker for a birth cohort in order to capture both age trends and time trends and avoid the common assumption that the amount someone currently smokes is the amount they have smoked since they began smoking. All distributions were age-, sex-, and region- specific ensemble distributions, which were found to outperform any single distribution.

We estimated exposure among former smokers using years since cessation. We utilised spatiotemporal Gaussian process regression to model mean age of cessation using cross-sectional survey data capturing age of cessation. Using these estimates, we generated ensemble distributions of years since cessation for every location, year, age group, and sex.

Risk-outcome pairs

We included the following risk-outcome pairs based on evidence supporting a causal relationship: tuberculosis, lower respiratory tract infections, esophageal cancer, stomach cancer, bladder cancer, liver cancer, laryngeal cancer, lung cancer, breast cancer, cervical cancer, colorectal cancer, lip and oral cancer, nasopharyngeal cancer, other pharyngeal cancer, pancreatic cancer, kidney cancer, leukemia, ischemic heart disease, ischemic stroke, hemorrhagic stroke, subarachnoid hemorrhage, atrial fibrillation and flutter, aortic aneurysm, peripheral arterial disease, chronic obstructive pulmonary disease, other chronic respiratory diseases, asthma, peptic ulcer disease, gallbladder and biliary tract diseases, Alzheimer disease and other dementias, Parkinson disease (protective), multiple sclerosis, type-II diabetes, rheumatoid arthritis, low back pain, cataracts, macular degeneration, and fracture.

Dose-response risk curves

We conducted systematic literature reviews for all risk-outcome pairs identified as being caused by smoking. We extracted effect sizes by cigarettes per smoker per day, pack-years, and years since quitting from cohort and case-control studies. We synthesised these data to produce non-linear dose response curves using a Bayesian meta-regression model. For outcomes with significant differences in effect size by sex or age, we produced sex- or age-specific risk curves.

We estimate risk curves of former smokers compared to never smokers taking into account the rate of risk reduction among former smokers seen in the cohort and case-control studies, and the cumulative exposure among former smokers within each age, sex, location and year group.

PAF calculation

We estimated population attributable fractions based on the following equation:

$$PAF = \frac{p(n) + p(f) \int \exp(x) * rr(x) + p(c) \int \exp(y) * rr(y) - 1}{p(n) + p(f) \int \exp(x) * rr(x) + p(c) \int \exp(y) * rr(y)}$$

where $p(n)$ is the prevalence of never smokers, $p(f)$ is the prevalence of former smokers, $p(c)$ is the prevalence of current smokers, $\exp(x)$ is a distribution of years since quitting among former smokers, $rr(x)$ is the relative risk for years since quitting, $\exp(y)$ is a distribution of cigarettes per smoker per day or pack-years, and $rr(y)$ is the relative risk for cigarettes per smoker per day or pack-years.

We used pack-years as the exposure definition for cancers and chronic respiratory diseases, and cigarettes per smoker per day for cardiovascular diseases and all other health outcomes.

Secondhand Smoke Capstone Appendix

Flowchart

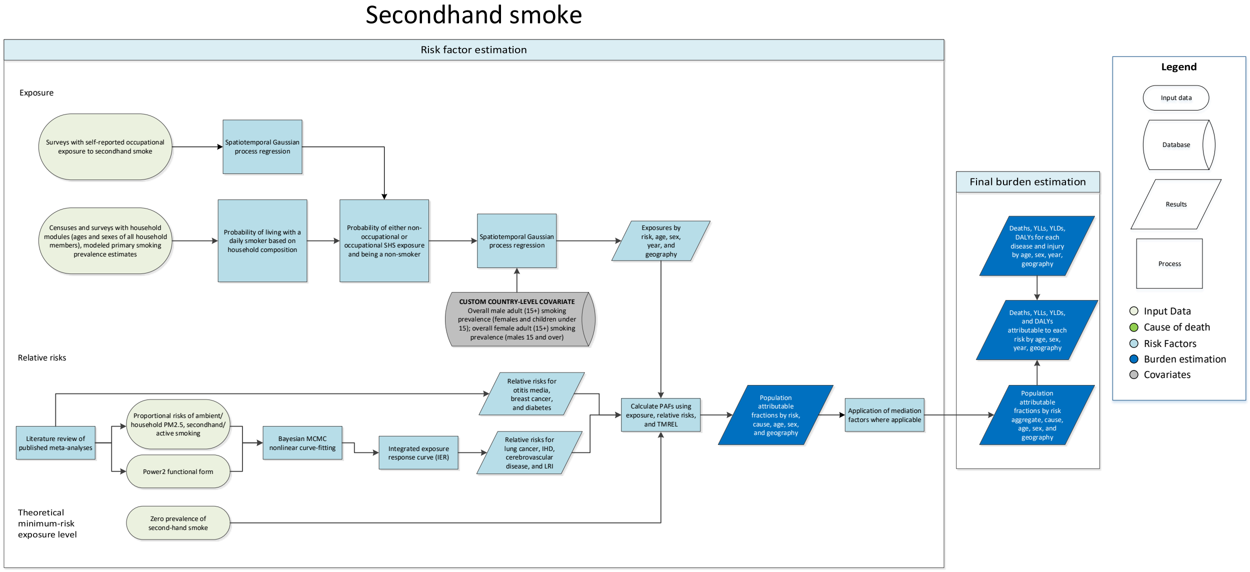

Exposure

Case definition

We define secondhand smoke exposure as current exposure to secondhand tobacco smoke at home, at work, or in other public places. We use household composition as a proxy for non-occupational secondhand smoke exposure and make the assumption that all persons living with a daily smoker are exposed to tobacco smoke. We use surveys to estimate the proportion of individuals exposed to secondhand smoke at work. We only consider non-smokers to be exposed to secondhand smoke. Non-smokers are defined as all persons who are not daily smokers. Ex-smokers and occasional smokers are considered non-smokers in this analysis. Exposure is evaluated for both children and adults.

Input data

To calculate the proportion of non-smokers who live with at least one smoker, we used unit record data on household composition, which included the ages and sexes of all persons living in the same household. Our sources included representative major survey series with a household composition module, including the Demographic Health Surveys (DHS), the Multiple Indicator Cluster Surveys (MICS), and the Living Standards Measurement Surveys (LSMS); and national and subnational censuses, which included those captured in the IPUMS project and identified using the Global Health Data Exchange catalog (GHDx).

To calculate the proportion of individuals exposed to secondhand smoke at work, by age and sex, we used cross-sectional surveys that ask respondents about self-reported occupational secondhand smoke exposure. Sources include the Global Adult Tobacco Surveys, Eurobarometer Surveys, and WHO STEPS Surveys. We identified sources using the GHDx.

Estimates of primary smoking prevalence in each location were also used in our calculations. Further details on the estimation of primary smoking prevalence can be found in the Smoking methods appendix.

Modelling strategy

We estimated the probability that each person is living with a smoker and is also a non-smoker themselves using set theory. First, household composition data were used at the individual level to capture the ages and sexes of each person in the household. Second, we analyzed surveys with both household composition data and tobacco use questions and determined that the distribution of household size, mean age of the household members, and the age distribution were not significantly different between households with and without a self-reported smoker. Since we did not find that household composition varied between smokers and non-smokers, we then used the GBD 2017 primary smoking prevalence model to calculate the probability that each household member is a smoker. Next, we used the probability of the union of sets on each individual household member to calculate the overall probability that at least one of the other household members was a smoker. We incorporated occupational exposure by modelling prevalence of current exposure to secondhand smoke at work, by age, sex, location, and year, using ST-GPR. In order to avoid double counting we calculated the probability that an individual is exposed through either non-occupational exposure or occupational exposure, given their age, sex, and household composition. Finally, we multiplied this probability of exposure by the probability that the individual is not a smoker themselves (i.e. 1 minus primary smoking prevalence for that person's location, year, age, and sex). We then collapse these individual-level probabilities to produce average probabilities of exposure by location, year, age, and sex.

These probabilities were modelled in the GBD ST-GPR framework, which generates exposure estimates from a mixed effects hierarchical linear model plus weighted residuals smoothed across time, space, and age. The linear model formula was fit separately by sex using restricted maximum likelihood in R.

We used the sex-specific overall smoking prevalence for adults (age 15 and older) as a country-level covariate in the model. The overall male adult daily smoking prevalence was used as the covariate for females of all ages and for males under age 15. The overall female adult daily smoking prevalence was used as the covariate for males age 15 and older. This was a modelling change from GBD 2015, in which we used the male age-standardised smoking prevalence for the adult female and children under 15 model, and the female age-standardised smoking prevalence for the adult male model.

All input data points from the probability calculation had a measure of uncertainty (variance and sample size) coming from the uncertainty of the primary smoking prevalence model and the sample size from the unit record data going into the modelling process. Geographic r*andom effects were used in model fitting but were not used in prediction.*

Theoretical minimum-risk exposure level

The theoretical minimum-risk exposure level for secondhand smoke is zero exposure among non-smokers, meaning that non-smokers would not live with any primary smokers.

Relative risks

For children ages 0-14, we estimated the burden of otitis media attributable to secondhand smoke exposure. For all ages we estimated the burden of lower respiratory infections (LRI), and for adults

greater or equal to 25 years of age we estimated the burden of lung cancer, chronic obstructive pulmonary disease (COPD), ischemic heart disease, and cerebrovascular disease attributable to secondhand smoke exposure, breast cancer, and type-II diabetes.

For lung cancer, ischemic heart disease, cerebrovascular disease, and LRI, we used country-specific relative risks created using integrated exposure response curves (IER) for PM2.5 air pollution. The relative risks for otitis media, breast cancer, and diabetes are derived from published meta-analyses.

We used the standard GBD population attributable fraction (PAF) equation to estimate burden based on exposure and relative risks.

Ambient Ozone Pollution Capstone Appendix

Flowchart

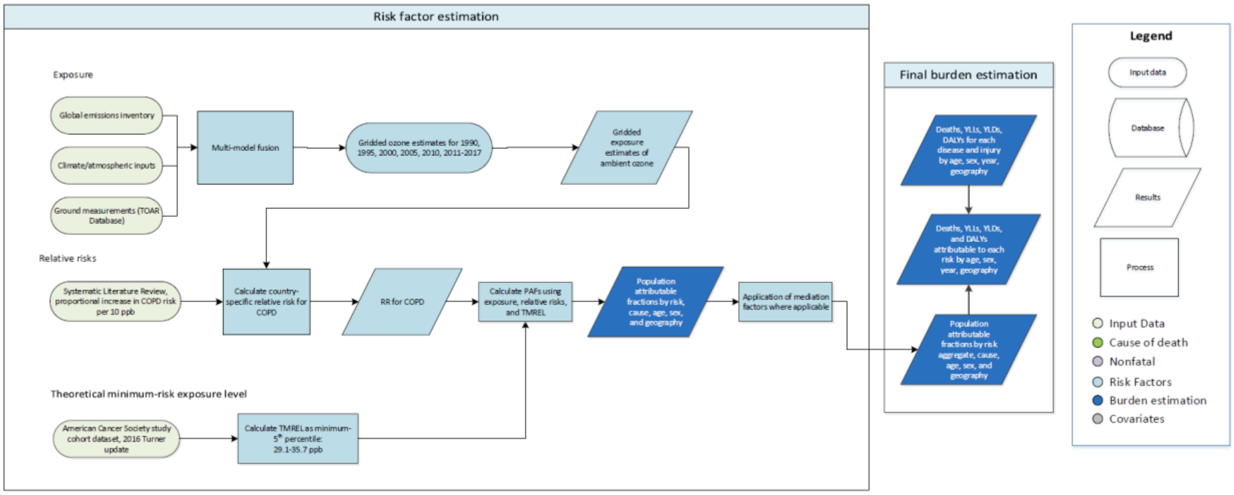

Input data and methodological summary

Exposure

Case definition

For GBD 2017, exposure to ozone pollution is defined as the seasonal (6 month period with highest mean) 8 hour daily maximum ozone concentrations, measured in ppb. This was an update from the previous exposure metric in accordance with an update of the American Cancer Society Cancer Prevention Study II (ACS CPS-II).[1]

Input data

Previously, exposure estimates were based on a chemical transport model with no measurement database or evaluation. In GBD 2017, exposure estimates incorporated a new comprehensive ozone measurement database (TOAR).[2] This enabled a continent-specific weighted blend of 6 chemical transport models with grid cell level bias correction. The use of ground measurements also enabled the incorporation of error estimation, where previously we had assumed a +/- 6% error. The output of this model is a global raster of ozone exposure which is a summary for the years 2008-2014.[3]

Modelling strategy for trends

To estimate ozone concentrations over time, we used the trend from the former GBD model for 1990, 2000, and 2010 and cubic splines for 1995, 2005, and 2011, after applying an adjustment for the difference in trends between the previous (1 hour daily maximum) and current (8 hour daily maximum metrics. Annualised rate of change was used to predict for the years 2012-2017.

Theoretical minimum-risk exposure level

The TMREL of ozone was updated this year based on the exposure distribution from the updated ACS CPS-II study.[1] A uniform distribution was drawn around the minimum and 5th percentile values experienced by the cohort, defined as ~U(29.1, 35.7), in ppb.

Relative risks

Since the inclusion of ozone in GBD 2010 the relative risk of ozone exposure for respiratory COPD mortality has been defined to be 1.029, 95% C.I. (1.01-1.048) per 10 ppb of ozone exposure. Note that this comes from one study that looked at all respiratory mortality.[4] For GBD 2017, we performed a literature review and included five cohorts from Canada, the UK, and the US which all measured COPD mortality. For cohorts with multiple analyses we chose the most recent analysis. We found a resulting relative risk of 1.06, 95% C.I. (1.02, 1.10).

References

1. Turner MC,Jerrett M, Pope CA 3rd, Krewski D, Gapstur SM, Diver WR, Beckerman BS, Marshall JD, Su J, Crouse DL, Burnett RT. Long-term ozone exposure and mortality in a large prospective study. *Am J Respir Crit Care Med*. 2016; 193(10): 1134-42.
2. Schultz MG, Schröder S, Lyapina O, Cooper O, Galbally I, Petropavlovskikh I, et al.. Tropospheric Ozone Assessment Report: Database and Metrics Data of Global Surface Ozone Observations. Elem Sci Anth. 2017;5:58. DOI: http://doi.org/10.1525/elementa.244
3. Chang K-L, Cooper OR. A new method for combining observations and multiple model output for an improved estimate of the global surface ozone distribution. Atmospheric Chemistry and Physics Discussion, 2018.
4. Jerrett M, Burnett RT, Pope CA, et al. Long-term ozone exposure and mortality. N Engl J Med 2009; 360: 1085–95.

Occupational Risk Factors Capstone Appendix

Exposure definitions

The following definitions were used for occupational risk factor exposures. All exposures were estimated for ages 15 and older.

Occupational Asbestos	Cumulative lifetime exposure to occupational asbestos, using mesothelioma death rate as an analogue
Occupational Asthmagens	Proportion of the working population exposed to asthmagens, based on population distributions across nine occupational categories
Occupational Carcinogens (arsenic, benzene, beryllium, cadmium, chromium, diesel engine exhaust, formaldehyde, nickel, polycyclic aromatic hydrocarbons, silica, sulfuric acid, and trichloroethylene)	Proportion of the population that was ever occupationally exposed to carcinogens at high or low exposure levels, based on population distributions across seventeen economic activities
Occupational Ergonomic Factors	Proportion of the working population exposed to low back pain-inducing work, based on population distributions across nine occupational categories
Occupational Injuries	Proportion of injuries in the working-age population attributable to occupational work, based on fatal injury rates in seventeen economic activities
Occupational Noise	Proportion of the population occupationally exposed to 85+ decibels of noise, based on population distributions across seventeen economic activities
Occupational Particulates	Proportion of the population occupationally exposed to particulates, based on population distributions across seventeen economic activities

Economic activities and occupations were coded according to the following categories:

Economic Activities	Occupations
Agriculture, hunting, forestry	Legislators, senior officials, and managers
Fishing	Professionals
Mining and Quarrying	Technicians and associate professionals
Manufacturing	Clerks

Electricity, gas, and water	Service workers and shop/market sales workers
Construction	Skilled agricultural and fishery workers
Wholesale and retail trade/repair	Plant and machine operators and assemblers
Hospitality	Craft and related workers
Transport, storage, and communication	Elementary occupations
Financial intermediation	
Real estate/renting	
Public administration/defense; compulsory social security	
Education	
Health and social work	
Other community/social/personal service activities	
Private households	
Extra-territorial organisations/bodies	

Input data

Primary inputs were obtained from the ILO,[1-4] and included raw data on economic activity proportions, occupation proportions, fatal injury rates, and employment to population ratio estimates. A systematic web review was conducted in order to collect the underlying microdata from the ILO's estimates to aid in re-extraction at greater levels of granularity. Where freely available, survey datasets were downloaded from the survey organisations in question. Other datasets were obtained through submission of requests to agencies and through the GBD collaborator network. Microdata was tabulated in order to create survey-weighted estimates of economic activities and occupations for the GBD geographies and years. Various classification systems were crosswalked to ISIC Rev.3 (for economic activities) and ISCO 1988 (for occupations). Subnational estimates for UK and China were added to the datasets for economic activities and occupations.[5,6]

For occupational asbestos, primary inputs were obtained through GBD 2017 cause of death estimates and published studies.[7,13,14]

Uncertainty for inputs where microdata was unavailable was generated by fitting a Loess curve to the data and determining the standard deviation of the data from the fitted curve.

Modelling strategies

A Spatio-temporal Gaussian process regression (ST-GPR) was used to generate estimates for all years and locations for the primary inputs. Study level covariates used in the prior model were education in

years per capita, geological covariates (for mining models), the proportion of the population living with access to a coastline (for fishing models), the IHME socio-demographic index (SDI), the mean temperature/latitude (for agriculture models), and the proportion of the population living in urban areas. Space-time parameters were chosen by maximising out-of-sample cross-validation and minimising RMSE. For economic activity and occupation proportions, estimates from ST-GPR were then re-scaled to sum to 1 across categories by dividing each estimate by the sum of all the estimates.

The following sections describe the modelling approaches for each occupational risk's exposure prevalence.

Occupational carcinogens, occupational noise, and occupational particulates

Prevalence of exposure to these risks was determined using the following equation:

$$Prevalence\ of\ Exposure_{c,y,s,a,r,l} = \sum_{EA} Proportion_{EA,c,y} * EAP_{c,y,s,a} * Exposure\ rate_{EA,r,l,d}$$

where:

EAP = economically active population	c = country	r = risk
EA = economic activity	d = duration	s = sex
a = age	l = level of exposure	y =year

Exposure rate was provided by expert group recommendations and literature[8-11] (see table 1). The CAREX database was used in order to quantify the association between exposure by industry/carcinogen to SDI across all the countries in the database. This effect was used to predict exposure in countries that were not included in CAREX. Duration was considered for occupational carcinogens through application of occupational turnover factors[12] and for occupational noise and particulates by calculating cumulative exposure as the average exposure over the lifetime (the past 50 years) for each age/sex cohort.

Occupational ergonomic factors and occupational asthmagens

Prevalence of exposure to these risks was determined using the following equation:

$$Prevalence\ of\ Exposure_{c,y,s,a,r} = \sum_{EA} Proportion_{OCC,c,y} * EAP_{c,y,s,a}$$

where:

EAP = economically active population	c = country	r = risk
OCC = occupation	a = age	s = sex
		y = year

Occupational injuries

Occupational injury counts were estimated using the following equation:

$$Occupational\ fatal\ injuries_{c,y,a,s} = \sum_{EA} Injury\ rate_{EA,c,y,s} * Population_{c,y,a,s} * EAP_{c,y,s,a} * Proportion_{EA,c,y}$$

where:

EAP = economically active population | c = country | y = year

EA = economic activity | a = age | s = sex

Occupational asbestos

Prevalence of exposure to asbestos was estimated using the asbestos impact ratio (AIR), which is equivalent to the excess deaths due to mesothelioma observed in a population divided by excess deaths due to mesothelioma in a population heavily exposed to asbestos. Formally, this is defined using the following equation:

$$AIR = \frac{Mort_{c,y,s} - N_{c,y,s}}{Mort^{*}_{c,ys,} - N_{c,y,s}}$$

where:

Mort = Mortality rate due to mesothelioma

Mort* = Mortality rate due to mesothelioma in population highly exposed to asbestos

N = Mortality rate due to mesothelioma in population not exposed to asbestos

c = country

y = year

s = sex

Mortality rate due to mesothelioma was estimated from GBD 2017 causes of death.[7] Mortality rate due to mesothelioma in populations not exposed to asbestos was calculated using the model in Lin et al.,[13] while the mortality rate due to high exposure to asbestos was estimated in Goodman et al.[14] Asbestos exposure prevalence created using the AIR was used to estimate PAFs for all asbestos-associated causes except for mesothelioma. Custom PAFs were calculated for mesothelioma by using the ratio of the excess mortality with respect to an unexposed population (Mort – N) divided by the mortality rate in the population in question (Mort). This calculation assumes that all mesothelioma is a product of occupational asbestos exposure and could potentially over-estimate burden due to occupational asbestos exposure in populations with high non-occupational asbestos exposure.

Theoretical minimum-risk exposure level

For all occupational risks, with the exception of occupational asbestos, the theoretical minimum-risk exposure level was assumed to be no exposure to that risk.

Relative risk

Relative risks were obtained for all occupational risks by conducting a systematic review of published meta-analysis. The estimates used, as well as the associated studies, are reported by category group in appendix table 5.

PAFs

For all occupational risks, with the exception of injuries (outlined below) and mesothelioma (outlined above), PAFs were calculated using the prevalences estimated above, using the PAF formula in outlined in the GBD 2017 methods appendix.

Occupational injuries PAF

The PAFs for occupational injuries were calculated using the following formula:

$$PAF_{c,y,a,s} = \frac{Occupational\ fatal\ injuries_{c,y,a,s} - TMREL}{Fatal\ injuries_{c,y,a,s}}$$

where:

c = country	a = age
y = year	s = sex

Fatal injury totals were obtained from GBD 2017 causes of death.[7]

References

1. International Labour Organization (ILO). International Labour Organization Database (ILOSTAT) - Employment by Sex and Economic Activity. International Labour Organization (ILO).

2. International Labour Organization (ILO). International Labour Organization Database (ILOSTAT) - Employment by Sex and Occupation. International Labour Organization (ILO).

3. International Labour Organization (ILO). International Labour Organization Database (ILOSTAT) - Fatal Injuries by Sex and Economic Activity. International Labour Organization (ILO).

4. International Labour Organization (ILO). International Labour Organization LABORSTA Economically Active Population, Estimates and Projections, October 2011. International Labour Organization (ILO), 2011.

5. Office for National Statistics (United Kingdom). Nomis Official Labor Market Statistics - Annual Population Survey. Newport, United Kingdom: Office for National Statistics (United Kingdom).

6. National Bureau of Statistics of China. China 1% National Population Sample Survey 1995. Ann Arbor, United States: China Data Center, University of Michigan.

7. GBD 2017 Mortality and Causes of Death Collaborators. Global, regional, and national life expectancy, all-cause and cause-specific mortality for 249 causes of death, 1980–2017: a systematic analysis for the Global Burden of Disease Study 2017. *Lancet Rev*.

8. Wilson DH, Walsh PG, Sanchez L, *et al.* The epidemiology of hearing impairment in an Australian adult population. *Int J Epidemiol* 1999; 28: 247–52

9. Kauppinen T, Toikkanen J, Pederson D, Young R, Kogevinas M, Ahrens W, et al. Occupational Exposure to Carcinogens in the European Union in 1990-93. Helsinki, Finland: Finnish Institute of Occupational Health; 1998.

10. Kauppinen T, Toikkanen J, Pedersen D, Young R, Ahrens W, Boffetta P, et al. Occupational exposure to carcinogens in the European Union. *Occup Environ Med* 2000; 57(1): 10–18.

11. Driscoll T, et al. The global burden of non-malignant respiratory disease due to occupational airborne exposures. *American Journal of Industrial Medicine 2005; 48*(6): 432-445.

12. Nelson, D. I., Concha-Barrientos, M., Driscoll, T., Steenland, K., Fingerhut, M., Punnett, L. & Corvalan, C. (2005). The global burden of selected occupational diseases and injury risks: Methodology and summary. *American journal of industrial medicine*, *48*(6), 400-418

13. Lin R-T, Takahashi K, Karjalainen A, *et al.* Ecological association between asbestos-related diseases and historical asbestos consumption: an international analysis. *Lancet* 2007; **369**: 844–9.

14. Goodman M, Morgan RW, Ray R, Malloy CD, Zhao K. Cancer in asbestos-exposed occupational cohorts: a meta-analysis. *Cancer Causes Control* 1999; **10**: 453–65.

Ambient Particulate Matter Pollution Capstone Appendix

Flowchart

Ambient particulate matter pollution

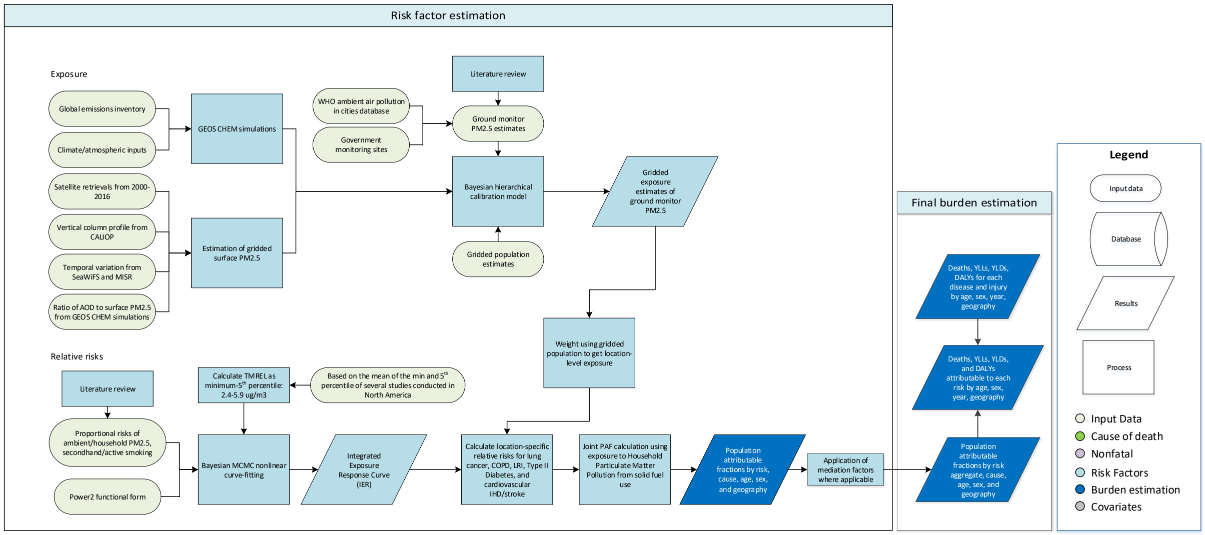

Input data and modeling strategy

Exposure

Definition

Exposure to ambient air pollution is defined as the population-weighted annual average mass concentration of particles with an aerodynamic diameter less than 2.5 micrometers ($PM_{2.5}$) in a cubic meter of air. This measurement is reported in µg/m^3.

Input Data

The data used to estimate exposure to ambient air pollution is drawn from multiple sources, including satellite observations of aerosols in the atmosphere, ground measurements, chemical transport model simulations, population estimates, and land-use data.

The following details the updates in methodology and input data used in GBD 2017.

$PM_{2.5}$ ground measurement database

Updates of ground measurements used for GBD 2017 include using more recent data than that used previously and the addition of data from new locations. The data from the 2018 update of the WHO Global Ambient Air Quality Database include monitor-specific measurements of concentrations of PM_{10} and $PM_{2.5}$ from 9,960 ground monitors (up from 6,003 in GBD 2016) from 108 countries. The majority of measurements were recorded in 2016 (as there is a lag in reporting measurements, little data from 2017 were available). Annual averages were excluded if they were based on less than 75% coverage within a year. Collection year ranged from 2008 to 2017 in data used. If information on coverage was not available then data were included unless they were already sufficient data within a country (monitor density greater than 0.1).

For locations measuring only PM_{10}, $PM_{2.5}$ measurements were estimated from PM_{10}. This was performed using a hierarchy of conversion factors ($PM_{2.5}/PM_{10}$ ratios): (i) for any location a 'local' conversation factor was used, constructed as the ratio of the average measurements (of $PM_{2.5}$ and PM_{10}) from within 50km and within the same country, if such were available' (ii) if there was not sufficient local information to construct a conversion factor then a country-wide conversion factor was used; and (iii) if there was no appropriate information within a country then a regional factor was used. In each case, to avoid the possible effects of outliers in the measured data (both $PM_{2.5}$ and PM_{10}), extreme values of the ratios were excluded (defined as being greater/lesser than the 95 and 5% quantiles of the empirical distributions of conversion factors) of the latter two cases for the country measurements were available, for both metrics. As in the GBD 2013 and GBD 2015/GBD 2016 databases, in addition to values of $PM_{2.5}$ and whether they were direct measurement or converted from PM_{10}, the database also included additional information, where available, related to the ground measurements such as monitor geo coordinates and monitor site type.

Satellite-based estimates

The updated satellite-based estimates for years 1998-2016 are described in detail in van Donkelaar et al. 2016.[0] These estimates were available at 0.1°×0.1° resolution (~11 x 11 km resolution at the equator) and combine aerosol optical depth retrievals from multiple satellites with the GEOS Chem chemical transport model and land use information.

Population data

A comprehensive set of population data on a high-resolution grid was obtained from the Gridded Population of the World (GPW) database. These estimates are adjusted to match UN2015 Population Prosepectus. These data are provided on a 0.0417°×0.0417° resolution. Aggregation to each 0.1°×0.1° grid cell comprised of summing the central 3 × 3 population cells. As this resulted in a resolution higher than necessary, it was repeated four times, each offset by one cell in a North, South, East and West direction. The average of the resulting five quantities was used as the estimated population for each grid cell. Population estimates for 2000, 2005, 2010, 2015 and 2020 were available from GPW version 4 revision 10. Populations for 2016 and 2017 were obtained by interpolation using natural splines with knots placed at 2000, 2005, 2010, 2015 and 2020. This was performed for each grid cell.

Chemical transport model simulations

Estimates of the sum of particulate sulfate, nitrate, ammonium and organic carbon and the compositional concentrations of mineral dust simulated using the GEOS Chem chemical transport model, and a measure combining elevation and the distance to the nearest urban land surface (as described in van Donkelaar et al. 2016[0]) were available for 2000 to 2016 for each 0.1°×0.1° grid cell. These were not included within the GBD 2013 analysis.

Modelling strategy

Significant advances have been made in the methodology used to estimate exposure to ambient particulate matter pollution since GBD 2013. The following is a summary of the modelling approach, known as the Data Integration Model for Air Quality (DIMAQ) used in GBD 2015, 2016, and 2017; further details can be found in Shaddick *et al.* (2017).[2]

In GBD 2010 and GBD 2013 exposure estimates were obtained using a single global function to calibrate available ground measurements to a 'fused' estimate of $PM_{2.5}$; the mean of satellite-based estimates and those from the TM5 chemical transport model, calculated for each 0.1°×0.1° grid cell. This was recognised to represent a trade-off between accuracy and computationally efficiency when utilising all the available data sources. In particular, the GBD 2013 exposure estimates were known to underestimate ground measurements in specific locations (see discussion in Brauer et al., 2013[3]). This underestimation was largely due to the use of a single, global, calibration function, whereas in reality the relationship between ground measurements and other variables will vary spatially.

In GBD 2015 and GBD 2016, coefficients in the calibration model were estimated for each country. Where data were insufficient within a country, information can be `borrowed' from a higher aggregation (region) and if enough information is still not available from an even higher level (super-region). Individual country level estimates were therefore based on a combination of information from the country, its region and super-region. This was implemented within a Bayesian Hierarchical modelling (BHM) framework. BHMs provide an extremely useful and flexible framework in which to model complex relationships and dependencies in data. Uncertainty can also be propagated through the model allowing uncertainty arising from different components, both data sources and models, to be incorporated within estimates of uncertainty associated with the final estimates. The results of the modelling comprise a posterior distribution for each grid cell, rather than just a single point estimate, allowing a variety of summaries to be calculated. The primary outputs here are the median and 95% credible intervals for each grid cell. Based on the availability of ground measurement data, modelling and evaluation was focused on the year 2016.

The GBD 2017 model was updated to also include within country calibration variation. [4] The model used for GBD2017, henceforth referred to as DIMAQ2, provides a number of substantial improvements over the initial formulation of DIMAQ. In DIMAQ, ground measurements from different years were all assumed to have been made in the primary year of interest (i.e. 2014 for GBD2015 before extrapolation) and then regressed against values from other inputs (e.g. satellites etc.) made in that year. In the presence of changes over time therefore, and particularly in areas where no recent measurements were available, there was the possibility of mismatches between the ground measurements and other variables. In DIMAQ2, ground measurements and matched with other inputs (over time) and the possibility of the (global level) coefficients being allowed to vary over time, subject to smoothing that is induced by a second-order random walk process. In addition, the manner in which spatial variation can be incorporated within the model has developed: where there is sufficient data, the calibration equations can now vary (smoothly) both within and between countries, achieved by allowing the coefficients to follow (smooth) Gaussian processes. Where there is insufficient data within a country, to produce accurate equations, as before information is borrowed from lower down the hierarchy and it is supplemented with information from the wider region.

DIMAQ2 is used for all regions except for the North Africa-Middle East and Sub-Saharan super-regions and remote islands where there is insufficient data to allow the extra complexities of the new model to be implemented. In the North Africa-Middle East and Sub-Saharan super-regions a simplified version of DIMAQ2 is used in which the temporal component is dropped, and for remote islands the original DIMAQ is used.

Due to both the complexity of the models and the size of the data, notably the number of spatial predictions that are required, recently developed techniques that perform 'approximate' Bayesian inference based on integrated nested Laplace approximations (INLA) were used.[5] Computation was performed using the R interface to the INLA computational engine (R-INLA). Fitting the models and performing predictions for each of the ca. 1.4 million grid cells required the use of a high performance computing cluster (HPC) making use of high memory nodes.

Model evaluation

Model development and comparison was performed using within- and out-of-sample assessment. In the evaluation, cross validation was performed using 25 combinations of training (80%) and validation (20%) datasets. Validation sets were obtained by taking a stratified random sample, using sampling probabilities based on the cross-tabulation of $PM_{2.5}$ categories (0-24.9, 25-49.9, 50-74.9, 75-99.9, 100+ μg/m^3) and super-regions, resulting in them having the same distribution of $PM_{2.5}$ concentrations and super-regions as the overall set of sites. The following metrics were calculated for each training/evaluation set combination: for model fit - R^2 and deviance information criteria (DIC, a measure of model fit for Bayesian models); for predictive accuracy - root mean squared error (RMSE) and population weighted root mean squared error (PwRMSE).

All modelling was performed on the log-scale. The choice of which variables were included in the model was made based on their contribution to model fit and predictive ability. The following is a list variables and model structures that were included in DIMAQ.

Continuous explanatory variables:

- (SAT) Estimate of $PM_{2.5}$ (in μgm^{-3}) from satellite remote sensing on the log-scale.
- (POP) Estimate of population for the same year as SAT on the log-scale.
- (SNAOC) Estimate of the sum of sulfate, nitrate, ammonium and organic carbon simulated using the GEOS Chem chemical transport model.
- (DST) Estimate of compositional concentrations of mineral dust simulated using the GEOS Chem chemical transport model.
- (EDxDU) The log of the elevation difference between the elevation at the ground measurement location and the mean elevation within the GEOS Chem simulation grid cell multiplied by the inverse distance to the nearest urban land surface.

Discrete explanatory variables:

- (LOC) Binary variable indicating whether exact location of ground measurement is known.
- (TYPE) Binary variable indicating whether exact type of ground monitor is known.
- (CONV) Binary variable indicating whether ground measurement is $PM_{2.5}$ or converted from PM_{10}.

Random Effects:

- Grid cell random effects on the intercept to allow for multiple ground monitors in a grid cell.
- Country-region-super-region hierarchical random effects for the intercept.
- Country-region-super-region hierarchical random effects for the coefficient associated with SAT .
- Country-region-super-region hierarchical random effects for the coefficient associated with the difference between estimates from CTM and SAT.
- Country-region-super-region hierarchical random effects for the coefficient associated with POP.
- Country level random effects for population uses a neighbourhood structure allowing specific borrowing of information from neighbouring countries.
- Within a region, country level effects of SAT and the difference between SAT AND CTM are assumed to be independent and identically distributed.
- Within a super-region, region level random effects are assumed to be independent and identically distributed.
- Super-region random effects are assumed to be independent and identically distributed.

Interactions*:*

- Interactions between the binary variables and the effects of SAT and CTM.

In addition, DIMAQ2 includes

- Smoothed, spatially varying, random-effects for the intercept
- Smoothed, spatially varying, random-effects for the coefficient of coefficient associated with SAT
- Smoothed, temporally varying, random-effect for the intercept

Results

The final model contained the following variables: SAT, POP, SNAOC, DST, EDxDU, LOC, TYPE, and CONV, together with interactions between SAT and each of LOC, TYPE and CONV. The model structure contained grid cell random effects on the intercept to allow for multiple ground monitors in a grid cell, country-region-super-region hierarchical random effects for intercepts and SAT and country level random effects for population using a neighbourhood structure allowing specific borrowing of information from neighbouring countries together with region-super-region hierarchical random effects for POP. Notably, and as in GBD 2015 and GBD 2016, based on the evaluation of candidate models, including estimates from the TM5 chemical transport model (CTM) used in GBD 2013 did not improve the predictive ability of the model and was therefore not included.

Compared to the model used in GBD2013, DIMAQ showed improved predictions of ground measurements in all super regions with improvements in both within-sample fit; with a global population-weighted RMSE of 12.1 $\mu g/m^3$ compared to 23.1 $\mu g/m^3$ when using the GBD 2013 approach.[0] Using the larger database available for GBD2017, with potentially more variability in measurements, DIMAQ2 shows an additional improvement on DIMAQ: overall population-weighted RMSE reduced from 9.32 to 8.11 (12.12 to 11.17 when using all data, irrespective of within-year coverage). Reductions by super-region can be seen in Figure 1. Reductions can be seen in all super-regions with particular improvement in the Southeast Asia, East Asia and Oceania super-region which is based largely on a substantial increase in accuracy in China, PwRMSE 6 vs 9 $\mu g/m^3$

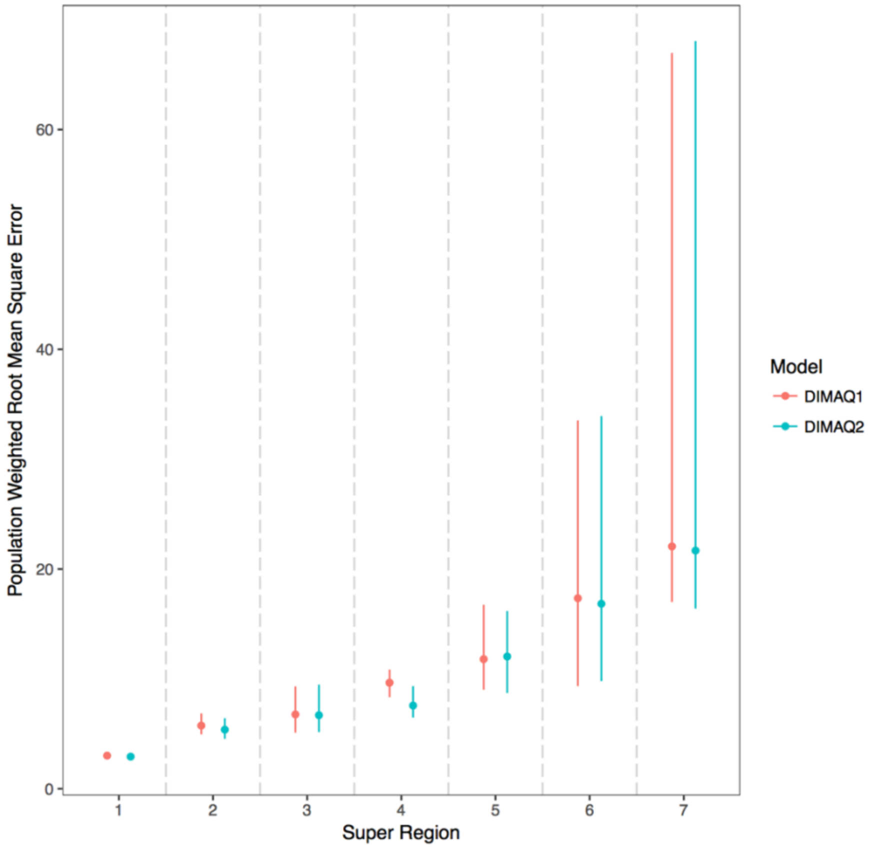

Figure 1: Summary measures of predictive ability, globally and by super-region. Dots denote the median values of population weighted root mean squared error (μg/m^3) from 25 validation sets with vertical lines showing the range of values over those sets.

Estimates for other years

In contrast to the method used previously, where estimates (of $PM_{2.5}$) were extrapolated to produce estimates for the year of interest (e.g. 2017 where data was available up to and including 2016) due to the extra complexity of the smooth spatial processes in DIMAQ2 this would not be possible in any straightforward manner. With DIMAQ2 it is the input variables that are extrapolated; this allows estimates for 2017 to be produced in the same way as other years and crucially, allows measures of uncertainty to be produced within the BHM framework rather than by using post-hoc approximations.

Satellite estimates and quantities estimated using the GEOS-Chem model were available for 1990, 1995, 2000, 2005, 2010-2016. Estimates of these input variables for 2017 were produced by extrapolating, on a cell-by-cell basis, using natural splines. Population estimates for 2000, 2005, 2010, 2015 and 2020 were availalble from GPW version 4. For 1990 and 1995 data were extracted from GPW version 3, as in GBD2013.[2] As with populations for 2015, values for each cell for 2011-2017 were obtained by interpolation using natural splines with knots placed at 2000, 2005, 2010, 2015 and 2020.

These were used as inputs to DIMAQ, enabling estimates of exposures to be obtained for each of these years respectively. For 2017, estimates of exposures were obtained from predictions from locally-varying regression models.[6] For each cell a model was fit to the values within that cell over time, with a constraint placed on the rate of change between 2016 and 2017 to avoid unrealistic and/or unjustified extrapolation of trends. Measures of uncertainty were obtained by repeating the procedure for the limits of the 95% credible intervals, again on a cell-by-cell basis.

Population-weighted exposure generation

To generate a distribution of the population-weighted ambient particulate matter, we took a weighted sampling strategy, taking samples from all grid cells in a given location. For example, for a country with n grid cells, we randomly sampled 1000 values from the n (grid cells) x 1000 (samples) where the probability of being sampled was proportional to the population of that grid cell.

Theoretical minimum-risk exposure level

The TMREL was assigned a uniform distribution with lower/upper bounds given by the average of the minimum and 5th percentiles of outdoor air pollution cohort studies exposure distributions conducted in North America, with the assumption that current evidence was insufficient to precisely characterise the shape of the concentration-response function below the 5th percentile of the exposure distributions. The TMREL was defined as a uniform distribution rather than a fixed value in order to represent the uncertainty regarding the level at which the scientific evidence was consistent with adverse effects of exposure. The specific outdoor air pollution cohort studies selected for this averaging were based on the criteria that their 5th percentiles were less than that of the American Cancer Society Cancer Prevention II (CPSII) cohort's 5th percentile of 8.2 based on Turner et al. (2016).[7] This criterion was selected since GBD 2010 used the minimum, 5.8, and 5th percentile solely from the CPS II cohort. The resulting lower/upper bounds of the distribution for GBD 2017 were 2.4 and 5.9. This has not changed since GBD 2015.

Relative risks and population attributable fractions

We estimated the Ambient Air Pollution-attributable burden of disease based on the relation of long-term exposure to PM2.5 with Ischemic Heart Disease, stroke (ischemic and hemorrhagic), COPD, lung cancer and acute lower respiratory infection. These were also the pollutant-outcome pairs used to estimate the Ambient Air Pollution attributable burden since GBD 2010. For GBD 2017 we also added Type II Diabetes as an outcome of ambient air pollution. We used results from all cohort studies published as of July 2018 that reported cause-specific relative risk estimates based on measured or modelled PM2.5 and that adjusted for potential confounding due to other major risk factors such as tobacco smoking using data for each study participant.

Bowe et al. recently published work that assembled the evidence for the relationship between particulate matter and diabetes to generate IER curves and attributable burden estimates based on methodologies similar to those of the GBD. [8]

When generating the IER for Type II Diabetes, we included all eight of the studies summarized by Bowe et al. in addition to six other cohorts. Resulting attributable burden estimates were remarkably similar to GBD 2017 results. All citations for studies used in the fitting of the IER curve can be found using the GBD 17 Data Input Sources Tool.

Integrated exposure response function

The Integrated Exposure Response Function (IER) was created to ascertain the shape of the dose response curve for a variety of health outcomes across a wide range of exposure to PM2.5. The IER model is fit by integrating RR information from studies of outdoor air pollution (OAP), Second hand tobacco smoke (SHS), Household Air Pollution (HAP), and Active Smoking (AS). Because OAP studies are often performed at the lower end of the ambient air pollution range, incorporating other exposures to particulate matter enables RR estimation across the global range of exposure. These methods have been described in detail elsewhere.[9,10]

Notable changes for GBD 2017 include added studies for OAP, SHS, and HAP, updated literature reviews for AS studies, and more informative priors to stabilize the shape of the IER curves.

- We added all newly published cohorts of long-term exposure to Ambient PM2.5 and incidence or mortality due to IHD, stroke, COPD, lung cancer, and LRI. One notable addition was the China Male Cohort which included mortality due to IHD, Stroke, COPD, Lung Cancer, and Diabetes (unpublished analysis).[11] This study represented a higher exposure range than most of our previously incorporated studies with 5th and 95th percentile of 15.5 and 77.1 micrograms/m^3. For Type II Diabetes, the new outcome included in GBD 2017, we included all cohorts which measured long-term PM2.5 exposure and incident diabetes or mortality due to diabetes.
- We did not change the SHS input studies with the exception of including all studies from a recent meta-analysis examining the relationship between SHS and Type II Diabetes.[12] We also added seven studies found from a systematic review examining SHS exposure and COPD. We had previously not included SHS in the formation of this curve.
- We added four cohort studies of HAP and any of our measured outcomes. Previously we have only included which measured levels of PM2.5 exposure. To incorporate cohort studies with binary exposure data (presence or absence of solid-fuel use for cooking) we used the PM2.5 mapping function (see Household Air Pollution Appendix for more details) to obtain a PM2.5 level attributed to solid fuel use for cooking for the location-year of the study (Exp_{HAP}). We also

used the OAP exposure model to obtain an OAP PM2.5 level for the location-year (Exp_{OAP}). The study RR was used to inform the curve on the range of Exp_{OAP} to (Exp_{OAP} + Exp_{HAP}).

- For all outcomes, we used updated systematic reviews of the literature performed by the GBD smoking team for studies examining cigarettes smoked per day and the six IER outcomes to inform the high exposure range of the curve. The smoking team found that the process of systematic review and inclusion of all acceptable studies led to lower relative risks.
- To help obtain more reasonable curve fits, we added more informative priors to two of three IER function parameters in the MCMC Bayesian fitting process.

Limitations

It is important to recognize the inherent limitations of the IER approach. The use of various sources to construct a risk curve assumes an equitoxicity of particles, consistent with evaluations by US EPA and WHO. However, current evidence suggests there are differences in health impact by source, size, and chemical composition. This is seen when comparing studies of ambient and household particulate matter. As this body of evidence grows, we will continue to re-examine our strategy for the integrated exposure-response curve. For now, the IER is a practical solution to fill gaps in the literature where we do not have sufficient evidence such as household air pollution exposures and ambient in highly polluted areas.

Additionally, currently the exposure concentrations used for both SHS and AS data points when fitting the IER are contrasted with the TMREL and do not take into account ambient particulate matter pollution. In future iterations of fitting the curve, we will test alternate approaches, including a similar approach to HAP, allowing each data point to inform the curve on the range of Exp_{OAP} to (Exp_{OAP} + $Exp_{AS/SHS}$).

Relative risk and proportional PAF approach

For GBD 2017 we developed a new approach to use the IER for obtaining PAFs for both OAP and HAP. Previously, relative risks for both exposures were obtained from the IER as a function of exposure and relative to the same TMREL. In reality, were a country to reduce only one of these risk factors, the other would remain. We failed to consider the joint effects of particulate matter from outdoor exposure and burning solid fuels for cooking.

In GBD 2017, relative risks were still estimated from the output of the IER curve. Everyone is exposed to some level of OAP, but only a proportion of the population in each location-year use solid cooking fuel and are exposed to HAP. For the proportion of the population not exposed to HAP the relative risk was obtained by $RR_{OAP} = IER(z = Exp_{OAP})$ and used to calculate the PAF for each location based on the population-weighted exposure.

For the proportion of the population exposed to both OAP and HAP, we calculated a joint relative risk from the IER by $RR_{OAP+HAP} = IER(z = Exp_{OAP}+Exp_{HAP})$. This joint relative risk is used to calculate a joint PAF for each location. PAF calculation is detailed in the methods appendix. For each location, we proportioned the joint PAF based on the proportion of exposure due to OAP and HAP respectively. See the table below for equations used to calculate proportional PAFs.

PAF	Population not exposed to HAP	Population exposed to HAP

OAP	PAF_{OAP}	$(Exp_{OAP}/(Exp_{OAP}+Exp_{HAP}))*PAF_{OAP+HAP}$
HAP	0	$(Exp_{HAP}/(Exp_{OAP}+Exp_{HAP}))*PAF_{OAP+HAP}$

Generally, as expected, this new strategy led to lower PAFs for both ambient and household particulate matter pollution.

References

1. van Donkelaar, A.; Martin, R. V; Brauer, M.; Hsu, N. C.; Kahn, R. A.; Levy, R. C.; Lyapustin, A.; Sayer, A. M.; Winker, D. M. Global Estimates of Fine Particulate Matter using a Combined Geophysical-Statistical Method with Information from Satellites, Models, and Monitors. Environ. Sci. Technol. 2016, 50 (7), 3762–3772
2. Shaddick, G., Thomas, M.L., Jobling, A., Brauer, M., van Donkelaar, A., Burnett, R., Chang, H., Cohen, A., Van Dingenen, R., Dora, C. and Gumy, S., 2016. Data Integration Model for Air Quality: A Hierarchical Approach to the Global Estimation of Exposures to Ambient Air Pollution. Journal of Royal Statistical Society Series C (Applied Statistics). 2017. DOI: 10.1111/rssc.12227
3. Brauer, M.; Freedman, G.; Frostad, J.; van Donkelaar, A.; Martin, R. V; Dentener, F.; Van Dingenen, R.; Estep, K.; Amini, H.; Apte, J. S.; et al. Ambient Air Pollution Exposure Estimation for the Global Burden of Disease 2013. Environ. Sci. Technol. 2015, 50 (1), 79–88.
4. Shaddick G, Thomas M, Amini H, Broday DM, Cohen A, Frostad J, Green A, Gumy S, Liu Y, Martin RV, Prüss-Üstün A, Simpson D, van Donkelaar A, Brauer M. Data integration for the assessment of population exposure to ambient air pollution for global burden of disease assessment. Environ Sci Technol. 2018 Jun 29. doi: 10.1021/acs.est.8b02864
5. Rue, H.; Martino, S.; Chopin, N.; Approximate Bayesian inference for latent Gaussian models by using integrated nested Laplace approximations. Journal of the royal statistical society: Series b (statistical methodology). 2009;71(2):319-92.
6. Cleveland, W.S. and Devlin, S.J., 1988. Locally weighted regression: an approach to regression analysis by local fitting. Journal of the American statistical association, 83(403), pp.596-610.
7. Turner MC,Jerrett M, Pope CA 3rd, Krewski D, Gapstur SM, Diver WR, Beckerman BS, Marshall JD, Su J, Crouse DL, Burnett RT. Long-term ozone exposure and mortality in a large prospective study . Am J Respir Crit Care Med. 2016; 193(10): 1134-42.
8. Bowe B, Xie Y, Li T, Yan Y, Xian H, Al-Aly Z. The 2016 global and national burden of diabetes mellitus attributable to PM2.5 air pollution. *The Lancet Planetary Health*. 2018; 2(7): e301–12.
9. Cohen AJ, Brauer M, Burnett R, et al. Estimates and 25-year trends of the global burden of disease attributable to ambient air pollution: an analysis of data from the Global Burden of Diseases Study 2015. Lancet 2017; published online April 10. http://dx.doi.org/10.1016/S0140-6736(17)30505-6.
10. Burnett RT, Pope CA 3rd, Ezzati M, Olives C, Lim SS, Mehta S, Shin HH, Singh G, Hubbell B, Brauer M, Anderson HR, Smith KR, Balmes JR, Bruce NG, Kan H, Laden F, Prüss-Ustün A, Turner MC, Gapstur SM, Diver WR, Cohen A. An integrated risk function for estimating the global burden of disease attributable to ambient fine particulate matter exposure. *Environ Health Perspect*. 2014; 122(4): 397-403.

11. Yin P, Brauer M, Cohen A, Burnett RT, Liu J, Liu Y, Liang R, Wang W, Qi J, Wang L, Zhou M. Long-term Fine Particulate Matter Exposure and Nonaccidental and Cause-specific Mortality in a Large National Cohort of Chinese Men. *Environ Health Perspect*. 2017; 125(11): 117002.
12. Xiaomin Wei, Meng E., Sufang Yu. A meta-analysis of passive smoking and risk of developing Type 2 Diabetes Mellitus. *Diabetes Research and Clinical Practice*. 2015; 107(1): 9-14.

Household Air Pollution Capstone Appendix

Flowchart

Household Air Pollution from Solid Fuels

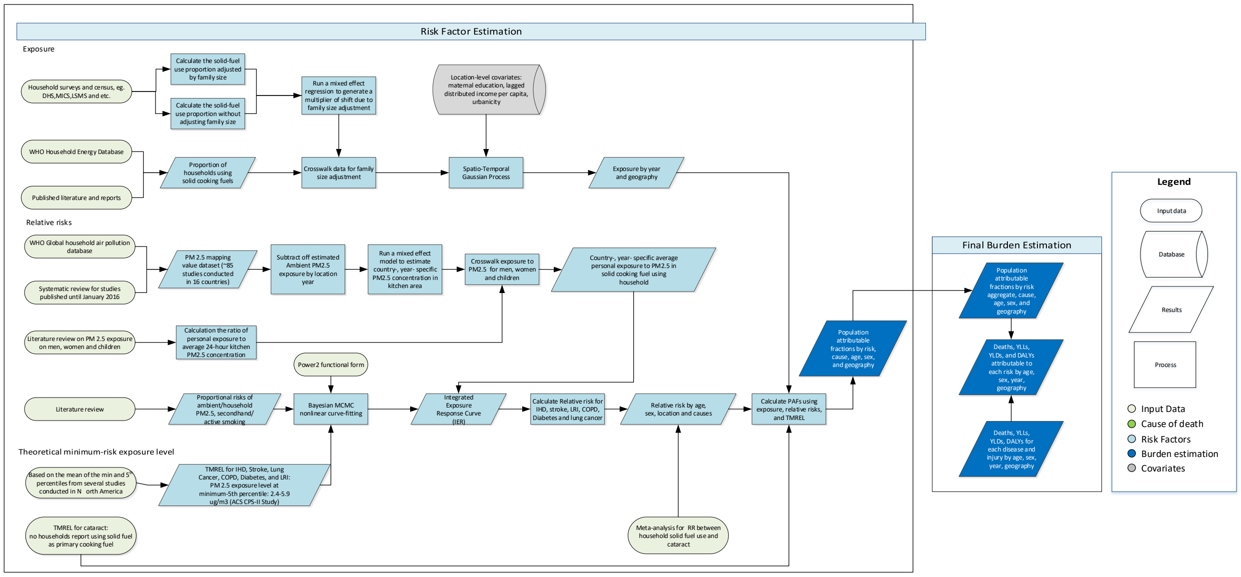

Input Data & Methodological Summary

Exposure

Case definition

Exposure to household air pollution from solid fuels (HAP) is defined as the proportion of households using solid cooking fuels. The definition of solid fuel in our analysis includes coal, wood, charcoal, dung, and agricultural residues.

Input data

Data were extracted from the standard multi-country survey series such as Demographic and Health Surveys (DHS), Living Standards Measurement Surveys (LSMS), Multiple Indicator Cluster Surveys (MICS), and World Health Surveys (WHS), as well as country-specific survey series such as Kenya Welfare Monitoring Survey and South Africa General Household Survey. To fill the gaps of data in surveys and censuses, we also downloaded and updated HAP estimates from WHO Energy Database and extracted from literature through systematic review. Each nationally or sub-nationally representative data point provided an estimate for the percentage of households using solid cooking fuels. Estimates for the usage of solid fuels for non-cooking purpose were excluded, i.e. primary fuels for lighting. The database, with

estimates from 1980 to 2017, contained about 680 studies from 150 countries. As updates to systematic reviews are performed on an ongoing schedule across all GBD causes and risk factors, an update for household air pollution will be performed in the next 1-2 iterations.

Modelling strategy

Household air pollution was modelled at household level using a three-step modelling strategy that uses linear regression, spatiotemporal regression and Gaussian Process Regression (GPR). The first step is a mixed-effect linear regression of logit-transformed proportion of households using solid cooking fuels. The linear model contains maternal education, proportion of population living in urban areas, and lagged-distributed income as covariates and has nested random effect by GBD region, and GBD super region respectively. The full ST-GPR process is specified elsewhere this appendix. No substantial modelling changes were made in this round compared to GBD 2016.

Theoretical minimum-risk exposure level

For cataract, the TMREL is defined as no households using solid cooking fuel. For outcomes that utilise evidence based on the Integrated Exposure Response (IER), the TMREL is defined as uniform distribution between 2.4 and 5.9 ug/m^3.

Relative risks

In addition to the previously included outcomes of lower respiratory infections (LRI), stroke, Ischemic Heart Disease (IHD), Chronic Obstructive Pulmonary Disease (COPD), lung cancer, and cataract, in GBD 2017 we added Type II Diabetes as a new outcome of household air pollution. The relative risk for cataracts was extracted from a meta-analysis and is 2.47 with 95% (1.61, 3.73).[1] GBD currently only estimates cataracts as an outcome for females.

In GBD 2017, we adopted a new approach for risk attribution using the Integrated Exposure-Response Function (IER). Updates to the IER and the new joint-estimation PAF approach is described in the Ambient Particulate Matter appendix.

$PM_{2.5}$ mapping value

In order to use the IER curve, we must estimate the exposure to particulate matter with diameter of less than 2.5 micrometers ($PM_{2.5}$). Since GBD 2015 we have been using a mapping model relying on a database of now almost 90 studies which measures $PM_{2.5}$ exposure in households using solid cooking fuel. Using socio-demographic index and study-level factors as covariates, we predict exposure for all location-years.

In GBD 2017, we updated the model to estimate the individual exposure to $PM_{2.5}$ over and above ambient levels due to the use of solid cooking fuel. We did this by subtracting off the estimated ambient level $PM_{2.5}$ for the location-year of each study in the database before inputting them into the model. By doing this we have independent estimates for $PM_{2.5}$ exposure due to ambient and household solid fuel use.

These exposures are cross-walked to values for men, women, and children by generating the ratio of each group's mean exposure to the overall mean personal exposure. The resulting location, year, sex, and age specific $PM_{2.5}$ exposure values are used as inputs in the IER and attributable burden calculation process.

References

1. Smith KR, Bruce N, Balakrishnan K, Adair-Rohani H, Balmes J, Chafe Z, et al. Millions Dead: How Do We Know and What Does It Mean? Methods Used in the Comparative Risk Assessment of Household Air Pollution. Annu Rev Public Health. 2014;35(1):185–206.

Online Results Appendix - Supplementary tables and figures

Supplementary Table 1: Estimated disability weights and 95% uncertainty intervals for GBD chronic respiratory disease states

Chronic respiratory disease states	DW and 95% U.I.
**Asthma**	
Controlled	0·015 (0·007–0·026)
Partly controlled	0·036 (0·022–0·055)
Uncontrolled	0·133 (0·086–0·192)
**COPD**	
Mild	0·019 (0·011–0·033)
Moderate	0·225 (0·153–0·310)
Severe	0·408 (0·273–0·556)
**Interstitial lung disease and pulmonary sarcoidosis**	
Asymptomatic interstitial lung disease and pulmonary sarcoidosis	-
Moderate heart failure due to severe interstitial lung disease and pulmonary sarcoidosis	-
Moderate interstitial lung disease and pulmonary sarcoidosis	0.225 (0.153-0.310)
Mild interstitial lung disease and pulmonary sarcoidosis	0.019 (0.011-0.033)
Treated heart failure due to severe interstitial lung disease and pulmonary sarcoidosis	-
Severe interstitial lung disease and pulmonary sarcoidosis without heart failure	0.408 (0.273-0.556)
Severe heart failure due to severe interstitial lung disease and pulmonary sarcoidosis	-
Mild heart failure due to severe interstitial lung disease and pulmonary sarcoidosis	-
**Pneumoconiosis**	
Severe asbestosis without heart failure	0·408 (0·273-0·556)
Moderate coal workers pneumoconiosis	0·225 (0·153-0·310)
Asymptomatic coal workers pneumoconiosis	-
**Other chronic respiratory diseases**	
None	-

Supplementary Table 2: Age-standardized prevalence of all and specific CRDs, by sex and by GBD super regions, in 1990 and in 2017

All Chronic Respiratory Diseases			
**1990**		**Sex**	
**Super Region**	**Male**	**Female**	**Both**
Global	7.9264% (7.4407% to 7.4407%)	8.4112% (7.8897% to 7.8897%)	8.1577% (7.6640% to 7.6640%)
Central Europe, Eastern Europe, and Central Asia	7.5947% (7.0967% to 7.0967%)	9.4940% (8.9235% to 8.9235%)	8.7069% (8.1780% to 8.1780%)
High-income	8.7133% (8.1172% to 8.1172%)	8.9053% (8.2867% to 8.2867%)	8.7688% (8.1639% to 8.1639%)
Latin America and Caribbean	8.6641% (7.8873% to 7.8873%)	9.5266% (8.7078% to 8.7078%)	9.1117% (8.3349% to 8.3349%)
North Africa and Middle East	8.5614% (7.9643% to 7.9643%)	9.5604% (8.9122% to 8.9122%)	9.0442% (8.4317% to 8.4317%)
South Asia	7.4014% (6.9244% to 6.9244%)	7.7479% (7.2400% to 7.2400%)	7.5663% (7.0794% to 7.0794%)
Southeast Asia, East Asia, and Oceania	8.1277% (7.5919% to 7.5919%)	8.2661% (7.7135% to 7.7135%)	8.1853% (7.6588% to 7.6588%)
Sub-Saharan Africa	6.8130% (6.3223% to 6.3223%)	7.3910% (6.8681% to 6.8681%)	7.1038% (6.6006% to 6.6006%)
**2017**		**Sex**	
**Super Region**	**Male**	**Female**	**Both**
Global	6.7311% (6.2483% to 6.2483%)	7.2669% (6.7418% to 6.7418%)	6.9915% (6.4967% to 6.4967%)
Central Europe, Eastern Europe, and Central Asia	6.4121% (5.9300% to 5.9300%)	7.9599% (7.3677% to 7.3677%)	7.2612% (6.7288% to 6.7288%)
High-income	7.9761% (7.4177% to 7.4177%)	8.4359% (7.8320% to 7.8320%)	8.1902% (7.6270% to 7.6270%)
Latin America and Caribbean	7.1614% (6.4350% to 6.4350%)	7.8878% (7.1281% to 7.1281%)	7.5388% (6.7952% to 6.7952%)
North Africa and Middle East	8.1221% (7.4635% to 7.4635%)	8.9565% (8.2871% to 8.2871%)	8.5116% (7.8521% to 7.8521%)
South Asia	6.3472% (5.8956% to 5.8956%)	6.8561% (6.3790% to 6.3790%)	6.5961% (6.1494% to 6.1494%)
Southeast Asia, East Asia, and Oceania	6.5486% (5.9862% to 5.9862%)	6.8423% (6.2880% to 6.2880%)	6.6884% (6.1362% to 6.1362%)
Sub-Saharan Africa	6.2645% (5.7587% to 5.7587%)	6.6466% (6.1036% to 6.1036%)	6.4602% (5.9311% to 5.9311%)

Chronic Obstructive Pulmonary Disease			
**1990**		**Sex**	
**Super Region**	**Male**	**Female**	**Both**
Global	4.6184% (4.2566% to 4.2566%)	4.7453% (4.3538% to 4.3538%)	4.6692% (4.2967% to 4.2967%)
Central Europe, Eastern Europe, and Central Asia	4.3386% (3.9709% to 3.9709%)	6.2257% (5.7077% to 5.7077%)	5.4604% (5.0121% to 5.0121%)
High-income	3.7703% (3.5095% to 3.5095%)	3.1548% (2.9005% to 2.9005%)	3.4044% (3.1494% to 3.1494%)
Latin America and Caribbean	4.1647% (3.8292% to 3.8292%)	4.3363% (3.9998% to 3.9998%)	4.2571% (3.9194% to 3.9194%)
North Africa and Middle East	3.9473% (3.5518% to 3.5518%)	4.2454% (3.8209% to 3.8209%)	4.0875% (3.6873% to 3.6873%)
South Asia	5.2940% (4.7717% to 4.7717%)	5.7300% (5.1433% to 5.1433%)	5.5029% (4.9452% to 4.9452%)
Southeast Asia, East Asia, and Oceania	5.3922% (4.9547% to 4.9547%)	5.4697% (4.9888% to 4.9888%)	5.4204% (4.9762% to 4.9762%)
Sub-Saharan Africa	3.5668% (3.1826% to 3.1826%)	3.6565% (3.2597% to 3.2597%)	3.6125% (3.2258% to 3.2258%)

**2017**		**Sex**	
**Super Region**	**Male**	**Female**	**Both**
Global	3.7115% (3.3347% to 3.3347%)	3.7914% (3.4310% to 3.4310%)	3.7407% (3.3698% to 3.3698%)
Central Europe, Eastern Europe, and Central Asia	3.4994% (3.1142% to 3.1142%)	4.8336% (4.3189% to 4.3189%)	4.2384% (3.7878% to 3.7878%)
High-income	3.6420% (3.2839% to 3.2839%)	3.1802% (2.8910% to 2.8910%)	3.3823% (3.0597% to 3.0597%)
Latin America and Caribbean	3.2977% (2.9504% to 2.9504%)	3.4154% (3.0553% to 3.0553%)	3.3617% (3.0088% to 3.0088%)
North Africa and Middle East	3.8648% (3.4844% to 3.4844%)	3.7664% (3.3778% to 3.3778%)	3.8022% (3.4299% to 3.4299%)
South Asia	4.4406% (3.9645% to 3.9645%)	4.5743% (4.0891% to 4.0891%)	4.5060% (4.0322% to 4.0322%)
Southeast Asia, East Asia, and Oceania	3.4654% (3.0944% to 3.0944%)	3.7487% (3.3834% to 3.3834%)	3.5975% (3.2463% to 3.2463%)
Sub-Saharan Africa	3.1299% (2.7856% to 2.7856%)	3.0376% (2.6963% to 2.6963%)	3.0823% (2.7339% to 2.7339%)

Asthma			
**1990**		**Sex**	
**Super Region**	**Male**	**Female**	**Both**
Global	3.7984% (3.3866% to 3.3866%)	4.1687% (3.7231% to 3.7231%)	3.9817% (3.5487% to 3.5487%)
Central Europe, Eastern Europe, and Central Asia	3.5798% (3.2102% to 3.2102%)	3.9108% (3.4980% to 3.4980%)	3.7601% (3.3722% to 3.3722%)
High-income	5.3935% (4.8275% to 4.8275%)	6.1679% (5.5238% to 5.5238%)	5.7895% (5.1893% to 5.1893%)
Latin America and Caribbean	4.8983% (4.1469% to 4.1469%)	5.6691% (4.8915% to 4.8915%)	5.2954% (4.5387% to 4.5387%)
North Africa and Middle East	5.1298% (4.5675% to 4.5675%)	5.9042% (5.2827% to 5.2827%)	5.5075% (4.9099% to 4.9099%)
South Asia	2.8451% (2.5647% to 2.5647%)	2.7576% (2.4994% to 2.4994%)	2.8016% (2.5322% to 2.5322%)
Southeast Asia, East Asia, and Oceania	3.2752% (2.8709% to 2.8709%)	3.2953% (2.8891% to 2.8891%)	3.2816% (2.8777% to 2.8777%)
Sub-Saharan Africa	3.5263% (3.1486% to 3.1486%)	4.0621% (3.6349% to 3.6349%)	3.7956% (3.4048% to 3.4048%)
**2017**		**Sex**	
**Super Region**	**Male**	**Female**	**Both**
Global	3.3617% (2.9519% to 2.9519%)	3.8339% (3.3948% to 3.3948%)	3.5996% (3.1751% to 3.1751%)
Central Europe, Eastern Europe, and Central Asia	3.1064% (2.7434% to 2.7434%)	3.4958% (3.0747% to 3.0747%)	3.3137% (2.9137% to 2.9137%)
High-income	4.6907% (4.1859% to 4.1859%)	5.6379% (5.0571% to 5.0571%)	5.1745% (4.6563% to 4.6563%)
Latin America and Caribbean	4.1262% (3.4397% to 3.4397%)	4.7876% (4.0826% to 4.0826%)	4.4677% (3.7810% to 3.7810%)
North Africa and Middle East	4.7146% (4.1267% to 4.1267%)	5.6760% (5.0443% to 5.0443%)	5.1783% (4.5833% to 4.5833%)
South Asia	2.4506% (2.1784% to 2.1784%)	2.8091% (2.4925% to 2.4925%)	2.6256% (2.3376% to 2.3376%)
Southeast Asia, East Asia, and Oceania	3.3816% (2.8873% to 2.8873%)	3.3895% (2.9149% to 2.9149%)	3.3871% (2.9104% to 2.9104%)
Sub-Saharan Africa	3.3470% (2.9231% to 2.9231%)	3.8464% (3.3850% to 3.3850%)	3.6035% (3.1601% to 3.1601%)

Interstitial Lung Disease and Pulmonary Sarcoidosis

**1990**		**Sex**	
**Super Region**	**Male**	**Female**	**Both**
Global	0.0880% (0.0803% to 0.0803%)	0.0737% (0.0672% to 0.0672%)	0.0799% (0.0729% to 0.0729%)
Central Europe, Eastern Europe, and Central Asia	0.2264% (0.2041% to 0.2041%)	0.1609% (0.1450% to 0.1450%)	0.1897% (0.1705% to 0.1705%)
High-income	0.1087% (0.0990% to 0.0990%)	0.0851% (0.0778% to 0.0778%)	0.0948% (0.0867% to 0.0867%)
Latin America and Caribbean	0.0530% (0.0481% to 0.0481%)	0.0431% (0.0392% to 0.0392%)	0.0479% (0.0436% to 0.0436%)
North Africa and Middle East	0.0300% (0.0271% to 0.0271%)	0.0387% (0.0349% to 0.0349%)	0.0343% (0.0310% to 0.0310%)
South Asia	0.0536% (0.0479% to 0.0479%)	0.0507% (0.0455% to 0.0455%)	0.0520% (0.0466% to 0.0466%)
Southeast Asia, East Asia, and Oceania	0.0601% (0.0540% to 0.0540%)	0.0473% (0.0424% to 0.0424%)	0.0528% (0.0475% to 0.0475%)
Sub-Saharan Africa	0.1022% (0.0912% to 0.0912%)	0.0986% (0.0883% to 0.0883%)	0.1000% (0.0894% to 0.0894%)

**2017**		**Sex**	
**Super Region**	**Male**	**Female**	**Both**
Global	0.0869% (0.0787% to 0.0787%)	0.0713% (0.0647% to 0.0647%)	0.0782% (0.0709% to 0.0709%)
Central Europe, Eastern Europe, and Central Asia	0.1829% (0.1628% to 0.1628%)	0.1280% (0.1135% to 0.1135%)	0.1527% (0.1356% to 0.1356%)
High-income	0.1251% (0.1143% to 0.1143%)	0.0994% (0.0912% to 0.0912%)	0.1110% (0.1019% to 0.1019%)
Latin America and Caribbean	0.0503% (0.0455% to 0.0455%)	0.0430% (0.0388% to 0.0388%)	0.0465% (0.0420% to 0.0420%)
North Africa and Middle East	0.0334% (0.0300% to 0.0300%)	0.0381% (0.0343% to 0.0343%)	0.0358% (0.0322% to 0.0322%)
South Asia	0.0586% (0.0523% to 0.0523%)	0.0562% (0.0504% to 0.0504%)	0.0571% (0.0510% to 0.0510%)
Southeast Asia, East Asia, and Oceania	0.0744% (0.0665% to 0.0665%)	0.0557% (0.0497% to 0.0497%)	0.0641% (0.0574% to 0.0574%)
Sub-Saharan Africa	0.0995% (0.0883% to 0.0883%)	0.0949% (0.0846% to 0.0846%)	0.0967% (0.0862% to 0.0862%)

Pneumonconiosis			
**1990**		**Sex**	
**Super Region**	**Male**	**Female**	**Both**
Global	0.0131% (0.0116% to 0.0116%)	0.0020% (0.0017% to 0.0017%)	0.0070% (0.0062% to 0.0062%)
Central Europe, Eastern Europe, and Central Asia	0.0114% (0.0102% to 0.0102%)	0.0019% (0.0017% to 0.0017%)	0.0056% (0.0050% to 0.0050%)
High-income	0.0061% (0.0054% to 0.0054%)	0.0009% (0.0007% to 0.0007%)	0.0031% (0.0027% to 0.0027%)
Latin America and Caribbean	0.0093% (0.0082% to 0.0082%)	0.0021% (0.0018% to 0.0018%)	0.0055% (0.0048% to 0.0048%)
North Africa and Middle East	0.0062% (0.0053% to 0.0053%)	0.0024% (0.0021% to 0.0021%)	0.0044% (0.0037% to 0.0037%)
South Asia	0.0045% (0.0039% to 0.0039%)	0.0017% (0.0014% to 0.0014%)	0.0031% (0.0028% to 0.0028%)
Southeast Asia, East Asia, and Oceania	0.0290% (0.0251% to 0.0251%)	0.0031% (0.0025% to 0.0025%)	0.0149% (0.0129% to 0.0129%)
Sub-Saharan Africa	0.0046% (0.0040% to 0.0040%)	0.0019% (0.0016% to 0.0016%)	0.0032% (0.0028% to 0.0028%)
**2017**		**Sex**	
**Super Region**	**Male**	**Female**	**Both**
Global	0.0126% (0.0111% to 0.0111%)	0.0016% (0.0014% to 0.0014%)	0.0066% (0.0059% to 0.0059%)
Central Europe, Eastern Europe, and Central Asia	0.0085% (0.0075% to 0.0075%)	0.0017% (0.0015% to 0.0015%)	0.0045% (0.0040% to 0.0040%)
High-income	0.0056% (0.0050% to 0.0050%)	0.0008% (0.0007% to 0.0007%)	0.0030% (0.0027% to 0.0027%)
Latin America and Caribbean	0.0078% (0.0068% to 0.0068%)	0.0018% (0.0015% to 0.0015%)	0.0046% (0.0040% to 0.0040%)
North Africa and Middle East	0.0067% (0.0057% to 0.0057%)	0.0024% (0.0020% to 0.0020%)	0.0046% (0.0039% to 0.0039%)
South Asia	0.0039% (0.0034% to 0.0034%)	0.0016% (0.0014% to 0.0014%)	0.0028% (0.0024% to 0.0024%)
Southeast Asia, East Asia, and Oceania	0.0261% (0.0226% to 0.0226%)	0.0018% (0.0016% to 0.0016%)	0.0130% (0.0113% to 0.0113%)
Sub-Saharan Africa	0.0044% (0.0038% to 0.0038%)	0.0018% (0.0015% to 0.0015%)	0.0030% (0.0026% to 0.0026%)

Online Figure 1. World map with the GBD regions and super-regions

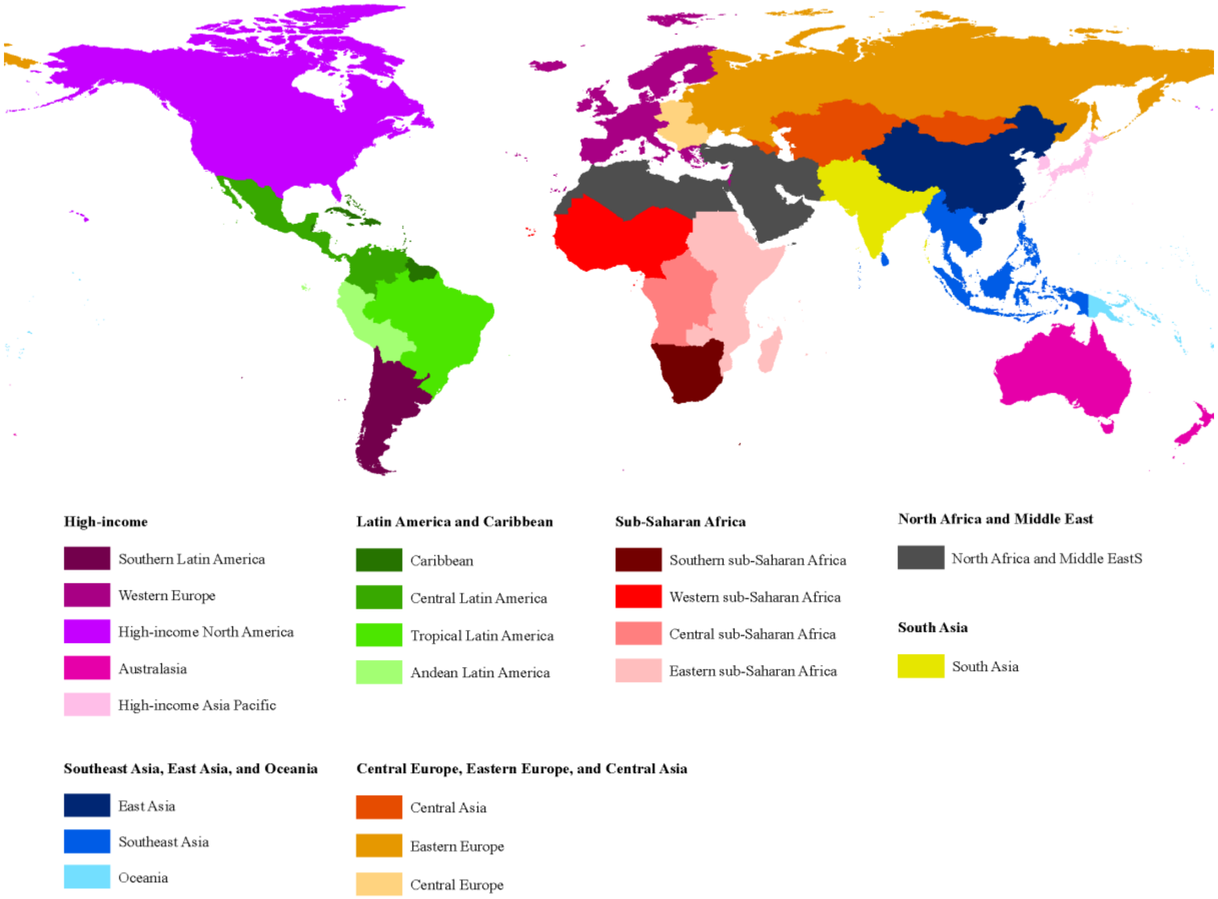

Online Figure 2a. Variations in CRD-attributable YLDs by sex and in aggregate across GBD super regions in 1990

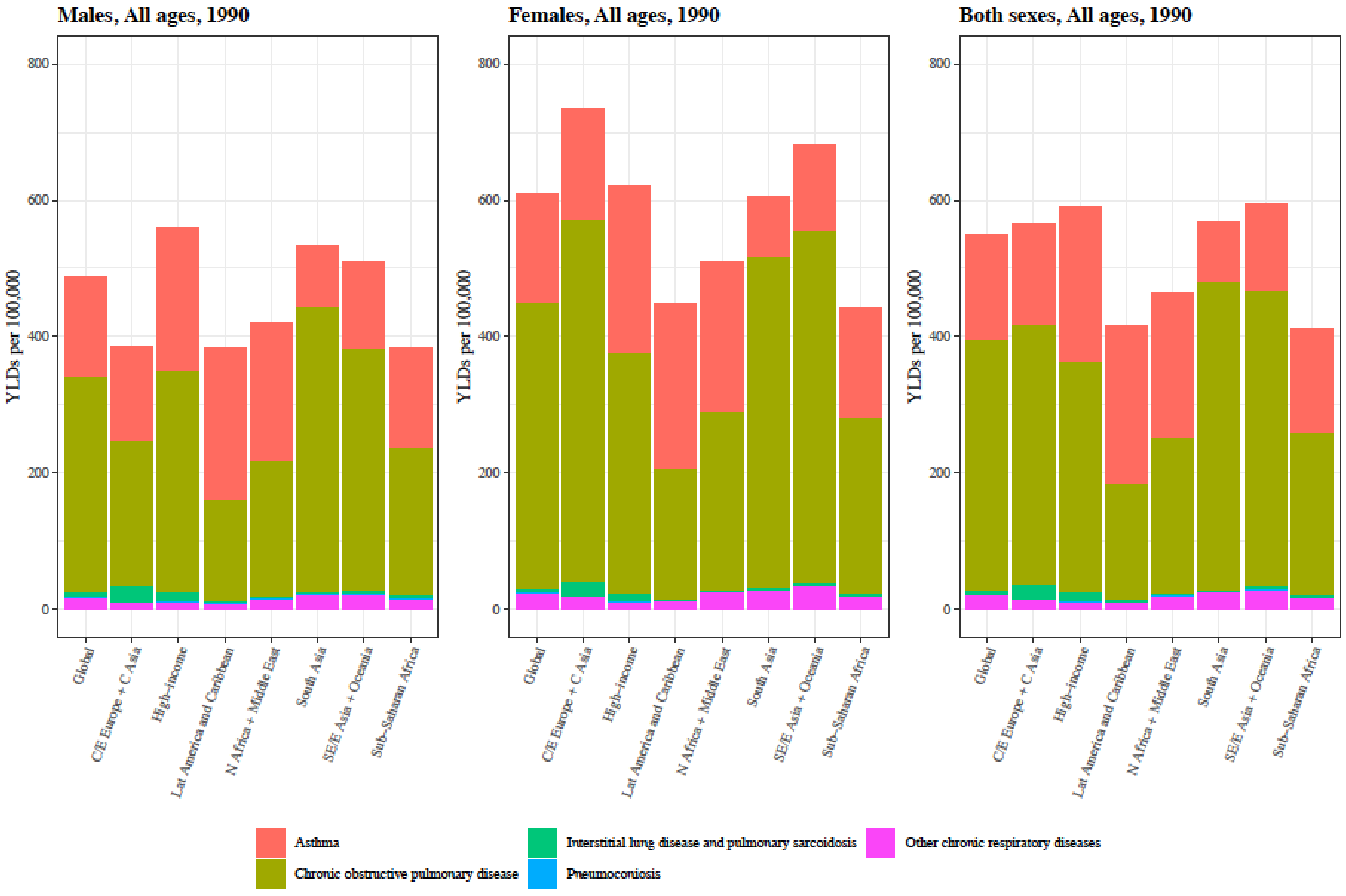

Online Figure 2b. Variations in CRD-attributable YLLs by sex and in aggregate across GBD super regions in 1990

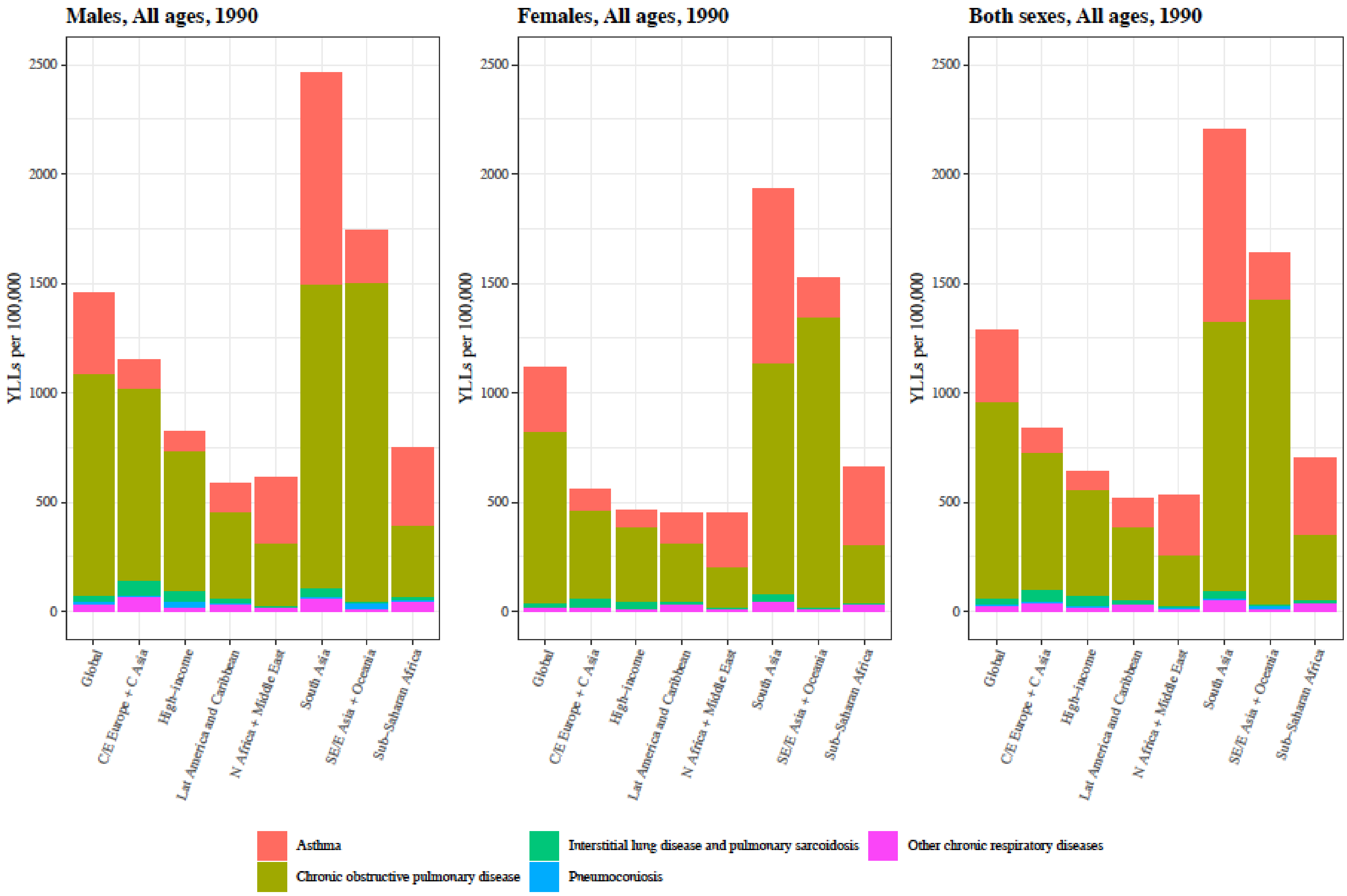

Online Figure 2c. Variations in CRD-attributable DALYs by sex and in aggregate across GBD super regions in 1990

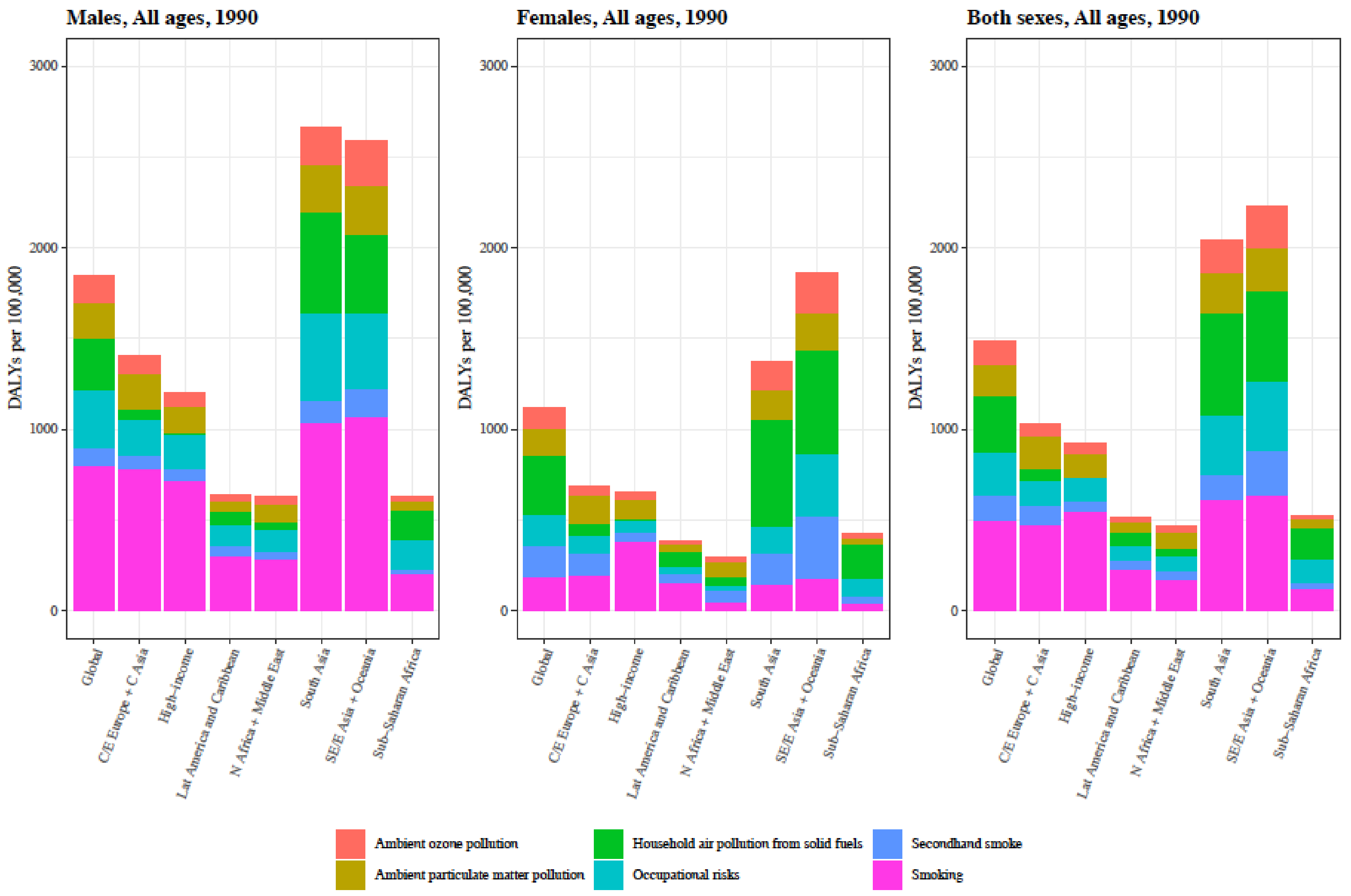

Supplement: Supplementary appendix 1 [file mmc1.pdf]
